# ESRRG-controlled downregulation of KCNN1 in primary sensory neurons is required for neuropathic pain

**DOI:** 10.1172/jci.insight.180085

**Published:** 2024-05-21

**Authors:** Huixing Wang, Wanhong Zuo, Xiaozhou Feng, Xiaodong Huo, Yingping Liang, Bing Wang, Dilip Sharma, Xiang Li, Bushra Yasin, Jiang-Hong Ye, Huijuan Hu, Yuan-Xiang Tao

**Affiliations:** 1 Department of Anesthesiology;; 2 Department of Physiology, Pharmacology & Neuroscience; and; 3 Department of Cell Biology & Molecular Medicine, New Jersey Medical School, Rutgers, The State University of New Jersey, Newark, New Jersey, USA.

**Keywords:** Neuroscience, Pain

## Abstract

Peripheral nerve injury–induced neuronal hyperactivity in the dorsal root ganglion (DRG) participates in neuropathic pain. The calcium-activated potassium channel subfamily N member 1 (KCNN1) mediates action potential afterhyperpolarization (AHP) and gates neuronal excitability. However, the specific contribution of DRG KCNN1 to neuropathic pain is not yet clear. We report that chronic constriction injury (CCI) of the unilateral sciatic nerve or unilateral ligation of the fourth lumbar nerve produced the downregulation of *Kcnn1* mRNA and KCNN1 protein in the injured DRG. This downregulation was partially attributed to a decrease in DRG estrogen-related receptor gamma (ESRRG), a transcription factor, which led to reduced binding to the *Kcnn1* promoter. Rescuing this downregulation prevented CCI-induced decreases in total potassium voltage currents and AHP currents, reduced excitability in the injured DRG neurons, and alleviated CCI-induced development and maintenance of nociceptive hypersensitivities, without affecting locomotor function and acute pain. Mimicking the CCI-induced DRG KCNN1 downregulation resulted in augmented responses to mechanical, heat, and cold stimuli in naive mice. Our findings indicate that ESRRG-controlled downregulation of DRG KCNN1 is likely essential for the development and maintenance of neuropathic pain. Thus, KCNN1 may serve as a potential target for managing this disorder.

## Abstract 2

Peripheral nerve injury-induced KCNN1 downregulation contributes to neuropathic pain via decreased action potential afterhyperpolarization and total Kv currents and increased excitability in the injured neurons of dorsal root ganglion.

## Introduction

Neuropathic pain is a chronic and refractory clinical disorder affecting 7%–10% of the world’s population (1). Its clinical characteristics include ongoing or intermittent spontaneous pain (such as shooting, pricking, burning, pins, needles, and freezing pain), allodynia (pain due to innocuous stimuli), and hyperalgesia (enhanced pain in responses to noxious stimuli). Current successful treatments for these nociceptive hypersensitivities are highly limited (1, 2). Despite taking prescribed medications, including opioids and nonopioids (e.g., duloxetine, amitriptyline, and gabapentin), more than 50% of patients with neuropathic pain still report moderate or severe pain (1, 2). Moreover, the severe side effects of these medications significantly affect long-term adherence (3–6). This creates an evident and urgent need to identify new targets and mechanisms for managing neuropathic pain.

Abnormal ectopic discharges and hyperexcitability in the neuronal soma of the dorsal root ganglion (DRG) and neuroma at the injured site following peripheral nerve injury are potential mechanisms underlying the genesis of neuropathic pain (7, 8). The calcium-activated potassium channel subfamily N member 1 (KCNN1) is a member of the small conductance calcium-activated K^+^ channels that play a role in mediating action potential afterhyperpolarization (AHP), thereby modulating neuronal excitability (9). Single-cell RNA-sequencing analysis showed that *Kcnn1* mRNA was expressed at moderate to low levels in MrgD-positive nonpeptidergic nociceptor, C-low-threshold mechanoreceptor (LTMR), Aδ-LTMR, calcitonin gene-related peptide–positive (CGRP-positive) peptidergic nociceptor, Aβ rapidly adapting–LTMR, Aβ slowly adapting type 1–LTMR, Aβ field–LTMR, and proprioceptor in the DRGs (10–12). Immunohistochemistry studies revealed that KCNN1 was preferentially localized to small and medium neuronal bodies (less than 1,000 μm^2^) in the DRGs, in which about 87% and 56% of KCNN1-positive DRG neurons were labeled by the lectin isolectin B4 (IB4) (a marker for nonpeptidergic nociceptor) and CGRP (a marker for peptidergic nociceptor), respectively (13). Early electrophysiologic recording showed that Aδ or Aα/β DRG neurons expressing CGRP or substance P (SP) exhibited a significantly longer mean AHP duration (14, 15). Further studies reported that IB4-labeled DRG neurons, whether expressing or not expressing CGRP or SP, exhibited a prolonged AHP duration, while IB4-negative DRG neurons expressing or not expressing CGRP or SP displayed a short AHP duration (16). The evidence indicates that the prolonged AHP duration in both nonpeptidergic and peptidergic DRG neurons may be associated with high expression of KCNN1 in these neurons. Furthermore, peripheral nerve injury reduced the apamin-sensitive (a selective KCNN blocker) potassium voltage (Kv) currents in injured rat DRG neurons (17), aligning with a clinical observation that found reduced KCNN1 level in the DRGs avulsed from patients with neuropathic pain (18). However, a previous report showed unchanged KCNN1 expression in injured rat DRGs 15 days after chronic constriction injury (CCI) of unilateral sciatic nerve (13). Therefore, the role of DRG KCNN1 in neuropathic pain remains elusive.

In this study, we first verified the cellular distribution patterns of KCNN1 in mouse DRG tissues. We then investigated whether CCI or fourth lumbar (L4) spinal nerve ligation (SNL) led to KCNN1 downregulation in the injured DRG. We also examined whether this downregulation was required for the CCI-induced DRG neuronal hyperexcitability and the development and maintenance of CCI-induced nociceptive hypersensitivity. Finally, we aimed to uncover how CCI caused KCCN1 downregulation in the injured DRG.

## Results

### Cellular distribution of Kcnn1 mRNA in the DRG.

**Figure 1:**
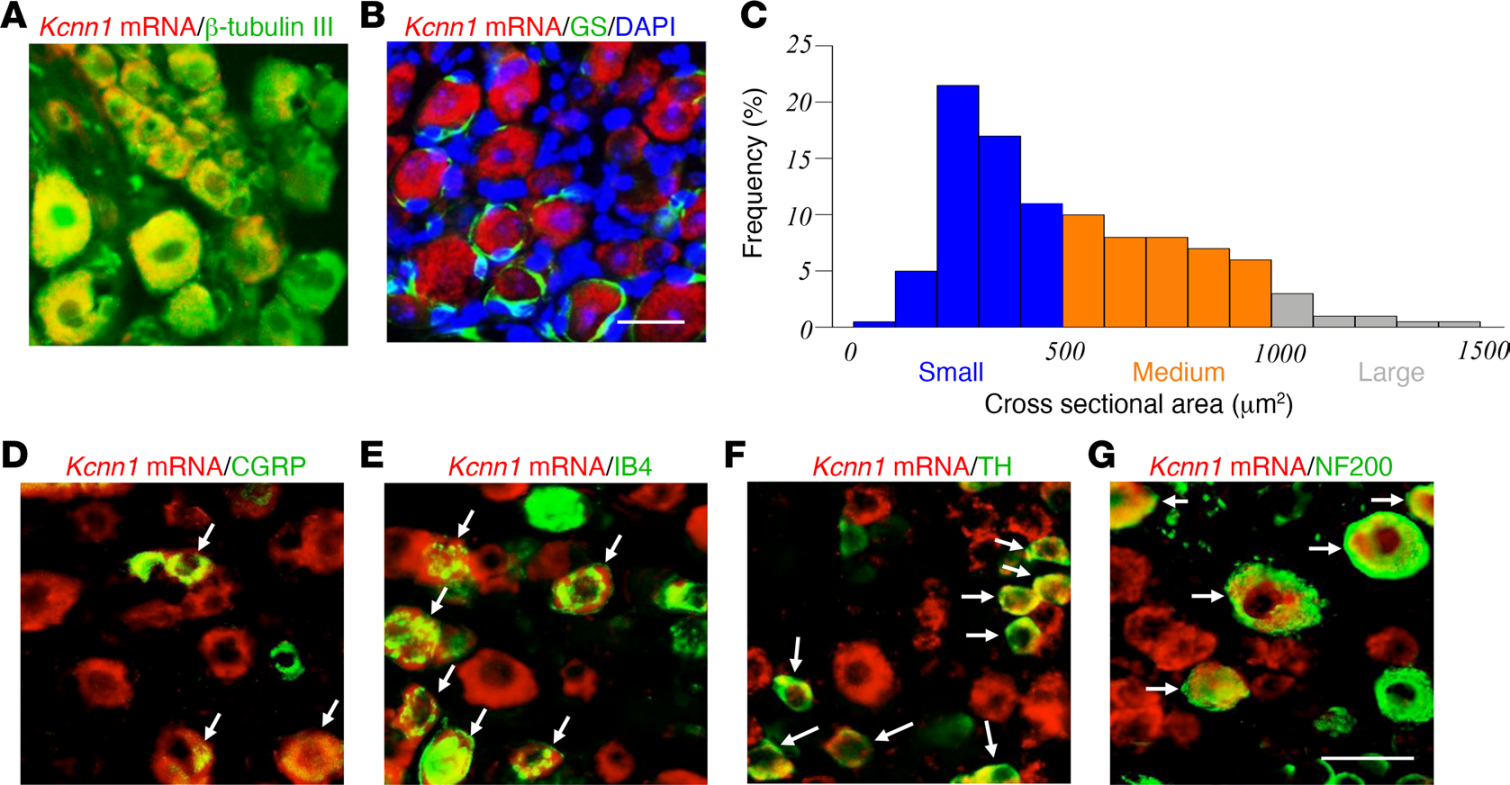
Cellular distribution of *Kcnn1* mRNA in the lumbar DRGs of naive mice. (**A** and **B**) Representative images of in situ hybridization histochemistry (ISHH) for *Kcnn1* mRNA (shown in red) and immunohistochemistry for β-tubulin III (shown in green; **A**) or glutamine synthetase (GS; shown in green; **B**) in the L3/4 DRGs. The cellular nucleus was labeled by 4′,6-diamidino-2-phenylindole (DAPI; shown in blue; **B**). *n* = 3 mice. Scale bar: 50 μm. (**C**) The cellular distribution of *Kcnn1* mRNA–positive neuronal somata in naive DRGs, small: 55%; medium: 39%; large: 6%. (**D**–**G**) Representative images of ISHH for *Kcnn1* mRNA (red) and immunohistochemistry for different DRG neuronal markers, calcitonin gene-related peptide (CGRP; green; **D**), isolectin B4 (IB4; green; **E**), tyrosine hydroxylase (TH; green; **F**), or neurofilament 200 (NF200; green; **G**), in the L3/4 DRGs. Arrows, double labeling neurons. *n* = 3 mice. Scale bar: 50 μm.

To investigate the role of DRG KCNN1 in neuropathic pain, we first examined KCNN1ʼs cellular distribution pattern in the DRG. Due to the lack of a commercially available KCNN1 antibody for immunohistochemical staining, we used in situ hybridization histochemistry (ISHH) assay to assess *Kcnn1* mRNA, followed by immunohistochemical staining of various DRG cell markers. *Kcnn1* mRNA was expressed in the cytoplasm and colocalized with β-tubulin III (a specific neuronal marker; Figure 1A), but not with glutamine synthetase (GS, a marker for satellite glial cells; Figure 1B), indicating that *Kcnn1* mRNA is expressed exclusively in DRG neurons. Approximately 49% (195 out of 397) of β-tubulin III–labeled neurons were positive for *Kcnn1* mRNA. A cross-sectional area analysis of neuronal somata revealed that *Kcnn1* mRNA was expressed in about 55% of small (<500 µm^2^ in area), 39% of medium (500–1,000 µm^2^ in area), and 6% of large (>1,000 µm^2^ in area) neurons (Figure 1C). Consistently, approximately 30% of *Kcnn1* mRNA–labeled neurons were positive for CGRP (Figure 1D), 39% for biotinylated IB4 (a marker for small nonpeptidergic neurons; Figure 1E), 33% for tyrosine hydroxylase (TH, a marker for small low-threshold neurons; Figure 1F), and 30% for neurofilament 200 (NF200, a marker for medium/large neurons and myelinated Aβ fibers; Figure 1G). These distribution patterns suggest *Kcnn1* mRNA’s involvement in the transmission and modulation of nociceptive information.

### Peripheral nerve injury downregulates Kcnn1 mRNA and KCNN1 protein in the injured DRG.

**Figure 2:**
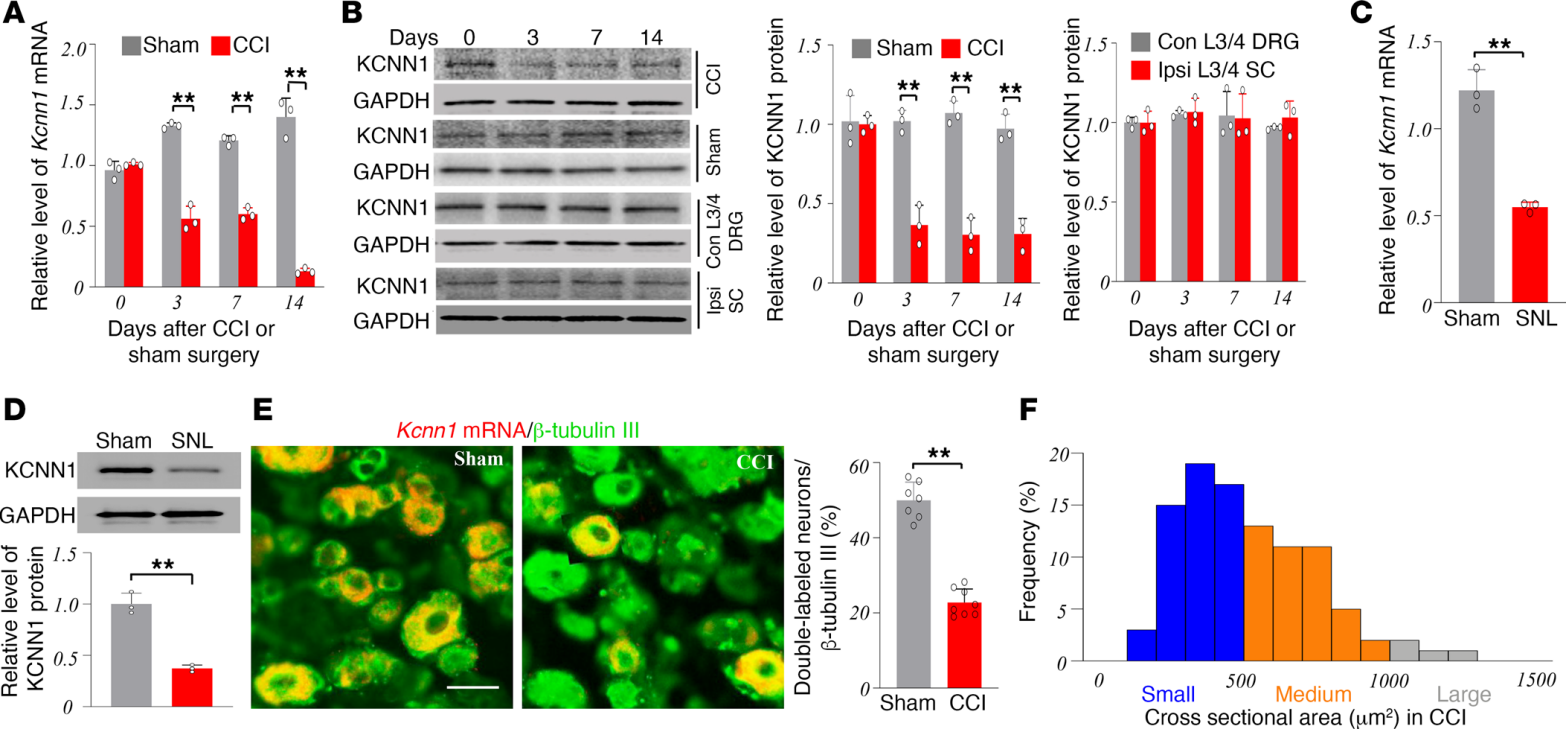
Peripheral nerve injury–induced downregulation of *Kcnn1* mRNA and KCNN1 protein in the injured DRG. (**A**) Expression of *Kcnn1* mRNA in the ipsilateral L3/4 DRGs on days 0, 3, 7, and 14 after CCI or sham surgery. *n* = 3 biological repeats (6 mice)/group/time point. **P < 0.01 by 2-way ANOVA followed by post hoc Tukey test. (**B**) Expression of KCNN1 protein in the ipsilateral L3/4 DRGs, contralateral (Con) L3/4 DRGs, and ipsilateral (Ipsi) L3/4 spinal cord (SC) on days 0, 3, 7, and 14 after CCI or sham surgery. *n* = 3 biological repeats (6 mice)/group/time point. **P < 0.01 by 2-way ANOVA followed by post hoc Tukey test. (**C** and **D**) Expression of *Kcnn1* mRNA (**C**) and KCNN1 protein (**D**) in the ipsilateral L4 DRG on day 7 after SNL or sham surgery. *n* = 3 biological repeats (12 mice)/group. **P < 0.01 by the 2-tailed unpaired Student’s *t* test. (**E**) Number of *Kcnn1* mRNA–labeled neurons in the ipsilateral L4 DRG on day 7 after CCI or sham surgery. Left: Representative images of ISHH for *Kcnn1* mRNA (red) and immunohistochemistry for β-tubulin III (green). Right: statistical summary. *n* = 3 mice/group. **P < 0.01 by the 2-tailed unpaired Student’s *t* test. Scale bar: 50 μm. (**F**) The cellular distribution of *Kcnn1* mRNA–positive neuronal somata in the ipsilateral L3/4 DRGs on day 7 after CCI. Small: 54%; medium: 42%; large: 4%.

We further examined whether the expression of *Kcnn1* mRNA and its encoded KCNN1 protein in the DRG was altered after peripheral nerve injury. Consistent with previous reports (19–22), CCI, but not sham surgery, induced mechanical allodynia, evidenced by marked increases in paw withdrawal frequencies in response to 0.07 g and 0.4 g von Frey filament stimuli on days 3, 7, and 14 after surgery on the ipsilateral (not contralateral) side (Supplemental Figure 1, A and B; supplemental material available online with this article; https://doi.org/10.1172/jci.insight.180085DS1). Additionally, heat and cold hyperalgesia, demonstrated by significant decreases in paw withdrawal latencies in response to heat and cold stimuli, respectively, was also observed on the ipsilateral (not contralateral) side at the same time points after CCI but not after sham surgery (Supplemental Figure 1, C and D). In line with the time-dependent behavioral changes, KCNN1 was downregulated in the ipsilateral L3/4 DRGs, but not in the contralateral L3/4 DRGs or the ipsilateral L3/4 spinal cord dorsal horn, after CCI (Figure 2, A and B). The levels of *Kcnn1* mRNA decreased by 58%, 50%, and 91% on days 3, 7, and 14 after CCI, respectively, compared with the corresponding days after sham surgery (Figure 2A). Consistently, KCNN1 protein levels in the ipsilateral L3/4 DRGs were significantly lower after CCI than after sham surgery on the same days (Figure 2B). Similar results were found following SNL (Figure 2, C and D). On day 7 after SNL, *Kcnn1* mRNA and KCNN1 protein decreased by 55% and 63%, respectively, in the ipsilateral L4 DRG, compared with the sham surgical group (Figure 2, C and D). Similarly, the number of *Kcnn1* mRNA–labeled neurons in the ipsilateral L3/4 DRGs on day 7 after CCI was reduced by 54% compared with the corresponding sham group (Figure 2E). However, CCI did not alter the distribution pattern of *Kcnn1* mRNA–labeled neurons in the ipsilateral L3/4 DRGs; approximately 54% of these neurons were small sized, 42% were medium sized, and 4% were large sized (Figure 2F).

### Rescuing downregulated KCNN1 in the injured DRG alleviates the development of CCI-induced nociceptive hypersensitivity.

**Figure 3:**
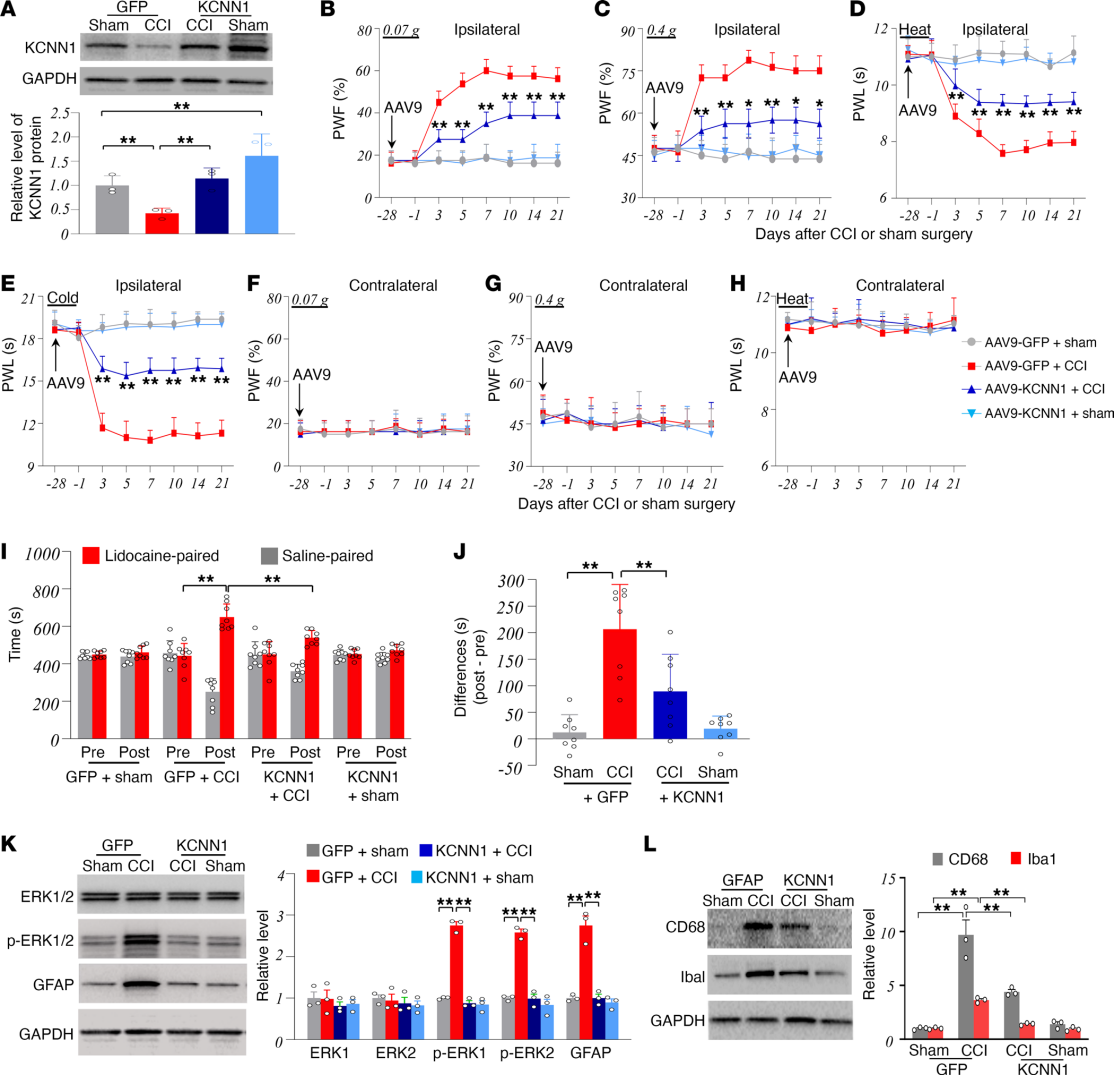
Effect of rescuing KCNN1 downregulation in the injured DRG on the development of CCI-induced nociceptive hypersensitivity in male mice. The mice were microinjected with AAV9-KCNN1 (KCNN1) or AAV5-GFP (GFP) into the ipsilateral L3/4 DRGs 28 days before CCI or sham surgery. (**A**) Expression of KCNN1 protein in the ipsilateral L3/4 DRGs on day 21 after CCI or sham surgery. *n* = 3 repeats (6 mice)/group. **P < 0.01 by 2-way ANOVA followed by post hoc Tukey test. (**B**–**H**) Paw withdrawal frequencies (PWF) in response to 0.07 g (**B** and **F**) and 0.4 g (**C** and **G**) von Frey filament stimuli and paw withdrawal latencies (PWL) to heat (**D** and **H**) and cold (**E**) stimuli on the ipsilateral (**B**–**E**) and contralateral (**F**–**H**) sides at the different days as indicated after CCI or sham surgery. *n* = 8 mice/group. *P < 0.05 or **P < 0.01 versus the AAV9-GFP*–*microinjected CCI group at the corresponding days by 3-way ANOVA with repeated measures followed by post hoc Tukey test. (**I** and **J**) Spontaneous nociceptive responses as assessed by the conditioned place preference (CPP) paradigm on day 21 after CCI or sham surgery. The time spent in each chamber (**I**) and difference scores for chamber preferences calculated by subtracting preconditioning (Pre) preference time from postconditioning (Post) time spent in the lidocaine-paired chamber (**J**). *n* = 8 mice/group. **P < 0.01 by 3-way ANOVA with repeated measures followed by post hoc Tukey test (**I**) or by 2-way ANOVA with repeated measures followed by post hoc Tukey test (**J**). (**K** and **L**) Expression of p-ERK1/2, total ERK1/2, GFAP (**K**), Iba1 (**L**), and CD68 (**L**) proteins in the ipsilateral L3/4 dorsal horn on day 21 after CCI or sham surgery. *n* = 3 repeats (6 mice)/group. **P < 0.01 by 2-way ANOVA followed by post hoc Tukey test.

Next, we investigated whether the downregulation of KCNN1 in the injured DRG contributed to the development of CCI-induced nociceptive hypersensitivity in male mice. To address this, we rescued KCNN1 expression in the injured DRG by microinjecting adeno-associated virus serotype 9 expressing full-length *Kcnn1* mRNA (AAV9-KCNN1) into the ipsilateral L3/4 DRGs 28 days before CCI or sham surgery. Mice microinjected with AAV9 expressing GFP (AAV9-GFP) served as controls. As anticipated, the level of KCNN1 protein in the ipsilateral L3/4 DRGs of AAV9-GFP–microinjected CCI mice was significantly reduced by 57% compared with AAV9-GFP–microinjected sham mice on day 21 after surgery (Figure 3A). This reduction was completely reversed in AAV9-KCNN1–microinjected CCI mice (Figure 3A). On day 21 after surgery, the amount of KCNN1 protein in the ipsilateral L3/4 DRGs of AAV9-KCNN1–microinjected sham mice increased by 1.6-fold compared with AAV9-GFP–microinjected sham mice (Figure 3A). DRG microinjection of AAV9-KCNN1 did not affect the CCI-induced reductions in the levels of K_2p_1.1 and Kv1.2 proteins in the ipsilateral L3/4 DRGs 21 days after surgery (Supplemental Figure 2).

As in the observation above, mechanical allodynia and heat and cold hyperalgesia were detected on the ipsilateral side from days 3 to 21 after CCI in the AAV9-GFP–microinjected male mice (Figure 3, B–E). DRG microinjection of AAV9-KCNN1 significantly attenuated CCI-induced increases in response to 0.07 g and 0.4 g von Frey filament stimuli (Figure 3, B and C), and CCI-induced reductions in response to heat and cold stimuli (Figure 3, D and E), from days 3 to 21 after CCI. Neither AAV9-KCNN1 nor AAV9-GFP microinjection into the DRGs affected basal paw withdrawal responses on the contralateral side of CCI mice or on either side of sham mice during the observation period (Figure 3, B–H). All microinjected mice maintained normal locomotor activities (Supplemental Table 1). Moreover, DRG microinjection of AAV9-KCNN1, but not AAV9-GFP, alleviated the spontaneous pain in male mice, as evidenced by a marked reduction in preference for the lidocaine-paired chamber on day 21 post-CCI (Figure 3, I and J). Sham male mice injected with either virus showed no clear preference for either chamber (Figure 3, I and J).

To further validate the behavioral observations above, we assessed the effect of DRG microinjection of AAV9-KCNN1 on CCI-induced neuronal, glial, and macrophage hyperactivities in the dorsal horn of male mice. These hyperactivities are triggered by DRG neuronal hyperexcitability-caused increase in the release of neurotransmitters/neuromodulators in primary afferents under neuropathic pain conditions (7). Consistent with previous reports (20, 22), levels of phosphorylated ERK1/2 (p-ERK1/2, a marker for neuronal hyperactivity), glial fibrillary acidic protein (GFAP, a marker for astrocyte hyperactivity), ionized calcium-binding adapter molecule 1 (Iba1, a marker for microglia), and CD68 (a marker for macrophages and monocytes), but not total ERK1/2, were significantly elevated in the ipsilateral L3/4 dorsal horn of AAV9-GFP–injected mice 21 days after CCI (Figure 3, K and L). These increases were not observed in the AAV9-KCNN1–microinjected CCI mice (Figure 3, K and L). Neither AAV9 altered basal levels of total ERK1/2, p-ERK1/2, GFAP, Iba1, and CD68 in the ipsilateral L3/4 dorsal horn of sham male mice (Figure 3, K and L). These findings indicate that rescuing KCNN1 expression in the injured DRG reduces CCI-induced central sensitization in the dorsal horn.

Similar results were observed in female CCI or sham mice with DRG microinjection of AAV9-KCNN1 or AAV9-GFP (Supplemental Figure 3, A–L).

### Rescuing downregulated KCNN1 in the injured DRG attenuates the maintenance of CCI-induced nociceptive hypersensitivity.

**Figure 4:**
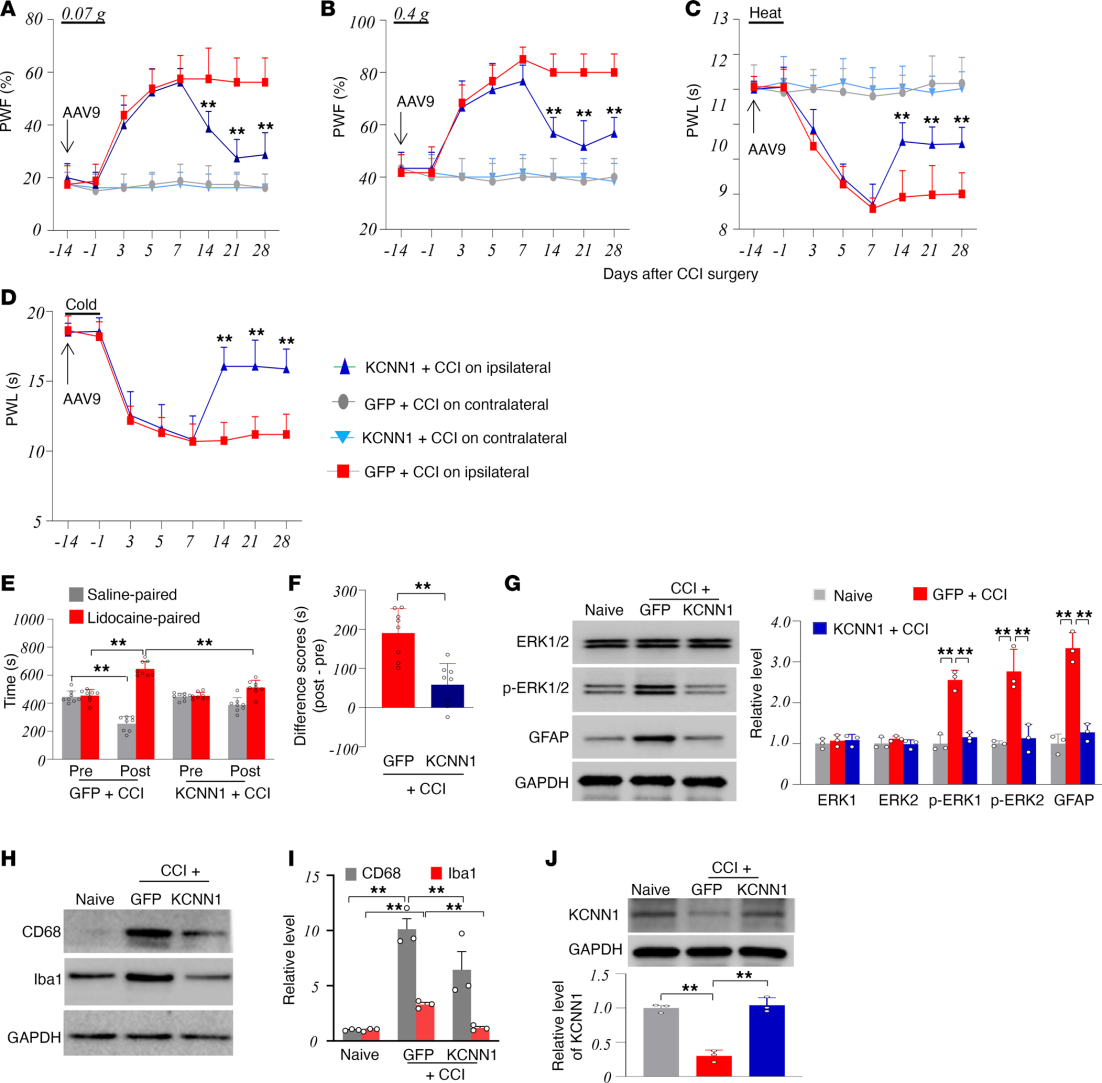
Effect of rescuing KCNN1 downregulation in the injured DRG on the maintenance of CCI-induced nociceptive hypersensitivity in male mice. The mice were microinjected with AAV9-KCNN1 or AAV9-GFP into the ipsilateral L3/4 DRGs 14 days before CCI. (**A**–**D**) Paw withdrawal frequencies (PWF) in response to 0.07 g (**A**) and 0.4 g (**B**) von Frey filament stimuli and paw withdrawal latencies (PWL) to heat (**C**) and cold (**D**) stimuli on the ipsilateral and contralateral sides at the different days as indicated after CCI. *n* = 8 mice/group. **P < 0.01 versus the AAV5-GFP*–*microinjected CCI group at the corresponding days on the ipsilateral side by 3-way ANOVA with repeated measures followed by post hoc Tukey test. (**E** and **F**) Spontaneous nociceptive responses as assessed by the conditioned place preference (CPP) paradigm on day 28 after CCI. The time spent in each chamber (**E**) and difference scores for chamber preferences calculated by subtracting preconditioning (Pre) preference time from postconditioning (Post) time spent in the lidocaine-paired chamber (**F**). *n* = 8 mice/group. **P < 0.01 by 2-way ANOVA with repeated measures followed by post hoc Tukey test (**E**) or by 2-tailed unpaired Student’s *t* test (**F**). (**G**–**I**) Expression of p-ERK1/2 (**G**), total ERK1/2 (**G**), GFAP (**G**), Iba1 (**H** and **I**), and CD68 (**H** and **I**) protein in the ipsilateral L3/4 dorsal horn on day 28 after CCI or from naive mice. *n* = 3 biological repeats (6 mice)/group. **P < 0.01 by 1-way ANOVA followed by post hoc Tukey test. (**J**) Expression of KCNN1 in the ipsilateral L3/4 DRGs on day 28 after CCI or from naive mice. *n* = 3 repeats (6 mice)/group. **P < 0.01 by 1-way ANOVA followed by post hoc Tukey test.

The role of KCNN1 downregulation in the injured DRG in the maintenance of the CCI-induced nociceptive hypersensitivity was also assessed. AAV9-GFP or AAV9-KCNN1 was microinjected into the ipsilateral L3/4 DRGs 14 days before CCI surgery, considering that AAV9 takes 3–4 weeks to express the target gene (23–27). CCI-induced mechanical allodynia and heat and cold hyperalgesia were fully developed on the ipsilateral side 7 days after CCI in both AAV9-GFP– and AAV9-KCNN1–microinjected groups (Figure 4, A–D). However, in the AAV9-KCNN1–microinjected group, these nociceptive hypersensitivities were alleviated on days 14, 21, and 28 after CCI (Figure 4, A–D). Basal paw withdrawal responses on the contralateral side remained unchanged in both groups of AAV9-microinjected CCI mice (Figure 4, A–D). On day 21 after CCI, the AAV9-KCNN1–microinjected mice showed reduced stimulation-independent spontaneous pain compared with the AAV9-GFP–microinjected CCI mice, as evidenced by a decreased preference for the lidocaine-paired chamber (Figure 4, E and F). Additionally, the increases in the amounts of p-ERK1/2 (not total ERK1/2), GFAP, Iba1, and CD68 in the ipsilateral L3/4 dorsal horn on day 28 after CCI from the AAV9-GFP–microinjected group were absent in the AAV9-KCNN1–microinjected group (Figure 4, G–I). As expected, the reduction in the level of KCNN1 in the ipsilateral L3/4 DRGs 28 days after CCI in the AAV9-GFP–microinjected mice was fully reversed in the AAV9-KCNN1–microinjected mice (Figure 4J). Taken together, these results suggest that the downregulation of KCNN1 in the injured DRG also contributes to the maintenance of CCI-induced nociceptive hypersensitivity and dorsal horn central sensitization.

### Mimicking CCI-induced DRG KCNN1 downregulation produces neuropathic pain-like symptoms.

**Figure 5:**
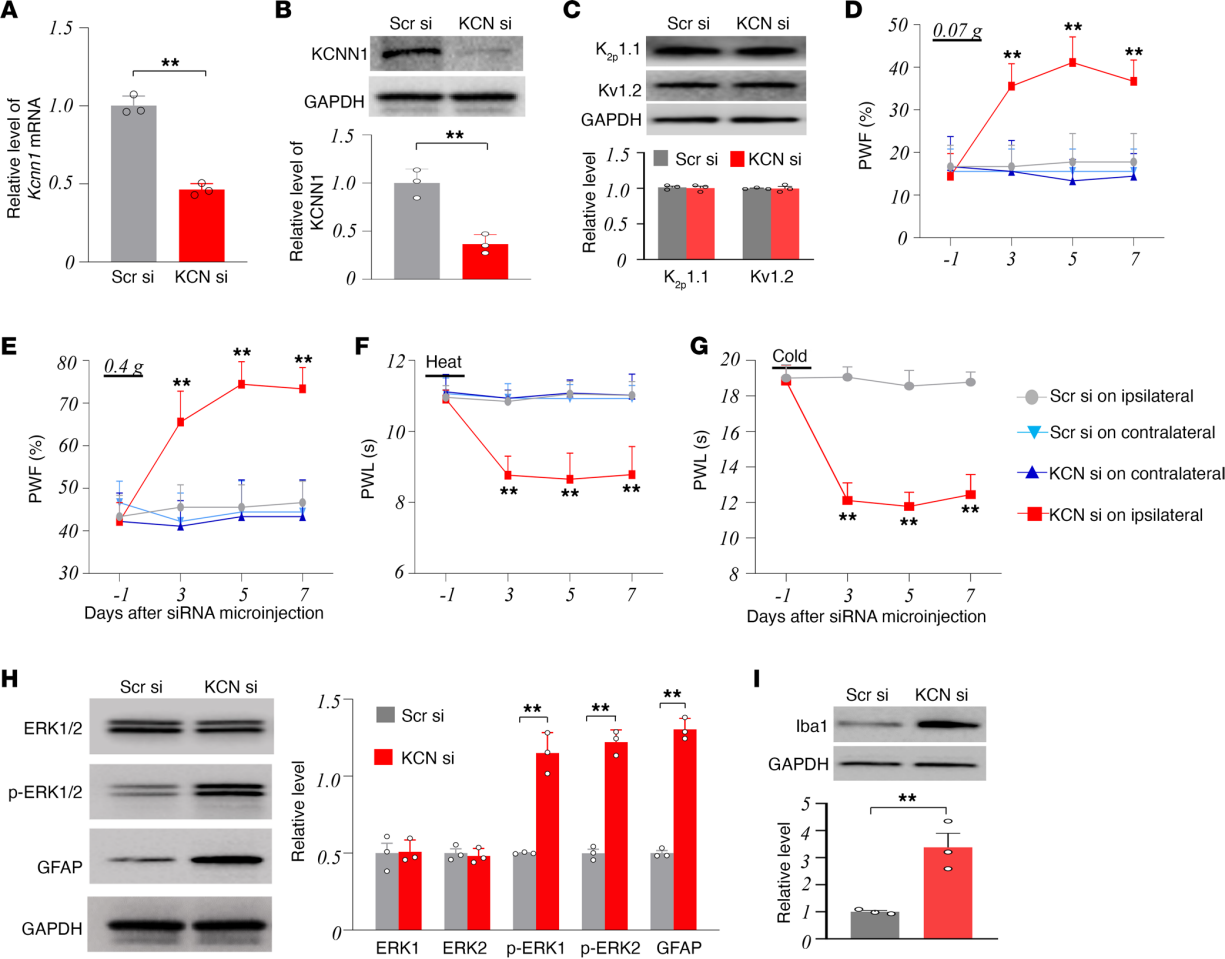
Effects of mimicking the CCI-induced DRG KCNN1 downregulation on basal nociceptive thresholds in naive male mice. (**A**–**C**) Expression of *Kcnn1* mRNA (**A**), KCNN1 protein (**B**), K_2p_1.1 protein (**C**), and Kv1.2 protein (**C**) in the ipsilateral L3/4 DRGs 7 days after microinjection of *Kcnn1* siRNA (KCN si) or control scrambled siRNA (Scr si) into unilateral L3/4 DRGs. *n* = 3 biological repeats (6 mice)/group. **P < 0.01 by 2-tailed unpaired Student’s *t* test. (**D**–**G**) Paw withdrawal frequencies (PWF) in response to 0.07 g (**D**) and 0.4 g (**E**) von Frey filament stimuli and paw withdrawal latencies (PWL) to heat (**F**) and cold (**G**) stimuli on the ipsilateral and contralateral sides on the days as indicated after microinjection of *Kcnn1* siRNA (KCN si) or control scrambled siRNA (Scr si) into unilateral L3/4 DRGs. *n* = 8 mice/group. **P < 0.01 versus the Scr si*–*microinjected group at the corresponding days on the ipsilateral side by 3-way ANOVA with repeated measures followed by post hoc Tukey test. (**H** and **I**) Expression of p-ERK1/2, total ERK1/2, GFAP (**H**), and Iba1 (**I**) in the ipsilateral L3/4 dorsal horn 7 days after microinjection of *Kcnn1* siRNA (KCN si) or control scrambled siRNA (Scr si) into unilateral L3/4 DRGs. *n* = 3 biological repeats (6 mice)/group. **P < 0.01 by 2-tailed unpaired Student’s *t* test.

To determine whether DRG downregulation of KCNN1 is sufficient for neuropathic pain, we knocked down DRG KCNN1 through microinjection of *Kcnn1*-specific small interfering RNA (*Kcnn1* siRNA) into the ipsilateral L3/4 DRGs of naive male mice, using scrambled siRNA as a control. As anticipated, levels of *Kcnn1* mRNA and KCNN1 protein decreased markedly by 54% and 63%, respectively, in the ipsilateral L3/4 DRGs of the *Kcnn1* siRNA–microinjected mice compared with the scrambled siRNA–microinjected mice on day 7 after microinjection (Figure 5, A and B). This microinjection did not alter basal levels of K_2p_1.1 and Kv1.2 in the ipsilateral L3/4 DRGs (Figure 5C), indicating the specificity and selectivity of *Kcnn1* siRNA. DRG microinjection of *Kcnn1* siRNA, but not the scrambled siRNA, produced marked increases in paw withdrawal frequencies to 0.07 g and 0.4 g von Frey filament stimuli and reductions in paw withdrawal latencies to heat and cold stimuli on the ipsilateral side on days 3, 5, and 7 after CCI (Figure 5, D–G). Neither siRNA altered basal responses on the contralateral side (Figure 5, D–G) or locomotor function (Supplemental Table 1). Additionally, DRG microinjection of *Kcnn1* siRNA, but not the scrambled siRNA, increased the amounts of p-ERK1/2 (not total ERK1/2), GFAP, and Iba1 in the ipsilateral L3/4 dorsal horn on day 7 after microinjection (Figure 5, H and I). CD68 level was undetectable or low in the ipsilateral L3/4 dorsal horn 7 days after microinjection of either siRNA (data not shown). Similar findings were observed in naive female mice injected with either *Kcnn1* siRNA or scrambled siRNA (Supplemental Figure 4, A–G). Collectively, our data suggest that, in the absence of nerve injury, downregulation of KCNN1 in the DRG leads to neuropathic pain-like symptoms.

### Rescuing downregulated KCNN1 in the injured DRG blocks the CCI-induced increase in DRG neuronal excitability.

**Figure 6:**
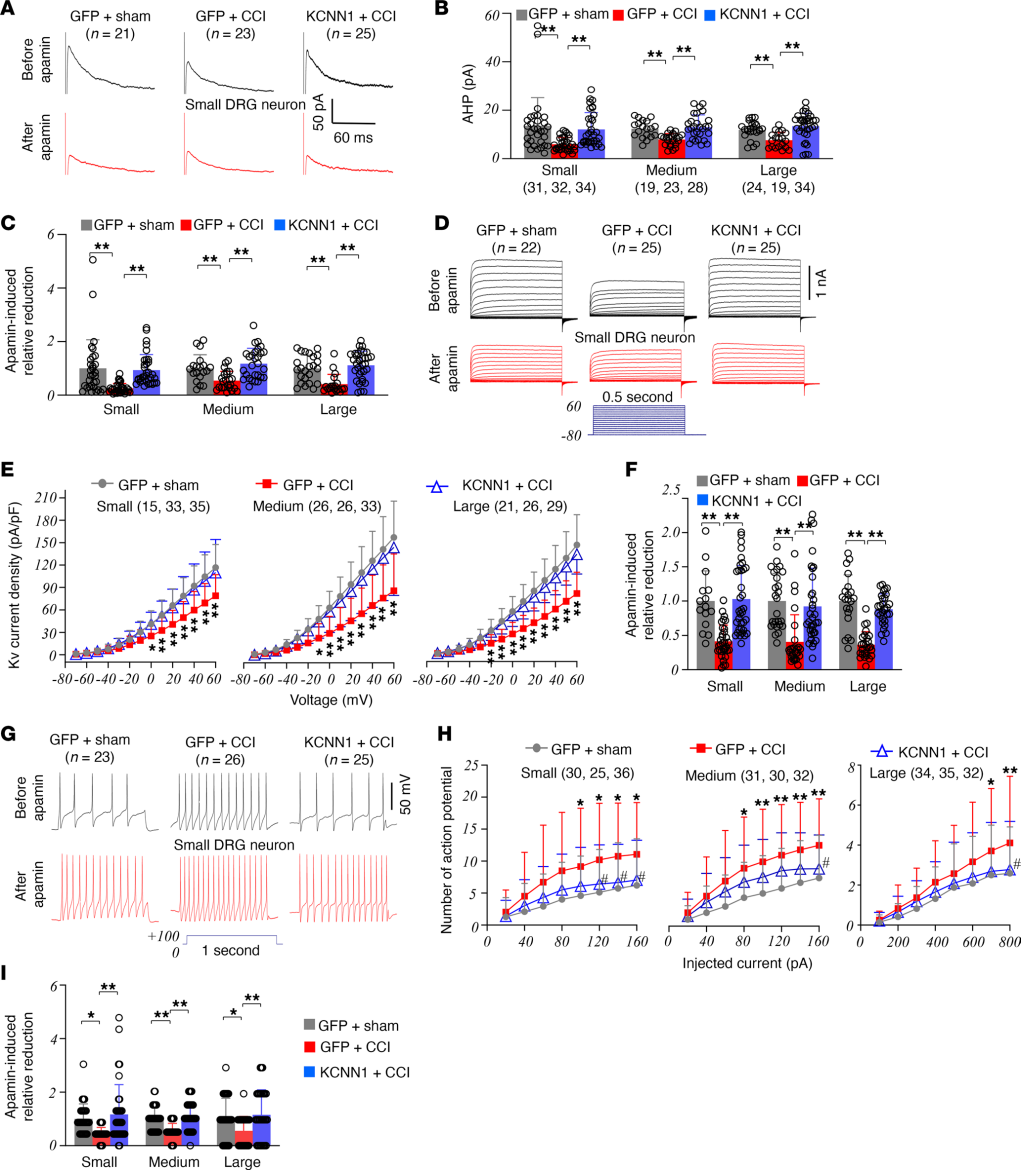
Downregulated KCNN1 participates in the CCI-induced increase of the excitability in injured DRG neurons. Recordings were conducted in the ipsilateral L3/4 DRGs 7–10 days after surgery in male mice with pre-microinjection of AAV-9-KCNN1/-GFP (KCNN1) or AAV9-GFP (GFP) into the injured DRG for 28 days. Number of the neurons recorded and number of mice used are indicated. (**A**) Representative traces of AHP currents. (**B** and **C**) Amplitudes of AHP currents before apamin application (**B**) and relative reductions in the amplitudes of AHP currents after apamin treatment (**C**) in small, medium, and large DRG neurons from 3 treated groups as indicated. **P < 0.01 by 2-way ANOVA with post hoc Tukey test. (**D**) Representative traces of total Kv current. (**E** and **F**) I–V curves before apamin application (**E**) and relative reductions in the amplitudes of total Kv currents (**F**) in small, medium, and large DRG neurons from 3 treated groups as indicated. *P < 0.05, **P < 0.01 between the GFP plus CCI mice and GFP plus sham mice or KCNN1 plus CCI mice at the corresponding voltage by 2-way ANOVA with repeated measures followed by post hoc Tukey test (**E**). **P < 0.01 by 2-way ANOVA with post hoc Tukey test (**F**). (**G**) Representative traces of action potentials. (**H** and **I**) The frequency of action potentials before apamin application (**H**) and relative increases in the frequency of action potentials after apamin treatment (**I**) in small, medium, and large DRG neurons from 3 treated groups as indicated. *P < 0.05, **P < 0.01 versus the GFP plus sham group at the corresponding voltage and ^#^*P* < 0.05 versus the GFP plus CCI group at the corresponding voltage by 3-way ANOVA with repeated measures followed by post hoc Tukey test (**H**). *P < 0.05, **P < 0.01 by 2-way ANOVA with post hoc Tukey test (**I**).

KCNN1 mediates AHP and neuronal excitability (9). We first investigated the effects of restoring KCNN1 levels in the injured DRG on CCI-induced decreases in AHP and total Kv currents in DRG neurons. To increase recording efficiency, we microinjected either AAV9-GFP or a mixture of AAV9-KCNN1 and AAV9-GFP (AAV9-KCNN1/-GFP) into the ipsilateral L3/4 DRGs 28 days before CCI or sham surgery. Whole-cell patch-clamp recordings were performed on acutely disassociated GFP-labeled DRG neurons from the ipsilateral L3/4 DRGs 7–10 days after CCI or sham surgery. As anticipated, AHP currents and total Kv current densities were significantly lower in small, medium, and large DRG neurons from AAV9-GFP–microinjected CCI mice compared with AAV9-GFP sham mice (Figure 6, A–F). These decreases were fully reversed in the AAV9-KCNN1/-GFP–microinjected CCI mice (Figure 6, A–F). Bath application of 100 nM apamin (a selective KCNN blocker, ref. 17) reduced AHP and Kv currents. These reductions were less profound in all 3 types of DRG neurons from AAV9-GFP–microinjected CCI mice compared with those from AAV9-GFP–microinjected sham mice or AAV9-KCNN1/-GFP–microinjected CCI mice (Figure 6, A–F). These findings strongly indicate that the decreases in AHP currents and total Kv current densities observed in small, medium, and large DRG neurons from AAV9-GFP–microinjected CCI mice are likely attributed to KCNN1 downregulation.

Furthermore, we examined the impact of rescuing KCNN1 downregulation on the CCI-induced increase in excitability of injured DRG neurons. As predicted, the numbers of action potentials evoked by ≥100 pA stimulation in small neurons, ≥80 pA in medium DRG neurons, and ≥700 pA in large DRG neurons (1 second) were significantly higher in AAV9-GFP–microinjected CCI mice, compared with AAV9-GFP–microinjected sham mice (Figure 6, G and H). These increases were not observed in AAV9-KCNN1/-GFP–microinjected mice (Figure 6, G and H). Bath application of 100 nM apamin resulted in a smaller increase in the number of action potentials in all 3 types of DRG neurons from AAV9-GFP–microinjected CCI mice compared with AAV9-GFP–microinjected sham mice or AAV9-KCNN1/-GFP–microinjected CCI mice (Figure 6I). While the numbers of spontaneous action potentials significantly increased in small, medium, and large DRG neurons from AAV9-GFP–microinjected CCI mice (Supplemental Figure 5A), this increase was absent in small and large DRG neurons or tended to be reduced in medium DRG neurons from the AAV9-KCNN1/-GFP–microinjected mice (Supplemental Figure 5A). Additionally, the CCI-induced decreases in resting membrane potentials in small, medium, and large DRG neurons from AAV9-GFP–microinjected CCI mice were not observed in AAV9-KCNN1/-GFP–microinjected mice (Supplemental Figure 5B), whereas the CCI-induced decreases in rheobases in small, medium, and large DRG neurons from AAV9-GFP–microinjected CCI mice were markedly blocked only in small DRG neurons from the AAV9-KCNN1/-GFP–microinjected mice (Supplemental Figure 5C). No significant changes were observed in membrane input resistances or other action potential parameters, such as amplitudes, overshoots, and thresholds, across the microinjected groups (Supplemental Table 2).

### Reduced ESRRG participates in the CCI-induced KCNN1 downregulation in the injured DRG.

**Figure 7:**
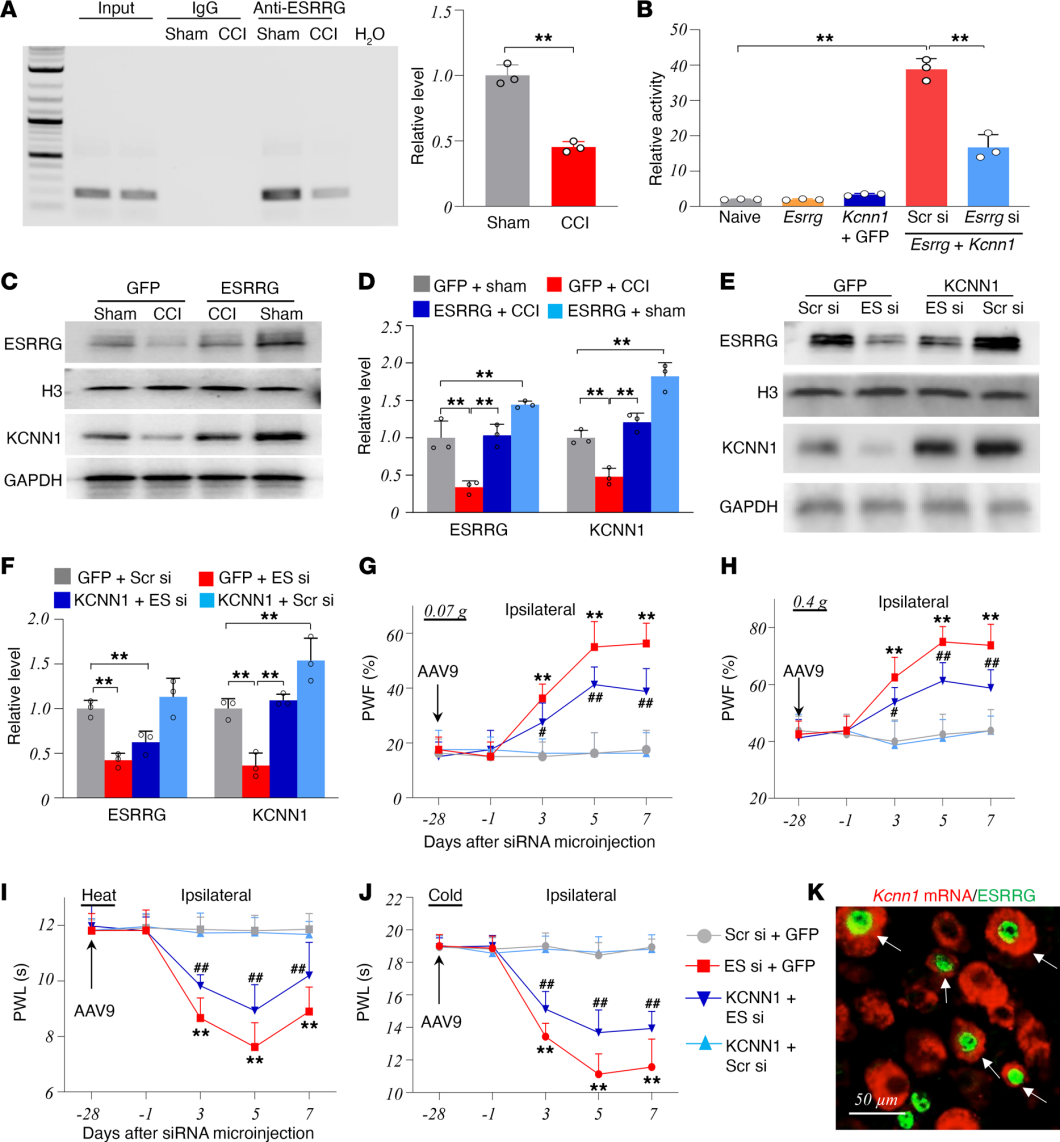
ESRRG reduction is responsible for the CCI-induced downregulation of KCNN1 in the injured DRG. (**A**) The binding activity of ESRRG to the *Kcnn1* gene promoter in the ipsilateral L3/4 DRGs 7 days postsurgery. *n* = 15 mice/group. **P < 0.01 by 2-tailed unpaired Student’s *t* test. (**B**) *Kcnn1* promoter activity in the CAD cells treated with the vectors/siRNAs as shown. *n* = 3 repeats/group. **P < 0.01 by 1-way ANOVA followed by Tukey post hoc test. Naive, pGL3-Basic; *Kcnn1*, pGL3-*Kcnn1* report vector; *Esrrg* si, *Esrrg* siRNA; Scr si, scrambled siRNA; *Esrrg*, vector expressing *Esrrg* mRNA; GFP, vector expressing GFP. (**C** and **D**) Expression of ESRRG and KCNN1 in the ipsilateral L3/4 DRGs 21 days after surgery in mice with pre-microinjection of AAV5-ESRRG (ESRRG) or AAV5-GFP (GFP). *n* = 6 mice/group. **P < 0.01 by 2-way ANOVA followed by post hoc Tukey test. (**E** and **F**) Expression of ESRRG and KCNN1 in the ipsilateral L3/4 DRGs 7 days after microinjection of *Esrrg* siRNA (ES si) or scrambled siRNA (Scr si) in mice with pre-microinjection of AAV9-KCNN1 (KCNN1) or AAV9-GFP (GFP). *n* = 6 mice/group. **P < 0.01 by 1-way ANOVA followed by post hoc Tukey test. (**G**–**J**) Paw withdrawal frequencies (PWF; **G** and **H**) and paw withdrawal latencies (PWL; **I** and **J**) on the days as indicated after microinjection of *Esrrg* siRNA (ES si) or scrambled siRNA (Scr si) in mice with pre-microinjection of AAV9-KCNN1 (KCNN1) or AAV9-GFP (GFP). *n* = 8 mice/group. **P < 0.01 versus the GFP plus Scr si group or ^#^*P* < 0.05, ^##^*P* < 0.01 versus the GFP plus ES si group at the corresponding days by 2-way ANOVA with repeated measures followed by post hoc Tukey test. (**K**) Coexpression of *Kcnn1* mRNA with ESRRG (arrows) in naive L4 DRG neurons. *n* = 3 mice.

Finally, we explored how peripheral nerve injury caused KCNN1 downregulation in the injured DRG. Given that transcription factors regulate gene expression, we used online Jaspar software (http://tfbsdb.systemsbiology.net/) and identified a consensus binding motif (_–1,175_TGAAGGTGAT_–1,184_) for the transcription factor estrogen-related receptor gamma (ESRRG) within the *Kcnn1* gene promoter. Chromatin immunoprecipitation (ChIP) assay revealed that a *Kcnn1* promoter fragment containing this binding motif could be amplified from the complex immunoprecipitated with ESRRG antibody in nuclear fractions from the DRG of sham mice (Figure 7A). This amplification did not occur with control normal serum (Figure 7A), suggesting specific ESRRG binding to the *Kcnn1* promoter in the DRG. CCI significantly reduced the binding of ESRRG to the *Kcnn1* promoter, as evidenced by a 55% decrease in binding density in the ipsilateral L3/4 DRGs on day 7 after CCI, compared with the sham group (Figure 7A). Furthermore, the luciferase assay revealed that cotransfection of the full-length *Esrrg* vector (as opposed to the GFP vector) with the *Kcnn1* reporter vector plus control scrambled siRNA significantly increased the *Kcnn1* gene promoter activity in in vitro CAD cells (Figure 7B). This increase was notably diminished in CAD cells cotransfected with the full-length *Esrrg* vector and *Kcnn1* reporter vector along with *Esrrg* siRNA (Figure 7B), indicating that ESRRG is specifically responsible for activating *Kcnn1* promoter. In in vivo experiments, male mice that received AAV9-GFP microinjection into the ipsilateral L3/4 DRGs 28 days before CCI exhibited a significant reduction in ESRRG level in the ipsilateral L3/4 DRGs on day 7 following CCI (Figure 7, C and D). Rescuing this reduction through microinjection of AAV9 expressing full-length *Esrrg* mRNA (AAV9-ESRRG) into the ipsilateral L3/4 DRGs of male mice 28 days before CCI reversed the CCI-induced downregulation of KCNN1 in the ipsilateral L3/4 DRGs on day 7 after CCI (Figure 7, C and D). This microinjection also alleviated CCI-induced mechanical allodynia and heat/cold hyperalgesia on the ipsilateral (not contralateral) side from days 3 to 21 after CCI, as well as spontaneous ongoing pain on day 21 after CCI (Supplemental Figure 6, A–I). Additionally, it blocked the CCI-induced increases in the amounts of p-ERK1/2 (but not total ERK1/2), GFAP, Iba1, and CD68 in the ipsilateral L3/4 dorsal horn 21 days after CCI (Supplemental Figure 6, A–I). As expected, microinjection of AAV9-ESRRG into the ipsilateral L3/4 DRGs of sham male mice led to increased basal levels of ESRRG and KCNN1 in these microinjected DRGs (Figure 7, C and D). Moreover, mimicking the CCI-induced reduction of DRG ESRRG through microinjection of the specific *Esrrg* siRNA into the unilateral L3/4 DRGs of naive mice 28 days after microinjecting AAV9-GFP into these DRGs led to a decease in the levels of ESRRG and KCNN1 in the ipsilateral L3/4 DRGs on day 7 after siRNA microinjection (Figure 7, E and F). This intervention also heightened sensitivity to mechanical, heat, and cold stimuli on the ipsilateral (but not contralateral) side from days 3 to 7 after microinjection (Figure 7, G–J, and Supplemental Figure 7, A–C) and increased basal amounts of p-ERK1/2 (not total ERK1/2), GFAP, and Iba1 in the ipsilateral L3/4 dorsal horn 7 days after microinjection (Supplemental Figure 7, D and E). These changes were not observed in naive male mice microinjected with AAV9-KCNN1 plus *Esrrg* siRNA (Figure 7, E–J, and Supplemental Figure 7, D and E). Double-labeled immunofluorescence staining revealed that about 68% of *Kcnn1* mRNA–labeled neurons were positive for ESRRG in the DRG neurons (Figure 7K). These findings suggest that the CCI-induced downregulation of KCNN1 is attributed, at least in part, to the reduction of ESRRG in injured DRG neurons.

## Discussion

Peripheral nerve injury caused by CCI or SNL leads to robust and long-lasting nociceptive hypersensitivities in preclinical mouse models of neuropathic pain. These include stimulation-evoked mechanical allodynia, heat/cold hyperalgesia, and stimulation-independent spontaneous ongoing pain. These models mirror the clinical symptoms seen in patients with neuropathic pain following breast surgery, cardiac surgery, thoracotomy, or limb amputation (1, 2). Elucidating how nociceptive hypersensitivities are caused by peripheral nerve injury may provide new avenues for the management of neuropathic pain. The present study demonstrated the downregulation of KCNN1 in the injured DRGs after CCI or SNL. This downregulation was necessary to develop and maintain CCI-induced nociceptive hypersensitivity, as it decreased action potential AHP and total Kv currents while increasing excitability in injured DRG neurons. Our findings suggest that KCNN1 could be a promising potential target for therapeutic management of neuropathic pain.

Similar to other Kv channels, such as Kv1.2 (28) and K_2p_1.1 (29), KCNN1 is exclusively present in DRG neurons and can be regulated following peripheral nerve injury. An early study using a specific KCNN1 antibody reported that KCNN1 protein was expressed mainly in small and medium DRG neurons (less than 1,000 μm^2^), with some expression in large DRG neurons (greater than 1,000 μm^2^), in rats (13). Approximately 87% of KCNN1-positive DRG neurons were labeled by IB4, and 56% of them were positive for CGRP (13). In the present study, the ISHH assay revealed that around 94% of DRG *Kcnn1* mRNA–labeled neurons were smaller than 1,000 μm^2^ in soma size. Additionally, we found that approximately 30% of *Kcnn1* mRNA–positive neurons were labeled with CGRP and 39% of them with IB4 in naive mouse DRGs. The reasons for the differences in proportions of these 2 markers between the present study and previous work (13) are unclear. It is yet to be determined whether these discrepancies result from the ISHH staining process affecting CGRP antigen integrity or IB4 binding. Interestingly, about 30% of *Kcnn1* mRNA–positive neurons were labeled by NF200, suggesting that *Kcnn1* mRNA is expressed in different subtypes of DRG neurons. Indeed, approximately 33% of *Kcnn1* mRNA–labeled DRG neurons were positive for TH. The diverse distribution of *Kcnn1* mRNA in DRG neurons likely indicates its functional heterogeneity.

Importantly, our present and previous studies demonstrated that CCI or SNL led to the downregulation of *Kcnn1* mRNA and KCNN1 protein in the injured DRG (25). Both models complement each other, as CCI-induced nociceptive hypersensitivity mainly results from ischemia, while SNL-induced nociceptive hypersensitivity is initiated primarily by nerve injury (30–32). This downregulation likely occurs in all subtypes of DRG neurons, as the distribution pattern of *Kcnn1* mRNA expression in small, medium, and large neurons of the ipsilateral L3/4 DRGs was unchanged on day 7 after CCI compared with naive DRGs, despite a marked reduction in total number of *Kcnn1* mRNA–labeled neurons in these injured DRGs. Our findings strongly support the previous observations that KCNN1 expression was reduced in the DRGs avulsed from the patients with neuropathic pain (18) and that apamin-sensitive Kv currents were decreased in the injured DRG neurons after SNL (17). However, Mongan et al. reported no alternation of KCNN1 expression in injured DRGs 15 days after CCI in rats (13). The reasons for the inconsistent results between the present work and the previous study are still being investigated. Since this previous study did not report behavioral results (13), the lack of changes in KCNN1 expression in the injured DRG may be attributed to an unsuccessful CCI model. Given the time-dependent correlation between DRG KCNN1 downregulation and the induction and maintenance of CCI-induced nociceptive hypersensitivity, our data suggest that downregulated KCNN1 in the DRGs may play a role in neuropathic pain.

ESRRG, an orphan nuclear hormone receptor that belongs to the estrogen-related receptor subfamily of transcription factors (33, 34), is involved in transcriptional silencing of Kcnn1 gene expression in injured DRGs. It regulates the transcription of genes involved in various endocrine and metabolic signals and plays significant roles in pathological conditions such as insulin resistance, alcoholic liver injury, and cardiac hypertrophy (33, 34). The present study demonstrated that CCI reduced the expression of ESRRG in the injured DRG. This reduction likely leads to the nerve injury–induced transcriptional silencing of *Kcnn1* gene in the injured DRG neurons. ESRRG directly bound to and activated the promoter region of *Kcnn1* gene. CCI decreased the binding of ESRRG to the *Kcnn1* gene promoter in the injured DRG. Moreover, rescuing the reduction of ESRRG in the DRG attenuated the development of CCI-induced nociceptive hypersensitivity and reversed the CCI-induced downregulation of KCNN1 in the injured DRG. Mimicking the CCI-induced reduction of DRG ESRRG downregulated basal KCNN1 expression in the DRG and produced enhanced behavioral responses to noxious stimuli in naive mice. Rescuing KCNN1 downregulation in the DRG markedly mitigated these enhanced behavioral responses. Our findings suggest that KCNN1 is a downstream target of ESRRG, mediating its role in neuropathic pain.

It should be noted that other mechanisms may also contribute to the downregulation of *Kcnn1* mRNA in the injured DRG. For instance, our previous study reported that the downregulation of sensory neuron–specific long noncoding RNA (*SS-lncRNA*) decreased the recruitment of hnRNPM to the *Kcnn1* promoter, leading to the silencing of *Kcnn1* in the injured DRG (25). hnRNPM is an mRNA-binding heterogeneous nuclear ribonucleoprotein (35). How it contributes to *SS-lncRNA* downregulation–induced reduction of *Kcnn1* promoter activity in the injured DRG remains unclear. In the present study, we found that ESRRG directly bound to the *Kcnn1* promoter and activated it, but the mechanism by which ESRRG is recruited to the *Kcnn1* promoter is not yet known. Whether reduced ESRRG is involved in *SS-lncRNA* downregulation–induced reduction of *Kcnn1* promoter activity, possibly due to a decrease in the binding of hnRNPM to ESRRG and the consequent recruitment of less ESRRG to the *Kcnn1* promoter, remains to be determined in our future study. Additionally, the involvement of other transcription factors, epigenetic modifications, or a decrease in *Kcnn1* mRNA stability in the injured DRG could not be ruled out and will be investigated in the future.

Downregulation of DRG KCNN1 is necessary for peripheral nerve injury–induced nociceptive hypersensitivity. KCNN1 contributes to total Kv currents (25) and determines AHP, which helps regulate neuronal excitability (9). Consistent with previous reports (36–38), CCI reduced the AHP and total Kv currents and increased the numbers of evoked and spontaneous action potentials in small, medium, and large neurons of the ipsilateral L3/4 DRGs. Given that KCNN1 is expressed in small, medium, and large DRG neurons and that CCI downregulated its expression in all 3 types of DRG neurons, rescuing the downregulation of DRG KCNN1 blocked CCI-induced changes. Although there was a tendency toward reversing the CCI-induced increase in spontaneous action potentials in medium neurons of the ipsilateral L3/4 DRGs, this change was not significant. Bath application of apamin had a lesser effect on the CCI-induced decrease of AHP currents and total Kv currents, as well as on the increase in the number of evoked action potentials, in AAV9-GFP–microinjected CCI mice compared with AAV9-KCNN1/-GFP–microinjected CCI mice or AAV9-GFP–microinjected sham mice. These findings further emphasize the role of DRG KCNN1 downregulation in the electrophysiological changes induced by CCI in injured DRG neurons. KCNN channels have been shown to contribute to the resting membrane potential and conductance (39). Our current-clamp recording revealed that reversing DRG KCNN1 downregulation markedly counteracted the CCI-induced reductions in resting membrane potentials in small, medium, and large DRG neurons, as well as in rheobase in small DRG neurons. This effect aligns with a previous study showing that KCNN1 overexpression could substantially hyperpolarize the resting membrane potential in the transfected HEK293 cells (40). Why rescuing DRG KCNN1 downregulation did not significantly reverse the CCI-induced decrease in rheobase in medium and large DRG neurons is not yet clear and requires further investigation. Behavioral observations from current and prior studies indicated that reversing DRG KCNN1 downregulation attenuated the development and maintenance of nociceptive hypersensitivity in mice subjected to CCI or SNL (25). Furthermore, mimicking this downregulation produced neuropathic pain-like symptoms in naive mice (25). DRG KCNN1 downregulation is likely critical for the onset and maintenance of neuropathic pain by increasing DRG neuronal excitability. Neuronal excitability triggers the release of transmitters and neuromodulators in primary afferents, contributing to central sensitization in the dorsal horn. Indeed, the observed inhibitory effect of reversing DRG KCNN1 downregulation on CCI-induced hyperactivity in dorsal horn neurons, astrocytes, microglia, and macrophages strongly supports this hypothesis.

In summary, this study demonstrated the role of ESRRG-controlled KCNN1 downregulation in injured DRGs in neuropathic pain. Alleviation of neuropathic pain was achieved by rescuing this downregulation through the microinjection of AAV9-KCNN1 in the DRGs, without affecting locomotor activity and acute/basal pain. Given that AAV has been used as a delivery vehicle in the COVID-19 vaccine, the local delivery of exogenous KCNN1 via AAV into the DRGs may hold potential as a novel antinociceptive treatment for managing neuropathic pain.

## Methods

### Sex as a biological variable.

The present study examined male and female mice, and similar findings were reported for both sexes.

### Animal preparations.

CD1 mice (approximately 7–8 weeks, male or female, weighing 20–25 g) were obtained from Charles River Laboratories. The animals were housed in a facility room at 23 ± 2°C under an automatic 12-hour light/12-hour dark cycle and provided with food and water ad libitum. To minimize interindividual variability in behavioral outcome measurements, the animals were acclimatized for at least 2 days before the experiments. All experiments were carried out by investigators with group assignment and treatment conditions blinded. These efforts aimed to minimize the suffering of animals and reduce the number of animals used.

### Neuropathic pain models.

The preclinical mouse model of CCI-induced neuropathic pain was conducted as previously described (19–22). In brief, mice were anesthetized with 2% isoflurane. The unilateral sciatic nerve was exposed and loosely ligated with 6–0 catgut at 3 sites, with an interval of about 1 mm proximal to the trifurcation of the sciatic nerve. Sham mice underwent the same surgery but without ligation.

The preclinical mouse model of SNL-induced neuropathic pain was also performed as described previously (41–44). Briefly, after the mice were anesthetized with 2% isoflurane, the unilateral L4 transverse process was identified and removed. The underlying L4 spinal nerve was carefully isolated, ligated with a 6–0 catgut, and then transected just distal to the ligature. Sham mice underwent an identical surgery but without transection and ligation of the L4 spinal nerve.

### DRG microinjection.

DRG microinjection was performed following the protocols established in our previous studies (41–44). Briefly, after the mice were anesthetized as described above, a midline incision was made in the lower lumbar back region. The unilateral L3/4 articular processes were removed, exposing the corresponding DRGs. AAV9 (1 μL/DRG, 4 × 10^12^ to 5 × 10^12^ genome copies/mL) or siRNA (1 μL/DRG; 40–80 μM) was microinjected into the exposed DRGs for 5–10 minutes using a glass micropipette connected to a Hamilton syringe under dissection microscopy. The siRNA was dissolved in TurboFect in vivo transfection reagent (catalog 00954573; Thermo Fisher Scientific) to improve delivery and prevent degradation of siRNA (45). After microinjection, the glass micropipette was kept for 10 minutes in the same position. Finally, the surgical area was irrigated with sterile saline, and the skin incision was closed with wound clips. Mice displaying abnormal locomotor activities were excluded from the experiment.

### Behavioral tests.

The evoked pain tests, including mechanical, heat, and cold assessments, were conducted in order at 1-hour intervals before viral microinjection or surgery and at various points after surgery, following the previously described protocols (41–44). The CPP test was conducted on day 14 or 21 after surgery, and the locomotor functional test was performed before tissue collection.

Paw withdrawal frequencies in response to mechanical stimuli were measured using 2 calibrated von Frey filaments (0.07 and 0.4 g, Stoelting Co.). Briefly, mice were individually placed in Plexiglas chambers on an elevated mesh screen. Each von Frey filament was applied to the plantar side of the hind paw for approximately 1 second and repeated 10 times at 5-minute intervals. A quick paw withdrawal was considered a positive response. The number of positive responses within the 10 trials was recorded as the percentage withdrawal: (number of paw withdrawals/10 trials) × 100 = % response frequency.

Paw withdrawal latencies in response to noxious heat stimuli were examined. Mice were individually placed in Plexiglas chambers on a glass plate. The heat stimuli were delivered using a Model 336 Analgesic Meter (IITC Life Science Inc.). A beam of light was emitted from a hole in the light box and applied to the middle of the plantar surface of each hind paw. A quick lift of the hind paw was considered a positive response, prompting the light to be turned off. The time between the start and the stop of the light beam was defined as the paw withdrawal latency. Each paw was tested 3 times with 5-minute intervals between trials. A cutoff time of 20 seconds was set to prevent damage to the hind paw.

Paw withdrawal latencies in response to cold stimuli were examined using a cold aluminum plate (–1~0°C). The temperature was continuously monitored by a thermometer set at –1~0°C. Each mouse was placed in a Plexiglas chamber on a cold aluminum plate. The time between placing the mouse on the plate and the onset of jumping was defined as the paw withdrawal latency, specifically on the ipsilateral side. Each trial was repeated 2 times at 30-minute intervals. A cutoff time of 20 seconds was applied in each trial to prevent potential injury to the mouse.

The CPP test was conducted following our previous studies with minor modifications (41–44). Briefly, an apparatus with 2 Plexiglas chambers connected by an internal door (Med Associates Inc.) was used. One chamber had a rough floor and walls with black and white horizontal stripes, while the another had a smooth floor and walls with black and white vertical stripes. The movement of the mice and the time spent in each chamber were monitored by photo beam detectors installed along the chamber walls and automatically recorded in MED-PC IV CPP software. The mouse was first preconditioned for 30 minutes to habituate the environment with full access to 2 chambers. At the end of preconditioning, the basal time duration spent in each chamber was recorded within 15 minutes to assess any preexisting chamber bias. Mice spending more than 80% or less than 20% of the total time in either chamber were excluded from further testing. The conditioning protocol took place over the next 3 days with the internal door between the chambers closed. In the morning, the mouse first received an intrathecal injection of saline (5 μL) specifically paired with 1 conditioning chamber for 15 minutes. Six hour later, lidocaine (0.8% in 5 μL saline) was administered intrathecally and paired with the opposite conditioning chamber for 15 minutes in the afternoon. The order of saline and lidocaine injections was alternated each day. On the test day, the mouse was placed in the chamber with the door open, allowing free access to both chambers. The movement and duration of each mouse spent in each chamber were recorded for 15 minutes to assess chamber preference. Difference scores were calculated as the difference between the time spent in the lidocaine chamber during the test and preconditioning.

Locomotor function tests, including placing, grasping, and righting reflexes, were performed as described previously (41–44). For the placing reflex, the hind limbs were positioned slightly lower than the forelimbs, and the dorsal surfaces of the hind paws were brought into contact with the edge of a table. It was then recorded whether the hind paws were reflexively placed on the table surface. For the grasping reflex, animals were placed on a wire grid to observe if the hind paws would grasp the wire. Regarding the righting reflex, animals were placed on their back on a flat surface to observe if they could assume the normal upright position. Each trial was repeated 5 times at a 5-minute interval, and the score for each test was recorded based on the numbers of times the normal reflex was observed.

### Western blotting assay.

Protein extraction and Western blotting analysis were conducted according to our previously published protocol (41–44). Briefly, unilateral L3/4 DRGs from 2 mice were pooled to obtain sufficient protein. L3/4 DRG or ipsilateral L3/4 spinal cord samples were homogenized on ice with lysis buffer. After centrifugation at 4°C for 15 minutes at 1,000*g*, the pellet (nuclear fraction) and the supernatant (membrane/cytosolic fractions) fractions were collected. The pellet was dissolved in a lysis buffer containing 2% sodium dodecyl sulfate and 0.1% Triton X-100. The samples (20 μg/sample) were heated at 99°C for 5 minutes, then loaded onto a 4% stacking/7.5% separating sodium dodecyl sulfate-polyacrylamide gel (catalog 345-0032; Bio-Rad Laboratories) and electrophoretically transferred onto a polyvinylidene difluoride membrane (catalog AM10100; Bio-Rad Laboratories). The membranes were incubated first in the blocking buffer (5% nonfat milk plus 0.1% Tween 20 in the Tris-buffered saline) for 2 hours at room temperature and then in the following primary antibodies at 4°C overnight. The primary antibodies include goat anti-KCNN1 (1:1,000, catalog LS-C803980; Lifespan biosciences, Absolute Biotech), rabbit anti–p-p44/42 MAPK (p-ERK1/2) (1:1,000, catalog 4370S; Cell Signaling Technology), rabbit anti-p44/42 MAPK (ERK1/2) (1:1,000, catalog 4695S; Cell Signaling Technology), mouse anti-GFAP (1:1,000, catalog 3670S; Cell Signaling Technology), rabbit anti-GAPDH (1:1,000, catalog G9545; Santa Cruz Biotechnology), mouse anti-ESRRG (1:500, catalog sc-376449; Santa Cruz Biotechnology), mouse anti-Iba1 (1:2,000, catalog SAB2702364; MilliporeSigma), rat anti-CD68 (1:1,000, catalog 600-401-R10; Rockland), or mouse anti–histone H3 (1:1,000, catalog sc-56616; Santa Cruz Biotechnology). After the membranes were washed with phosphate-buffered saline (PBS) containing 0.1% Tween 20, the proteins were detected using the HRP-conjugated donkey anti-goat (catalog number: 705-005-003), goat anti-rabbit (catalog number: 111-005-003), or goat anti-mouse (catalog number: 115-005-003) secondary antibody (1:4,000, Jackson ImmunoResearch) at room temperature for 1 hour. Visualization was achieved using the Western peroxide reagent, and luminol/enhancer reagent (Clarity Western ECL Substrate, Bio-Rad Laboratories), and exposure was conducted using the ChemiDoc XRS System with Image Lab software (Bio-Rad). The intensity of blots was quantified with densitometry using Image Lab software. All cytosol protein bands were normalized to GAPDH, while all nucleus protein bands were normalized to total histone H3.

### Quantitative real-time reverse transcription polymerase chain reaction assay.

Total RNA extraction and quantitative real-time reverse transcription polymerase chain reaction (RT-PCR) assay were conducted according to our previously published protocols (23–27). Briefly, unilateral L3/4 DRGs from 2 CCI or sham mice, unilateral L4 DRG from 4 SNL or sham mice, or the cultured DRG neurons from 1 well of a 6-well plate were rapidly collected and pooled to obtain sufficient RNA. Total RNA was extracted using TRIzol-chloroform methods (Invitrogen, Thermo Fisher Scientific), treated with an overdose of deoxyribonuclease I (New England Biolabs), and reverse-transcribed with the ThermoScript Reverse Transcriptase (Invitrogen, Thermo Fisher Scientific) and oligo(dT) primers (Invitrogen, Thermo Fisher Scientific) or specific RT primers. A 4 μL template was amplified in a Bio-Rad Laboratories CFX96 real-time PCR system using the primers (Supplemental Table 3). Each sample was run in triplicate in a 20 μL reaction volume containing 200 nM forward and reverse primers, 10 μL of SsoAdvanced Universal SYBR Green Supermix (Bio-Rad Laboratories), and 20 ng of cDNA. The PCR cycling parameters included a 3-minute incubation at 95°C, 39 cycles of 95° for 30 seconds, and 60° for 30 seconds, 72°C for 30 seconds, and 5 minutes at 72°C. All PCR data were normalized to a corresponding internal control *Tuba1*α. The ratios of mRNA levels at different points after surgery to mRNA levels at day 0 were calculated using the ΔCt method (2^−ΔΔCt^).

### ISHH.

The ISHH was performed as described previously with minor modifications (24, 27, 45). Briefly, after mice were deeply anesthetized with isoflurane, they were transcardially perfused with 50–100 mL of 4% paraformaldehyde in 0.1 M DEPC-treated phosphate buffer (pH 7.4). Bilateral L3/4 DRGs were collected, postfixed with the same perfused solution for 2–3 hours, and cryoprotected in 30% sucrose in 1× DEPC-treated PBS at 4°C for 2 nights. The tissues were sectioned at a thickness of 15 μm on a cryostat. The sections were initially incubated in 25 μg/mL proteinase K (catalog 3115828001; Roche) at 37°C for 10 minutes. After being washed with 1× DEPC-treated PBS, the sections were prehybridized (ISH Kit; BioChain Institute Inc) for 1.5 hours at 62°C. The *Kcnn1* complementary RNA probe (0.6 kb) was prepared by in vitro transcription (primers in Supplemental Table 3) and labeled with digoxigenin-deoxyuridine triphosphate (dUTP) according to the manufacturer’s instructions (Roche Diagnostics). The sections were hybridized with a digoxigenin-dUTP–labeled probe (2 ng/μL) at 62°C overnight. After being washed with 2× SSC for 15 minutes at 62°C twice, 1.5× SSC for 15 minutes at 62°C twice, and 0.2× SSC for 20 minutes at 62°C twice, the sections were incubated with 1× blocking solution for 2 hours at room temperature. Subsequently, they were incubated with alkaline phosphatase–conjugated anti-digoxigenin antibody (1:300, catalog number: 11093274910; Roche) and chicken anti–β-tubulin III (1:1,000, catalog number: AB9354; MilliporeSigma), mouse anti-ESRRG (1:50, Santa Cruz Biotechnology), mouse anti-NF200 (1:100, catalog number: N4142; MilliporeSigma), biotinylated IB4 (1:200, catalog number L2140; MilliporeSigma), mouse anti-CGRP (1:50, catalog number: ab81887; Abcam), mouse anti-TH (1:200, catalog number: sc-25269; Santa Cruz Biotechnology), or mouse anti-GS (1:800, catalog number: MAB302; MilliporeSigma) overnight at 4°C. The fluorescent signals were developed with Fast Red (catalog number: F4648; MilliporeSigma) and species-appropriate fluorescence-conjugated secondary antibody (Alexa Fluor 488 Donkey Anti-Chicken, code: 703-545-155; Cy2 Goat Anti-Mouse, code: 115-226-071; Jackson ImmunoResearch) or FITC-labeled avidin (catalog number: A2901, MilliporeSigma). Images were captured using a Leica DMI4000 fluorescence microscope with a DFC365 FX camera (Leica). The number of the neurons (with nucleus) double-labeled by *Kcnn1* mRNA and each marker as well as the number of the neurons (with nucleus) single-labeled by β-tubulin III or *Kcnn1* mRNA in each section were counted. At least 7–12 DRG sections from 3 mice per group were examined. Average percentages of double-labeled neurons were calculated by dividing the number of double-labeled neurons by the total number of single-labeled neurons.

### Plasmid constructs and virus production.

Full-length *Esrrg* cDNA and full-length *Kcnn1* cDNA were amplified from mouse DRG RNA using the SuperScript III One-Step RT-PCR system with the Platinum Taq High Fidelity Kit (Thermo Fisher Scientific) and the primers in Supplemental Table 3. All PCR products were ligated into the BglII and BsrBI restriction sites of the pAAV-CMV-MS vector (Cell Biolabs) to replace the enhanced GFP sequence (Supplemental Figure 8, A and B). The sequences of recombinant clones were verified by DNA sequencing. To produce viral particles, the recombinant viral vectors and packaging vectors were cotransfected into HEK293T cells (Thermo Fisher Scientific). AAV particles were harvested and purified using AAVpro Purification Kit (Takara). The titer was determined using the AAV real-time PCR titration kit (Takara).

### Luciferase assay.

The 1,953 bp fragment from the mouse *Kcnn1* promoter region (including ESRRG-binding motifs) was amplified by PCR using the primers listed in Supplemental Table 3 to construct the *Kcnn1* reporter vector (Supplemental Figure 8C). The CAD cells (MilliporeSigma) were plated on a 12-well plate and cultured at 37°C in a humidified incubator with 5% CO_2_. One day after culturing, the cells in each well were cotransfected with 300 ng of a vector expressing full-length *Esrrg* mRNA, 300 ng of a pGL3-Basic vector with or without the *Kcnn1* promoter reporter vector, and 10 ng of the pRL-TK (Promega) using Lipofectamine 3000 (Invitrogen, Thermo Fisher Scientific), according to the manufacturer’s instructions. Two days after transfection, the cells were collected and lysed in a passive lysis buffer. Approximately 10 μL of the supernatant was used to measure the luciferase activity using the Dual-Luciferase Reporter Assay System (Promega). Independent transfection experiments were repeated 3 times. The relative reporter activity was calculated after the normalization of the firefly activity to renilla activity.

### ChIP assay.

The ChIP assays were conducted using the EZ ChIP Kit (catalog MAGNA0002; Upstate, MilliporeSigma) as described (23–27). The homogenate from the DRG was cross-linked with 1% formaldehyde at room temperature for 10 minutes. The reaction was stopped by the addition of glycine (1×). After centrifugation at 800*g*, the pellet was collected and lysed in sodium dodecylsulfate lysis buffer containing a protease inhibitor cocktail (catalog J64963.LQ; Thermo Fisher Scientific). The lysis was sonicated until the DNA was sheared into fragments with a mean length of 200 to 1,000 nt. The samples were first precleaned with protein G magnetic beads (CS200638, MilliporeSigma) and then subjected to immunoprecipitation overnight with 2.5 µg of anti-ESRRG antibody (PA5-95510, Thermo Fisher Scientific) or 2.5 µg of normal rabbit purified IgG (ab171870, Abcam) overnight at 4°C. The input (10% of the sample for immunoprecipitation) was used as a positive control. After purification, the DNA fragments were amplified using the PCR/real-time PCR assay with the primers listed in Supplemental Table 3.

### Whole-cell patch-clamp recording.

The preparation of acutely disassociated DRG neurons for whole-cell patch-clamp recording was performed as described previously (23–27). To increase recording efficiency, mice received microinjection of AAV9-GFP or a mixed viral solution of AAV9-GFP plus AAV9 expressing full-length *Kcnn1* mRNA (AAV9-KCNN1) into the unilateral L3/4 DRGs 28 days before CCI or sham surgery. Only GFP-labeled (green) neurons were recorded 7 days after CCI or sham surgery. The acutely dissociated neurons from the ipsilateral L3/4 DRGs were collected and plated on laminin-coated coverslips. Whole-cell patch-clamp recordings were conducted at the temperature of 36°C 4 to 10 hours after plating. Total Kv currents were determined using a method described in our previous studies (26, 27, 46). In brief, for Kv current isolation, the bath solution contained the following reagents (in mM): choline-Cl 130, KCl 5, CdCl_2_ 1, CaCl_2_ 2, MgCl_2_ 1, HEPES 10, glucose 10 (pH = 7.4 with Tris-base, 320 mOsm). The intracellular pipette solution contained (in mM): potassium gluconate 120, KCl 20, MgCl_2_ 2, EGTA 10, HEPES 10, Mg-ATP 4 (pH = 7.3 with KOH, 310 mOsm). The Kv currents were recorded under the voltage model with 500 ms depolarizing voltage steps (command voltages to potentials ranging between –80 and +60 mV in 10 mV increments, holding at –80 mV). The resting membrane potential was measured 3 minutes after obtaining a stable recording. Action potentials (APs) were recorded under the current model. The rheobase current was defined as the first step current that induced 1 AP by 50 ms depolarizing step current injections. APs were evoked using a 1-second depolarizing current pulse (80 pA, 1.5 times the average rheobase current). Small and medium DRG neurons were given increasingly depolarizing current steps at +20 pA intervals ranging from 0 pA to +160 pA, while large neurons were at +100 pA intervals ranging from +100 pA to +800 pA. This allowed us to measure AP generation, in response to membrane depolarization (1-second duration). The AP amplitude was measured between the peak and the baseline. The AP overshoot was measured between the AP peak and 0 mV. Input resistance was measured during a 50 pA step (1-second duration) and calculated by dividing the steady-state voltage response by the current-pulse amplitude (–50 pA), presented as MOhms (MΩ). AHP currents were recorded under the voltage-clamp model with 500 ms depolarizing pulses (from –60 to 0 mV, holding at –60 mV). The AHP amplitude was measured between the maximum hyperpolarization and the final plateau voltage. The artificial cerebrospinal fluid consisted of the following (in mM): NaCl 140, KCl 5, CaCl_2_ 2, MgCl_2_ 1, HEPES 10, and glucose 10, with pH adjusted to 7.3 by NaOH. Micropipette resistance ranged from 4 to 6 MΩ. The patch pipette solution contained the following (in mM): KCl 135, MgATP 3, Na_2_ATP 0.5, CaCl_2_ 1.1, EGTA 2, and glucose 5; pH was adjusted to 7.38 with KOH and osmolarity adjusted to 300 mOsm with sucrose. Recording signals were amplified with an Axopatch 700B (Molecular Devices), filtered at 2 kHz, and sampled at 5 kHz using pCLAMP 10.7 software (Molecular Devices).

### Statistics.

Animals were randomly assigned to various treatment groups. All data were expressed as mean ± SD. The number of animals and sample sizes used in each study were determined based on our previous studies (26, 27, 46), pilot work, and power analysis (power of 0.09 at *P* < 0.05) and are consistent with those reported previously in the neuropathic pain research field. Statistical analysis was performed using GraphPad Prism 8.0.1. The data were statistically analyzed using a 2-tailed independent Student’s *t* test and a 1-way, 2-way, or 3-way ANOVA, as appropriate. When ANOVA showed a significant difference, a pairwise comparison between means was performed using the post hoc Tukey method. Significance was set at *P* < 0.05.

### Study approval.

The experimental procedures received the approval of the Animal Care and Use Committee of the New Jersey Medical School at Rutgers University, in accordance with the ethical guidelines of the NIH and the International Association for the Study of Pain.

### Data availability.

The raw data for all graphs are reported in the Supporting Data Values file.

## Author contributions

YXT supervised all experiments and conceived the project. HW, WZ and YXT designed the project. HW, XH, YL and XL performed the animal modeling; conducted behavioral experiments; and carried out microinjection, Western blot, in situ hybridization, immunohistochemistry, and PCR experiments. WZ conducted electrophysiological recording. XF did ChIP and luciferase assays. DS and BW helped with ISHH. HW, WZ, XF, BY, JHY, HH, and YXT analyzed the data. HW and YXT wrote the manuscript. HW, WZ, XF, XH, YL, BW, DS, XL, BY, JHY, HH, and YXT read and edited the manuscript.

## Supplementary Material

Supplemental data

Unedited blot and gel images

Supporting data values

## Version 1 06/24/2024

Electronic publication
