# Supplementary material for: ESRRG-controlled downregulation of KCNN1 in primary sensory neurons is required for neuropathic pain
Source: JCI Insight. 2024 May 21;9(12):e180085. doi: 10.1172/jci.insight.180085 (PMC11383585; doi:10.1172/jci.insight.180085)
Supplement: Supplemental data [file jciinsight-9-180085-s088.pdf]

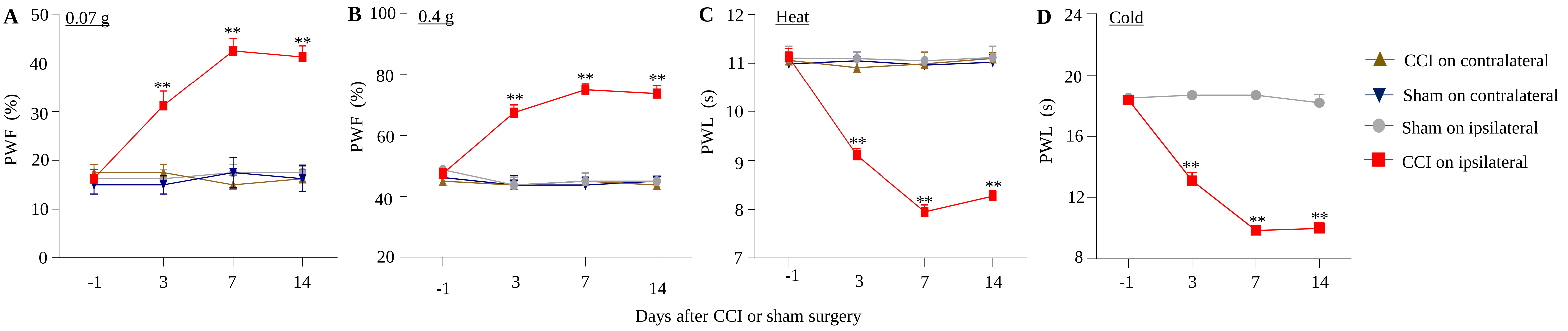

Supplementary Figure 1. CCI led to the development of nociceptive hypersensitivity. (A-D) Paw withdrawal frequencies (PWF) in response to 0.07 g (A) and 0.4 g (B) von Frey filament stimuli and paw withdrawal latencies (PWL) to heat (C) and cold (D) stimuli on the ipsilateral and contralateral sides at the different days as indicated after CCI or sham surgery. n = 8 mice/group. $^{**}P < 0.01$ versus the sham group at the corresponding days by two-way ANOVA with repeated measures followed by post hoc Tukey test.

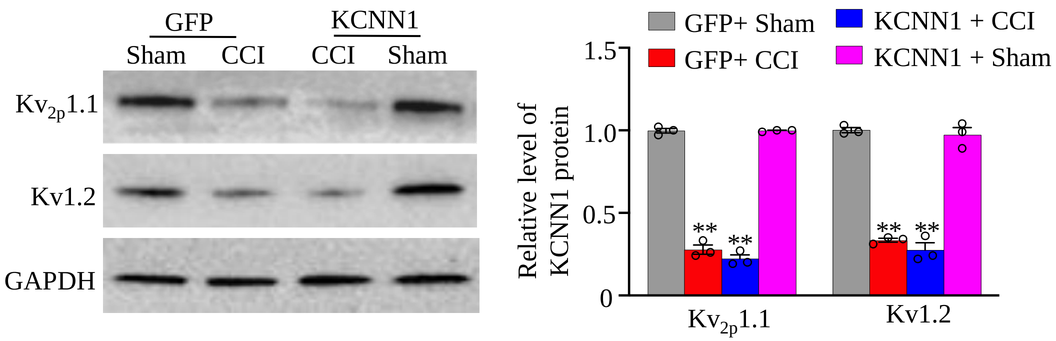

Supplementary Figure 2. Expression of $K_{2p}1.1$ and Kv1.2 proteins in the ipsilateral L3/4 DRGs on day 21 after CCI or sham surgery in the mice with microinjection with AAV9-KCNN1 (KCNN1) or AAV5-GFP (GFP) into the ipsilateral L3/4 DRGs 28 days before CCI or sham surgery. n = 3 repeats (6 mice)/group. ***P* < 0.01 by two-way ANOVA followed by post hoc Tukey test.

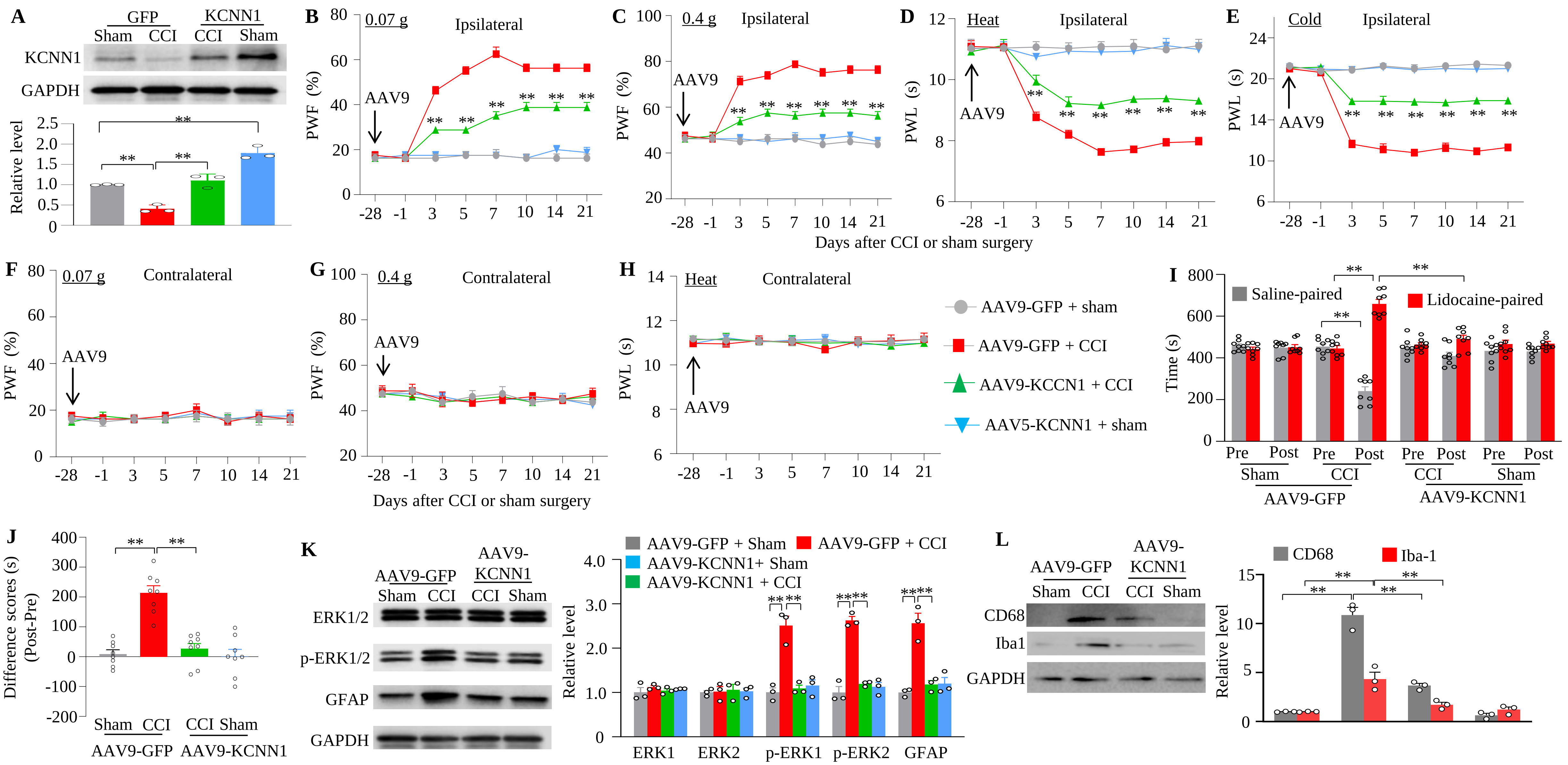

A
GFP
KCNN1
Sham CCI CCI Sham
KCNN1
GAPDH
Relative level
B
0.07 g
Ipsilateral
PWF (%)
AAV9
C
0.4 g
Ipsilateral
D
Heat
Ipsilateral
PWL (s)
E
Cold
Ipsilateral
Days after CCI or sham surgery
F
0.07 g
Contralateral
G
0.4 g
Contralateral
H
Heat
Contralateral
AAV9-GFP + sham
AAV9-GFP + CCI
AAV9-KCCN1 + CCI
AAV5-KCNN1 + sham
I
Saline-paired
Lidocaine-paired
Time (s)
Pre Post Pre Post Pre Post Pre Post
Sham CCI CCI Sham
AAV9-GFP
AAV9-KCNN1
J
Difference scores (s) (Post-Pre)
Sham CCI CCI Sham
AAV9-GFP AAV9-KCNN1
K
AAV9-GFP
AAV9-KCNN1
Sham CCI CCI Sham
ERK1/2
p-ERK1/2
GFAP
GAPDH
AAV9-GFP + Sham
AAV9-GFP + CCI
AAV9-KCNN1+ Sham
AAV9-KCNN1 + CCI
Relative level
ERK1 ERK2 p-ERK1 p-ERK2 GFAP
L
AAV9-GFP
AAV9-KCNN1
Sham CCI CCI Sham
CD68
Iba1
GAPDH
CD68
Iba-1
Relative level

Supplementary Figure 3. Effect of rescuing KCNN1 downregulation in injured DRG on the development of CCI-induced nociceptive hypersensitivity in female male mice. The mice were microinjected with AAV9-KCNN1 (KCNN1) or AAV5-GFP (GFP) into the ipsilateral L3/4 DRGs 28 days before CCI or sham surgery. (A) Expression of KCNN1 protein in the ipsilateral L3/4 DRGs on day 21 after CCI or sham surgery. n = 3 repeats (6 mice)/group. ** $P < 0.01$ by two-way ANOVA followed by post hoc Tukey test. (B-H) Paw withdrawal frequencies (PWF) in response to 0.07 g (B and F) and 0.4 g (C and G) von Frey filament stimuli and paw withdrawal latencies (PWL) to heat (D and H) and cold (E) stimuli on the ipsilateral (B-E) and contralateral (F-H) sides at the different days as indicated after CCI or sham surgery. n = 8 mice/group. * $P < 0.05$ or ** $P < 0.01$ versus the AAV9-GFP-microinjected CCI group at the corresponding days by three-way ANOVA with repeated measures followed by post hoc Tukey test. (I and J) Spontaneous nociceptive responses as assessed by the CPP paradigm on day 21 post-CCI or sham surgery. Time spent in each chamber (I) and difference scores for chamber preferences calculated by subtracting preconditioning (Pre) preference time from postconditioning (Post) time spent in the lidocaine-paired chamber (J). n = 8 mice/group. ** $P < 0.01$ by three-way ANOVA with repeated measures followed by post hoc Tukey test (I) or by two-way ANOVA with repeated measures followed by post hoc Tukey test (J). (K, L) Expression of p-ERK1/2 (K), total ERK1/2 (K), GFAP (K), Iba1 (L), and CD68 (L) proteins in the ipsilateral L3/4 dorsal horn on day 21 after CCI or sham surgery. n = 3 repeats (6 mice)/group. ** $P < 0.01$ by two-way ANOVA followed by post hoc Tukey test.

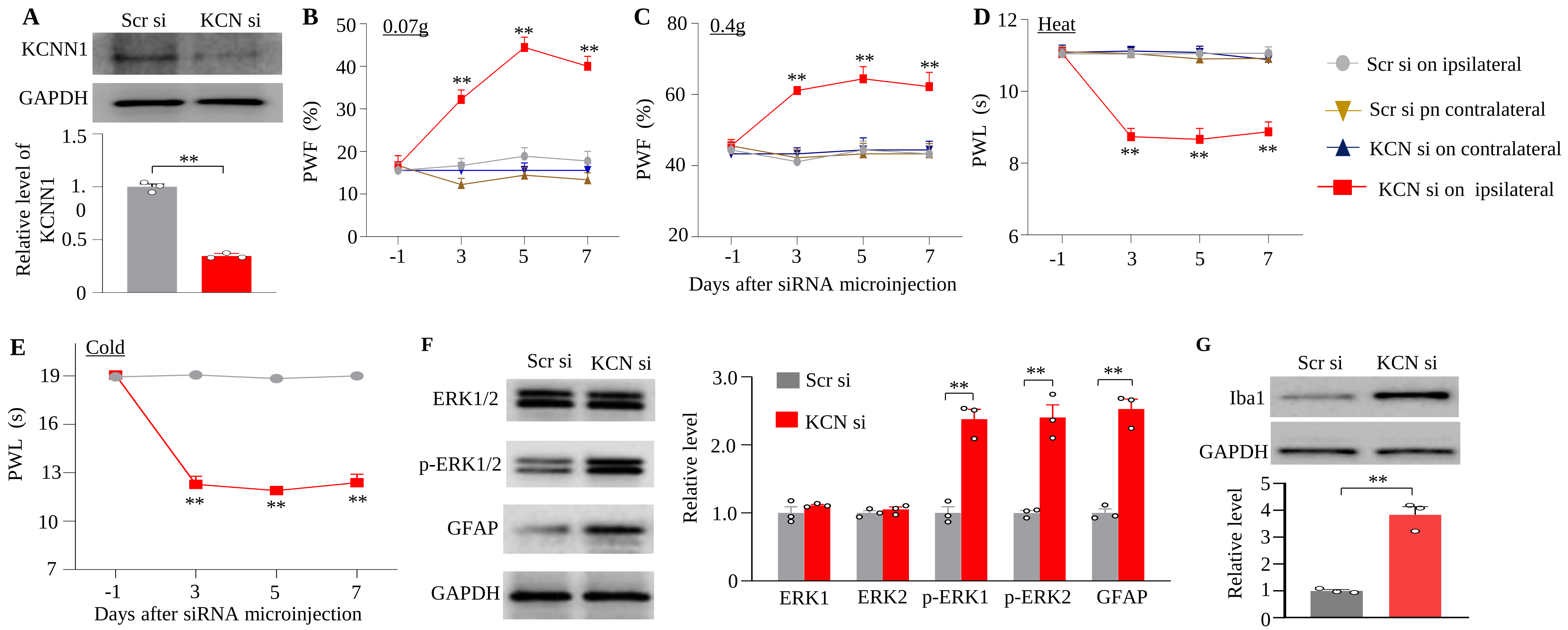

Supplementary Figure 4. Effects of mimicking the CCI-induced DRG KCNN1 downregulation on basal nociceptive thresholds in naïve female mice. (A) Expression of KCNN1 protein (A) in the ipsilateral L3/4 DRGs 7 days after microinjection of *Kcnn1* siRNA (KCN si) or control scrambled siRNA (Scr si) into unilateral L3/4 DRGs. n = 3 biological repeats (6 mice)/group. $^{**}P < 0.01$ by two-tailed unpaired Student *t* test. (B-E) Paw withdrawal frequencies (PWF) in response to 0.07 g (B) and 0.4 g (C) von Frey filament stimuli and paw withdrawal latencies (PWL) to heat (D) and cold (E) stimuli on the ipsilateral and contralateral sides at the days as indicated after microinjection of *Kcnn1* siRNA (KCN si) or control scrambled siRNA (Scr si) into unilateral L3/4 DRGs. n = 8 mice/group. $^{**}P < 0.01$ versus the Scr si-microinjected group at the corresponding days on the ipsilateral side by three-way ANOVA with repeated measures followed by post hoc Tukey test. (F, G) Expression of p-ERK1/2 (F), total ERK1/2 (F), GFAP (F), and Iba1 (G) in the ipsilateral L3/4 dorsal horn 7 days after microinjection of microinjection of *Kcnn1* siRNA (KCN si) or control scrambled siRNA (Scr si) into unilateral L3/4 DRGs. n = 3 biological repeats (6 mice)/group. $^{**}P < 0.01$ by two-tailed unpaired Student *t* test.

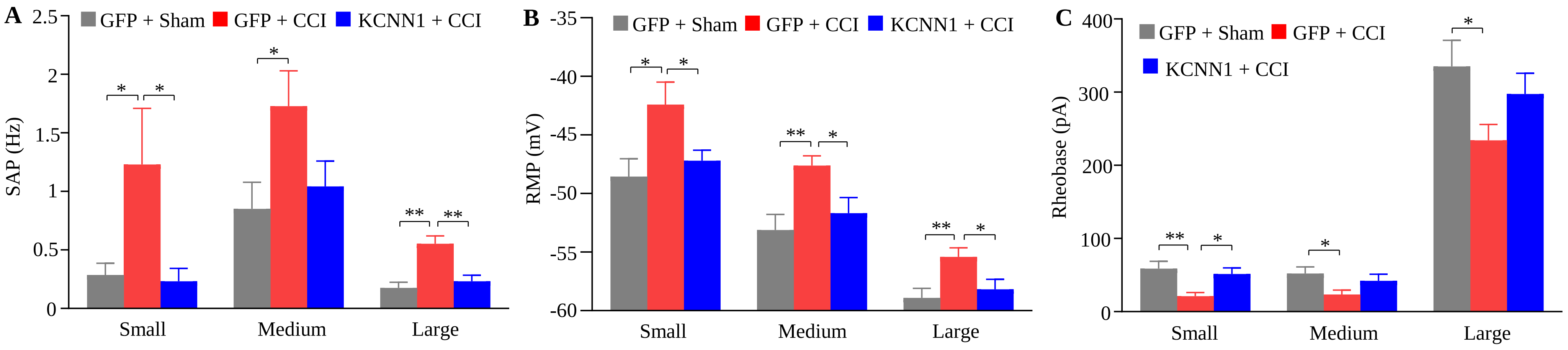

Supplementary Figure 5. Downregulated KCNN1 participates in the CCI-induced increase of the excitability in injured DRG neurons. All electrophysiological recordings were conducted in the ipsilateral L3/4 DRGs on day 7-10 after CCI or sham surgery in male mice with microinjection of AAV-9-KCNN1/-GFP (KCNN1) or AAV9-GFP (GFP) into the ipsilateral L3/4 DRG for 28 days. (A) Frequency of spontaneous action potentials (SAP) in small (n =25-36/group) medium (n = 30-32/group), and large (n = 32-35/group) DRG neurons from AAV9-GFP-microinjected sham mice (n = 22/size), AAV9-GFP-microinjected CCI mice (n = 22/size) or AAV9-KCNN1-/-GFP-microinjected CCI mice (n = 25/size). *P < 0.05, **P < 0.01 by two-way ANOVA with *post hoc* Tukey test. (B and C) Resting membrane potentials (RMP; B) and rheobases (C) in small, medium, and large DRG neurons from AAV9-GFP-microinjected sham mice, AAV9-GFP-microinjected CCI mice or AAV9-KCNN1-/-GFP-microinjected CCI mice. Number of neurons recorded/group/size was same as in A. *P < 0.05, **P < 0.01 by two-way ANOVA with *post hoc* Tukey test.

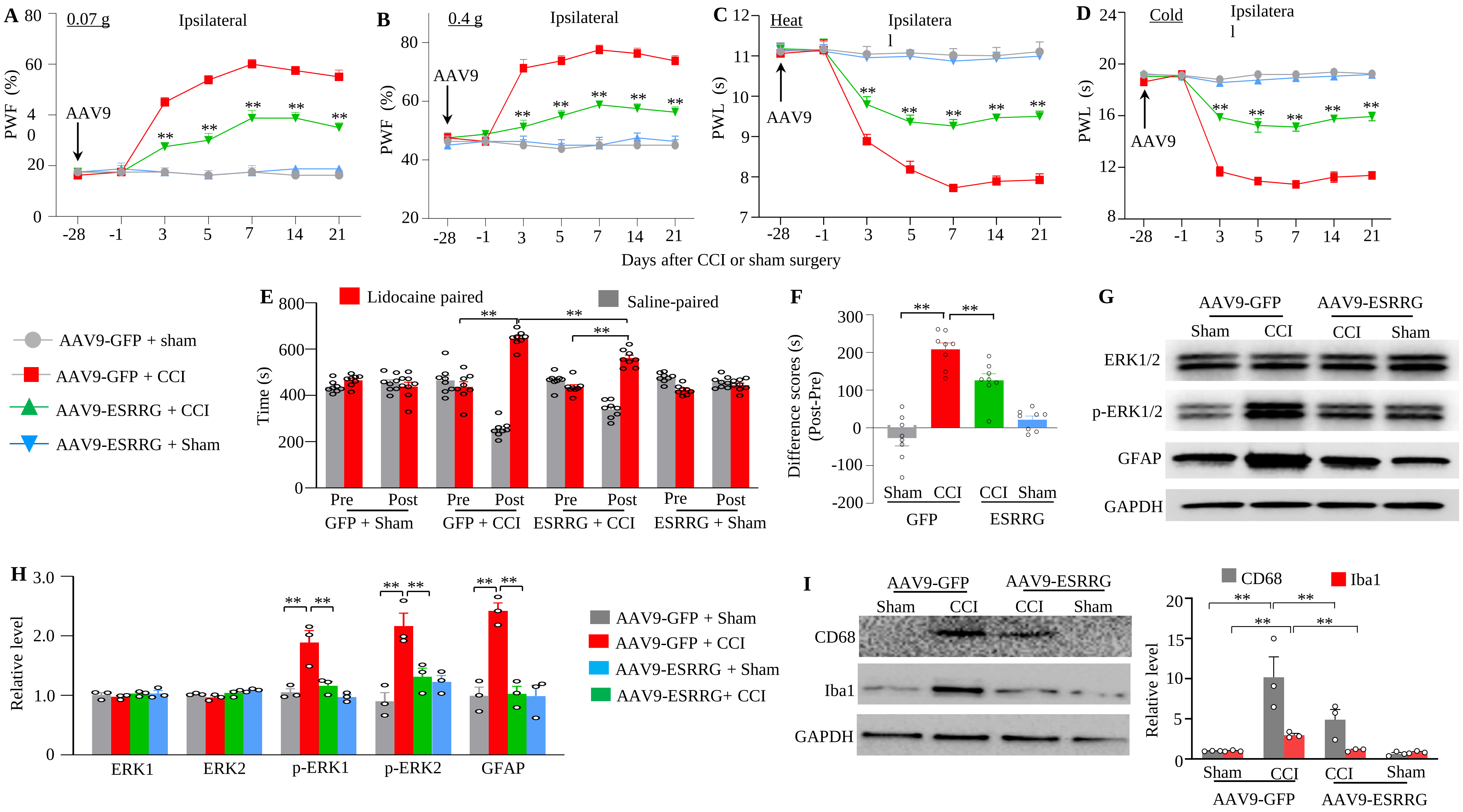

A
0.07 g
Ipsilateral
PWF (%)
AAV9
B
0.4 g
Ipsilateral
PWF (%)
AAV9
C
Heat
Ipsilatera l
PWL (s)
AAV9
D
Cold
Ipsilatera l
PWL (s)
AAV9
Days after CCI or sham surgery
AAV9-GFP + sham
AAV9-GFP + CCI
AAV9-ESRRG + CCI
AAV9-ESRRG + Sham
E
Lidocaine paired
Saline-paired
Time (s)
Pre Post Pre Post Pre Post Pre Post
GFP + Sham GFP + CCI ESRRG + CCI ESRRG + Sham
F
Difference scores (s) (Post-Pre)
Sham CCI CCI Sham
GFP ESRRG
G
AAV9-GFP AAV9-ESRRG
Sham CCI CCI Sham
ERK1/2
p-ERK1/2
GFAP
GAPDH
H
Relative level
ERK1 ERK2 p-ERK1 p-ERK2 GFAP
AAV9-GFP + Sham
AAV9-GFP + CCI
AAV9-ESRRG + Sham
AAV9-ESRRG+ CCI
I
AAV9-GFP AAV9-ESRRG
Sham CCI CCI Sham
CD68
Iba1
GAPDH
CD68 Iba1
Relative level
Sham CCI CCI Sham
AAV9-GFP AAV9-ESRRG

Supplementary Figure 6. Effect of rescuing ESRRG reduction in injured DRG on the development of CCI-induced nociceptive hypersensitivity in male mice. The mice were microinjected with AAV9-ESRRG (KCNN1) or AAV5-GFP (GFP) into the ipsilateral L3/4 DRGs 28 days before CCI or sham surgery. (A-D) Paw withdrawal frequencies (PWF) in response to 0.07 g (A) and 0.4 g (B) von Frey filament stimuli and paw withdrawal latencies (PWL) to heat (C) and cold (D) stimuli on the ipsilateral side at the different days as indicated after CCI or sham surgery. n = 8 mice/group. **$P < 0.01$ versus the AAV9-GFP-microinjected CCI group at the corresponding days by three-way ANOVA with repeated measures followed by post hoc Tukey test. (E and F) Spontaneous nociceptive responses as assessed by the CPP paradigm on day 21 post-CCI or sham surgery. Time spent in each chamber (E) and difference scores for chamber preferences calculated by subtracting preconditioning (Pre) preference time from postconditioning (Post) time spent in the lidocaine-paired chamber (F). n = 8 mice/group. **$P < 0.01$ by three-way ANOVA with repeated measures followed by post hoc Tukey test (E,) or by two-way ANOVA with repeated measures followed by post hoc Tukey test (F). (G-I) Expression of p-ERK1/2 (G, H), total ERK1/2 (G, H), GFAP (G. H), Iba1 (I), and CD68 (I) proteins in the ipsilateral L3/4 dorsal horn on day 21 after CCI or sham surgery. n = 3 repeats (6 mice)/group. **$P < 0.01$ by two-way ANOVA followed by post hoc Tukey test.

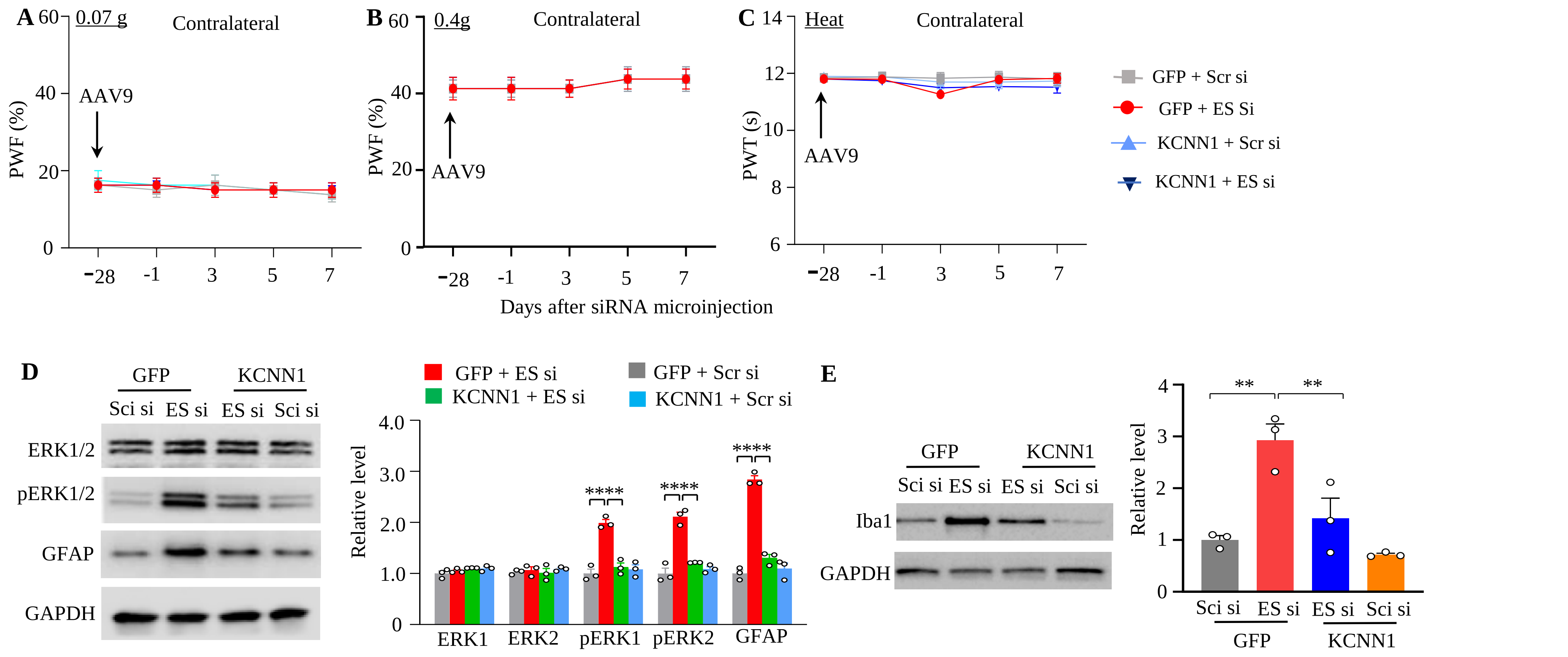

Supplementary Figure 7. The CCI-induced downregulation of KCNN1 is at least in part due to ESRRG reduction in injured DRG. (A-C) Paw withdrawal frequencies (PWF) in response to 0.07 g (A) and 0.4 g (B) von Frey filament stimuli and paw withdrawal latencies (PWL) to heat (C) stimulation on the contralateral side on the days as indicated after microinjection of *Esrrg* siRNA (ES si) or control scrambled siRNA (Scr si) into the ipsilateral L3/4 DRGs in male mice with microinjection of AAV9-KCNN1 (KCNN1) or AAV9-GFP (GFP) into unilateral L3/4 DRGs 28 days before siRNA injection. n = 8 mice/group. Two-way ANOVA with repeated measures followed by post hoc Tukey test. (D, E) Expression of p-ERK1/2 (D), total ERK1/2 (D), GFAP (D), and Iba1 (E) in the ipsilateral L3/4 dorsal horn on day 7 after microinjection of *Esrrg* siRNA (ES si) or control scrambled siRNA (Scr si) into the ipsilateral L3/4 DRGs in male mice with microinjection of AAV9-KCNN1 (KCNN1) or AAV9-GFP (GFP) into unilateral L3/4 DRGs 28 days before siRNA injection. n = 3 biological repeats (6 mice)/group. $^{**}P < 0.01$ by one-way ANOVA with repeated measures followed by post hoc Tukey test.

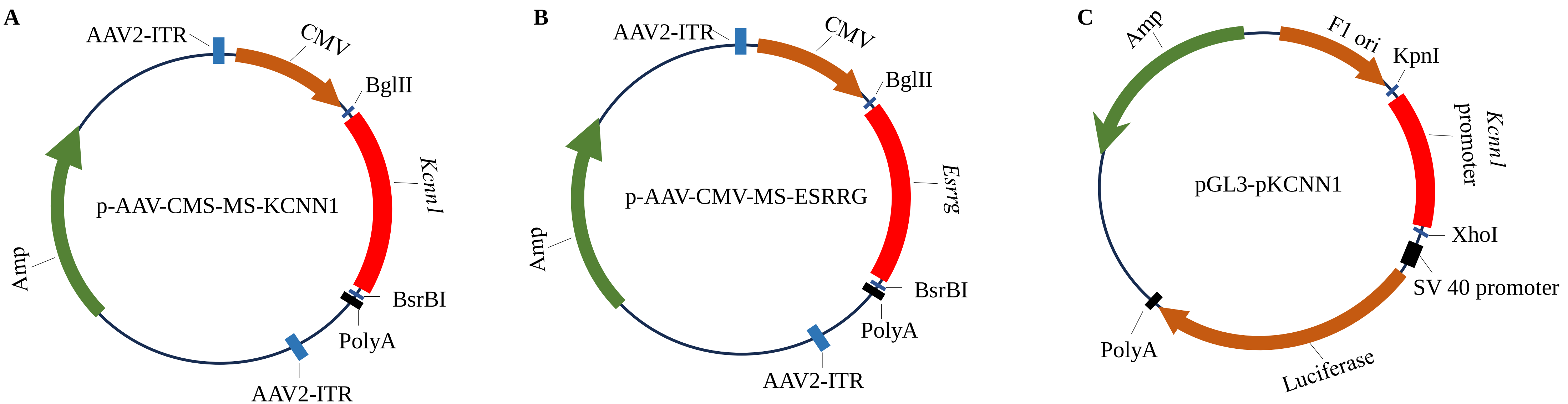

Supplementary Figure 8. A and B: Full-length *Kcnn1* cDNA (A) and full-length *Esrrg* cDNA (B) were amplified from mouse DRG RNA using PCR. PCR products were ligated into the BglII and BsrBI sites of the pAAV-CMV-MS vector to replace the enhanced green fluorescent protein (GFP) sequence. C: A 1,953-bp fragment from mouse *Kcnn1* promoter region (containing ESRRG-binding motifs) was amplified from mouse DRG RNA using PCR. The fragment was ligated into the KpnI and XhoI sites of pGL3 reporter vector. All sequences of recombinant clones were verified by DNA sequencing.

Supplementary Table 1: Locomotor function.

Treatment groups	Placing	Grasping	Righting
AAV9-KCNN1 + CCI （male）	5 (0)	5 (0)	5 (0)
AAV9-GFP + CCI （male）	5 (0)	5 (0)	5 (0)
AAV9- KCNN1 + Sham （male）	5 (0)	5 (0)	5 (0)
AAV9-GFP + Sham （male）	5 (0)	5 (0)	5 (0)
AAV9-KCNN1 + CCI （female）	5 (0)	5 (0)	5 (0)
AAV9-GFP + CCI （female）	5 (0)	5 (0)	5 (0)
AAV9-KCNN1*1* + Sham （female）	5 (0)	5 (0)	5 (0)
AAV9-GFP + Sham （female）	5 (0)	5 (0)	5 (0)
AAV9-*Kcnn1* siRNA（male）	5 (0)	5 (0)	5 (0)
AAV9-*Scrambled* siRNA（male）	5 (0)	5 (0)	5 (0)
AAV9-*Kcnn1* siRNA（female）	5 (0)	5 (0)	5 (0)
AAV9-*Scrambled* siRNA（female）	5 (0)	5 (0)	5 (0)
AAV9-ESRRG + CCI （male）	5 (0)	5 (0)	5 (0)
AAV9-GFP + CCI （male）	5 (0)	5 (0)	5 (0)
AAV9-ESRRG+ Sham （male）	5 (0)	5 (0)	5 (0)
AAV9-GFP + Sham （male）	5 (0)	5 (0)	5 (0)
AAV9-GFP + *Scrambled* siRNA（male）	5 (0)	5 (0)	5 (0)
AAV9-GFP + *Esrrg* siRNA（male）	5 (0)	5 (0)	5 (0)
AAV9-KCNN1 +*Esrrg* siRNA（male）	5 (0)	5 (0)	5 (0)
AAV9-KCNN1 + *Scrambled* siRNA（male）	5 (0)	5 (0)	5 (0)

Mean (SEM). n = 9 mice/group. 5 trials. AAV9: recombinant adeno-associated virus type 9. CCI: chronic constriction injury. ESRRG: estrogen-related receptor gamma. GFP: enhanced green fluorescent protein. KCNN1: Calcium-Activated Potassium Channel Subfamily N Member 1. siRNA: small interfering RNA.

Supplementary Table 2: Effect of rescuing DRG KCNN1 downregulation on membrane input resistance and other action potential parameters.

Grouping	GFP + Sham	GFP + CCI	KCNN1/GFP + CCI	F/p value
		Small		
n (Cells/Mice)	30/22	25/25	36/25	
R_{in} (MΩ)	482.1 ± 71.6	527.6 ± 80.7	509.2± 46.6	0.12/0.89
APA (mV)	93.1 ± 2.2	97.6 ± 1.95	98.3± 3.6	0.91/0.41
APT (mV)	-22.1 ±1.0	-23.9 ± 1.2	-23.3± 0.8	0.93/0.40
APO (mV)	39.4 ± 1.8	38.7 ± 2.03	39.6± 1.6	0.06/0.94
		Medium		
n (Cells/Mice)	31/22	30/25	32/26	
R_{in} (MΩ)	398.7.1 ± 33.3	466.6 ± 57.3	411.2 ± 28.8	0.76/0.47
APA (mV)	93.5 ± 1.8	92.8 ± 1.6	95.8 ± 2.9	0.53/0.59
APT (mV)	-23.0 ± 1.1	-23.2 ± 1.1	-23.3 ± 0.6	0.24/0.79
APO (mV)	41.9 ± 1.5	39.1 ± 1.8	39.7 ± 1.8	0.77/0.47
		Large		
n (Cells/Mice)	34/21	35/25	32/26	
R_{in} (MΩ)	214.5 ± 15.6	216.1 ± 16	229.6 ± 15.9	0.27/0.77
APA (mV)	92.7 ± 1.9	95.5 ± 1.7	94.2 ± 3.5	0.84/0.44
APT (mV)	-20.8 ± 0.7	-22.1 ± 0.9	-19.8 ± 0.7	0.87/0.42
APO (mV)	39.2 ± 1.7	38.4± 1.8	39.8 ± 1.8	0.08/0.92

GFP: AAV9-GFP. KCNN1/GFP: a mixed solution of AAV9-KCNN1 and AAV5-GFP. APA: action potential amplitude. APT: action potential threshold. APO: action potential overshoot.

Supplementary Table 3: Primers and siRNAs used

Names	Sequences
**Real-time RT-PCR**	
Tuba1a -F	5′-GTGCATCTCCATCCATGTTG-3′
Tuba1a -R	5′-CAGCTGCTCCACCTTCTTCT-3′
Kcnn1 -F	5′-TTGAAAAGCGTAAACGGCTCA-3′
Kcnn1 -R	5′-CAGAGCAAAAGAGCAGAGTGA-3′
Essrg -F	5′-TGTTGGTGGCTGAACCAGAG-3′
Essrg -R	5′-CAACGCCGAGGATCAGAATC-3′
**Vector construction**	
AAV9- *Kcnn1* -F	5′-GTCAGATCTATGAGTAGCCACAGCCACAATGGCA-3′
AAV9- *Kcnn1* -R	5′-CCGCTCGAT TCACCCACAGTCTGATCCATGGTGGGC-3′
AAV9-- *Esrrg* -F	5′-GGGAGATCTATGTCAACAAAGATCGACACATT-3′
AAV9- *Esrrg*-R	5′-CCGCTCGATCAGACCTTGGCCTCCAGCATTTC-3′
**ChIP assay**	
Kcnn1-F	5′- TGAAAAGCGTAAACGGCTC -3′
Kcnn1-R	5′- CAGAGCAAAAGAGCAGAGTG -3′
**Luciferase assay**	
Kcnn1-F	5′-ACTGGTACCGAAACCTGCCCGGCATTTGTAG-3′
Kcnn1-R	5′-GATCTCGAGGGATTCTGGTGAAACATTTGGG-3′
**siRNAs**	
Kcnn1 siRNA	5′-UUUUCAAAGAGGGCCCUGCGG-3′
Esrrg siRNA	5′-UUAUGGGCUGUUCUCUGCAUCUAT-3′
Control scrambled siRNA	5′-GGUUCAGAUGUGCGGCGAGU-3′

F: Forward. R: Reverse. *Tuba1a:* Tubulin alpha 1a. *Kcnn1*: Potassium Calcium-Activated Channel *Subfamily N Member 1*. *Esrrg:* estrogen Related Receptor Gamma. siRNA: small interfering RNA.
