# Supplementary material for: ESRRG-controlled downregulation of KCNN1 in primary sensory neurons is required for neuropathic pain
Source: JCI Insight. 2024 May 21;9(12):e180085. doi: 10.1172/jci.insight.180085 (PMC11383585; doi:10.1172/jci.insight.180085)

Full unedited gels for Fig. 2B

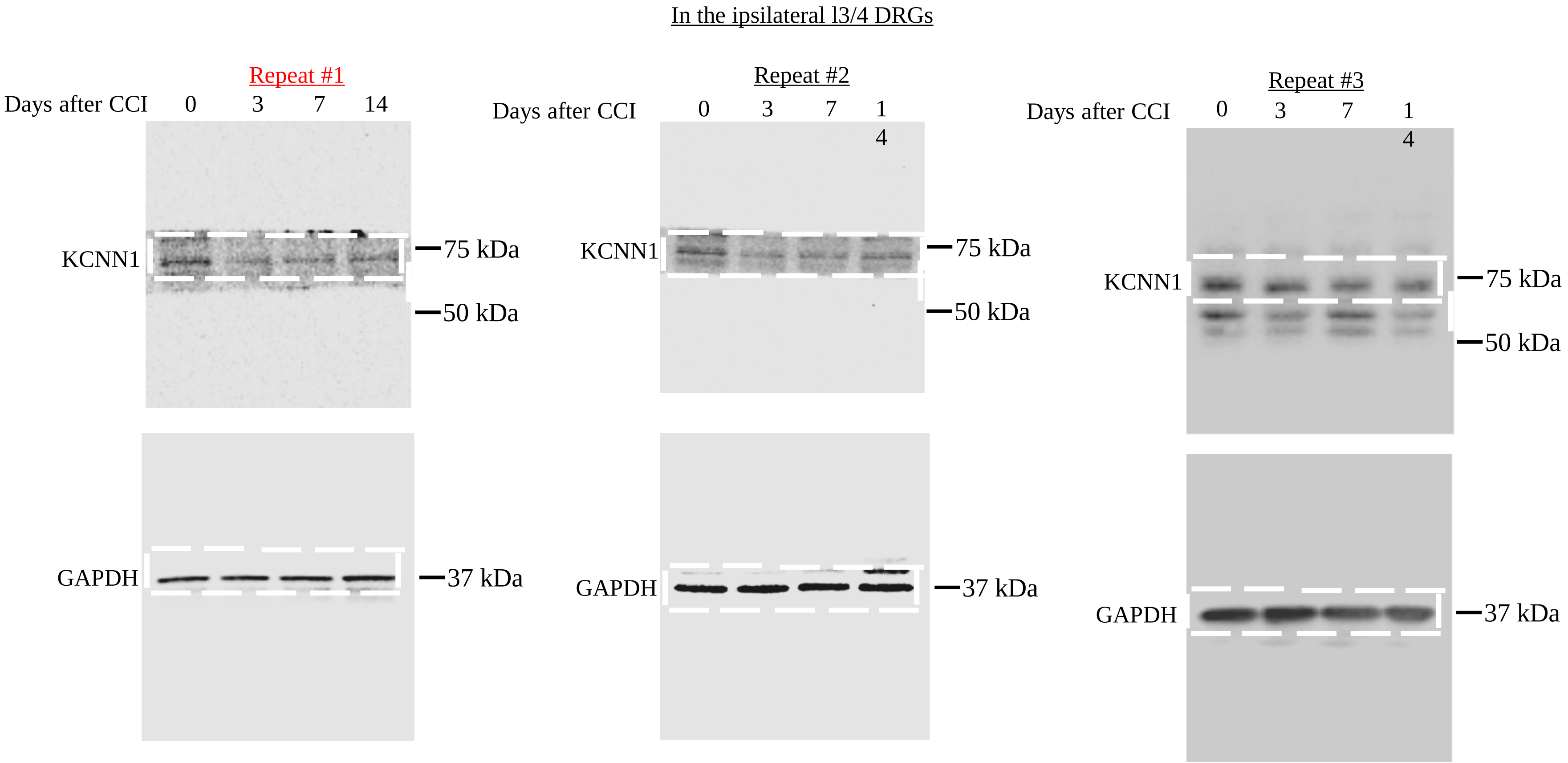

Full unedited gels for Fig. 2B

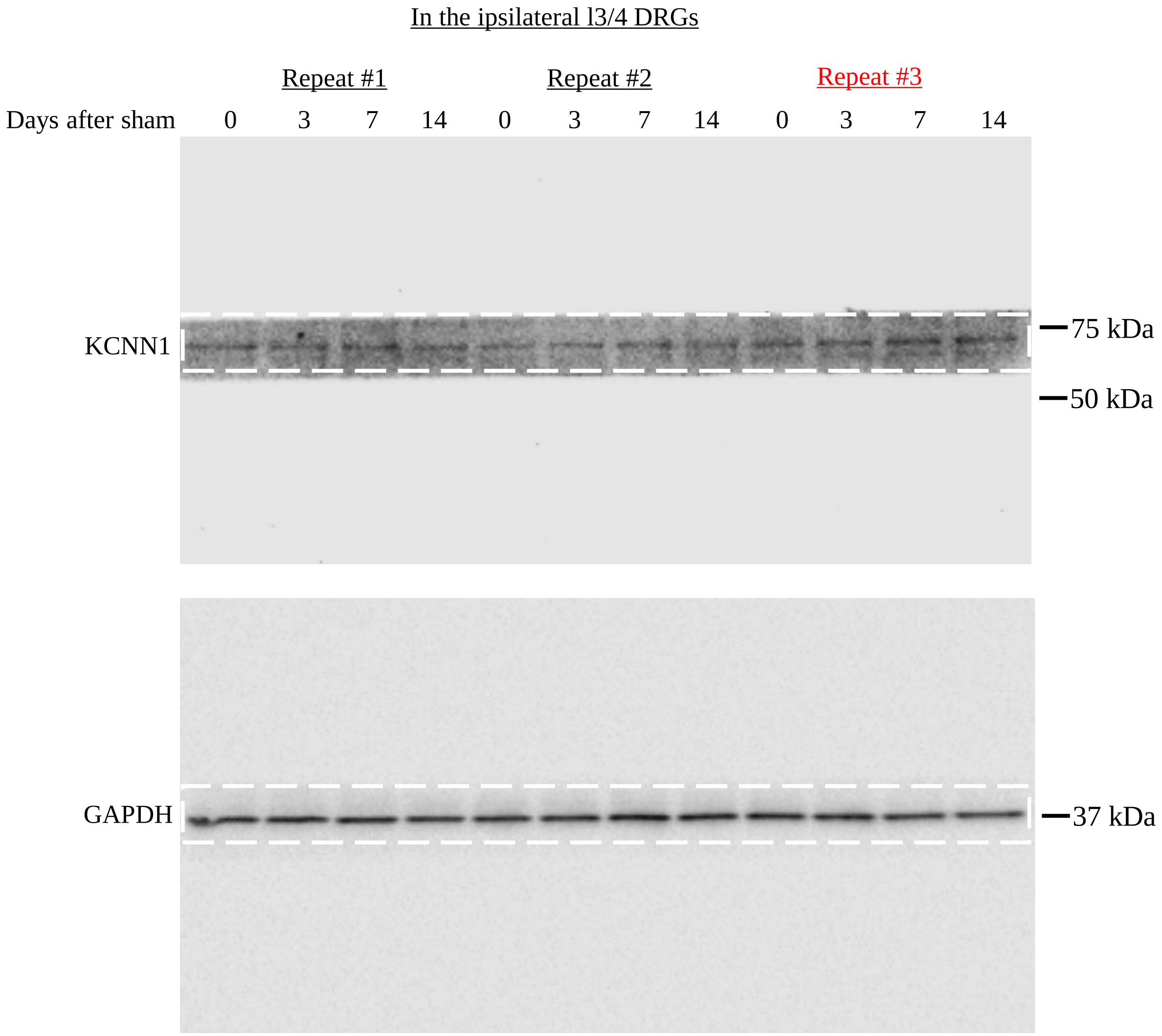

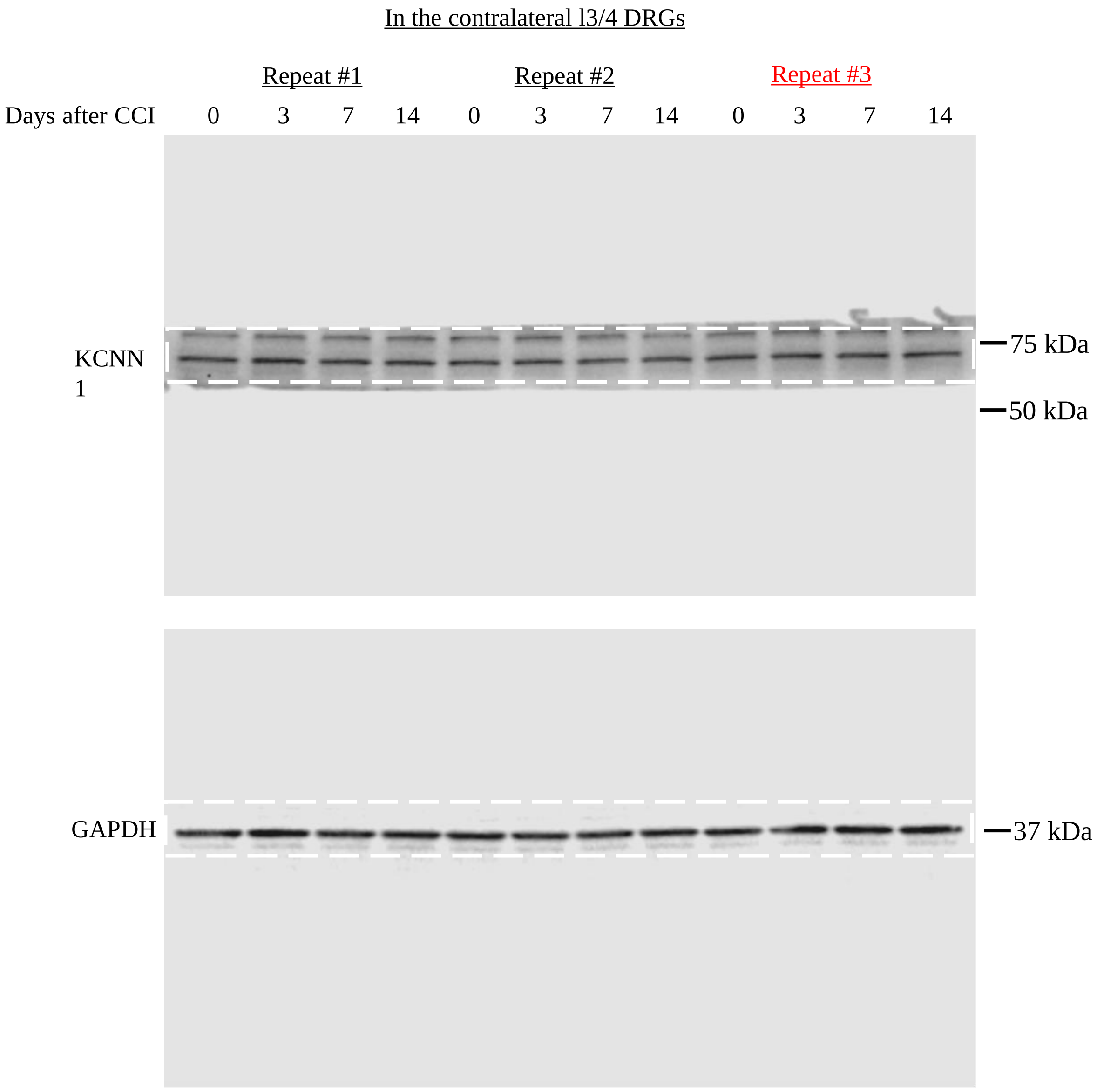

Full unedited gels for Fig. 2B

In the ipsilateral l3/4 spinal cord

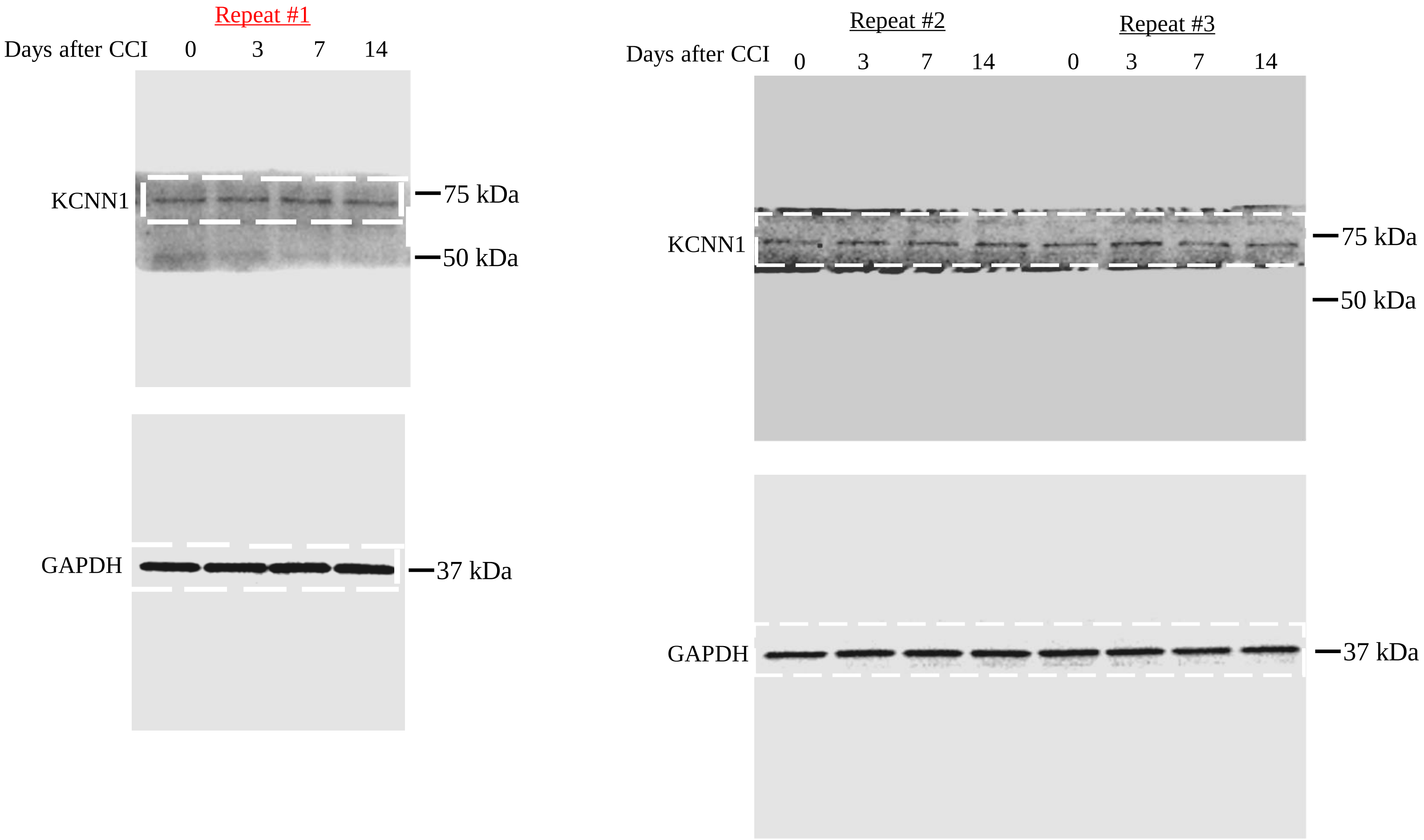

Full unedited gels for Fig. 2D

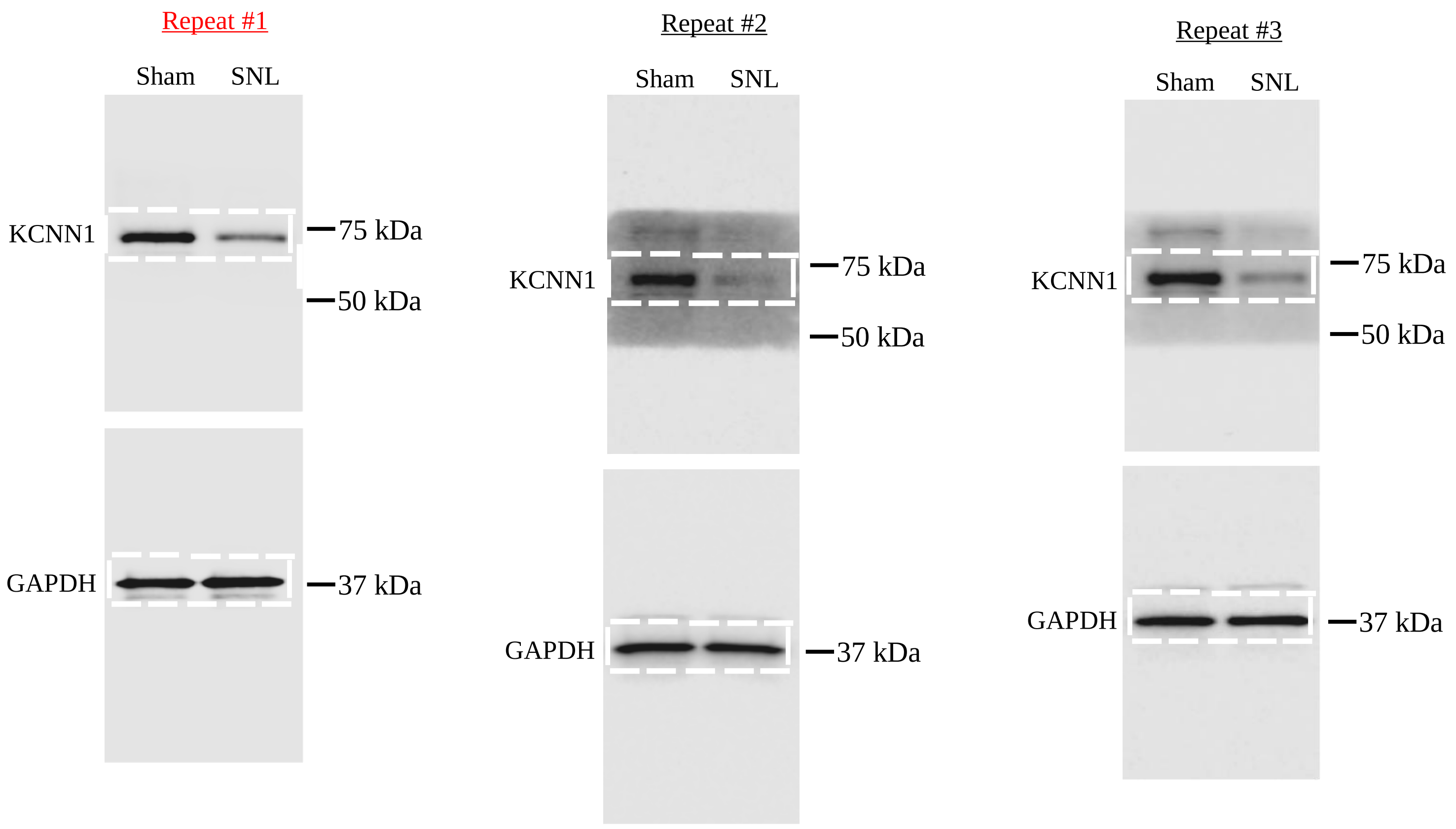

Full unedited gels for Fig. 3A

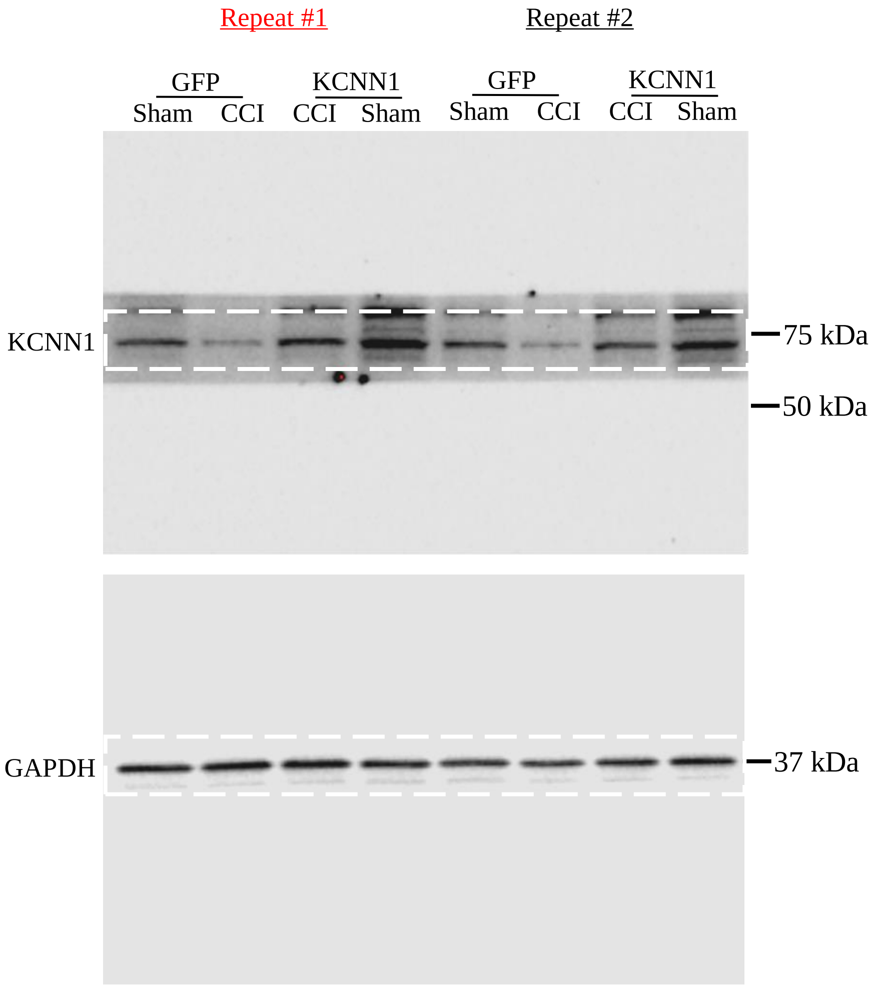

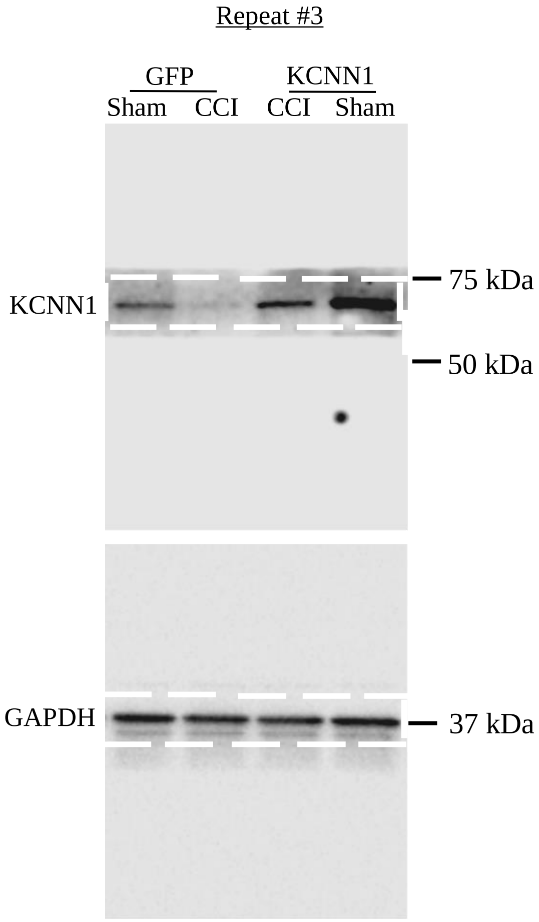

Full unedited gels for Fig. 3K

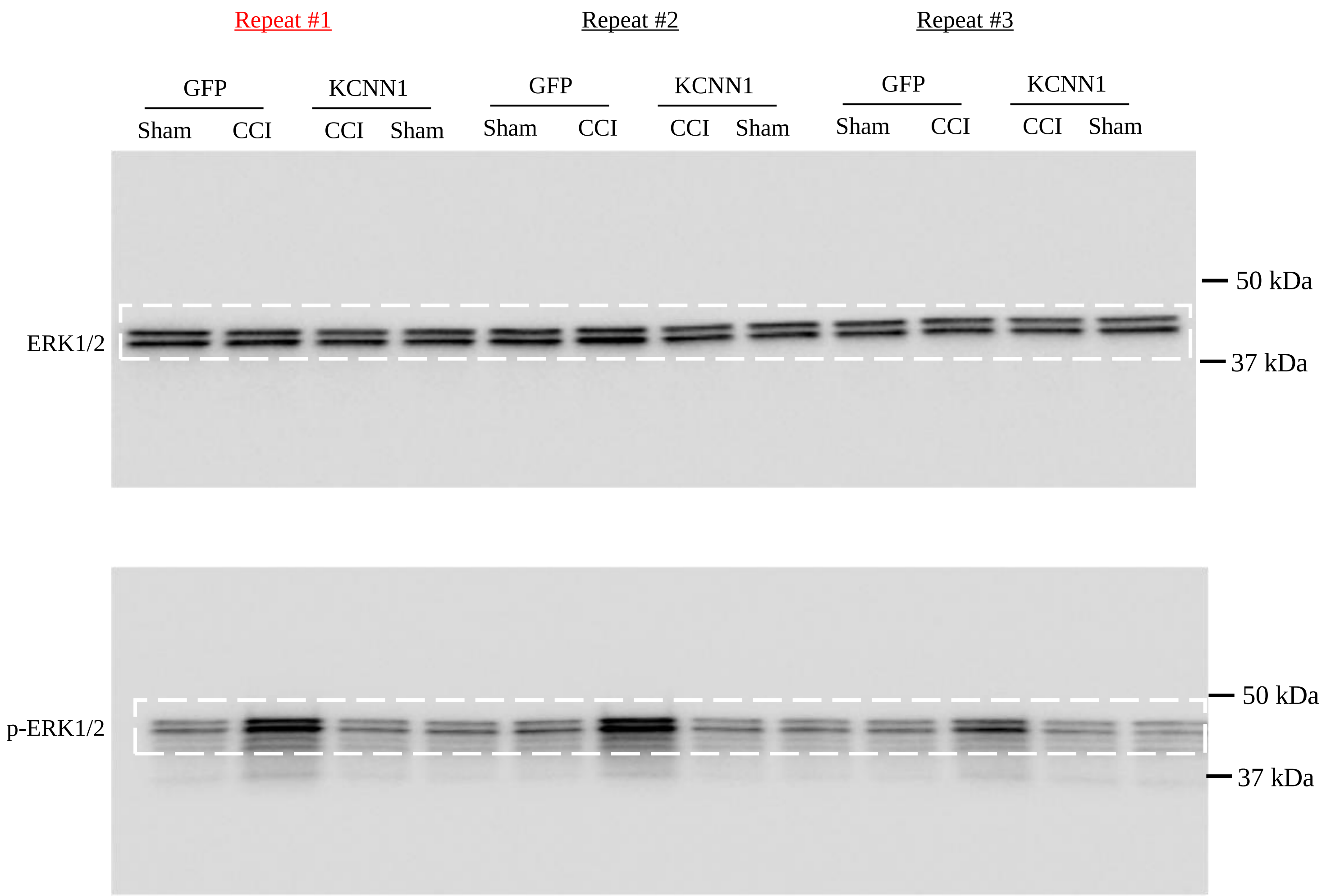

Full unedited gels for Fig. 3K

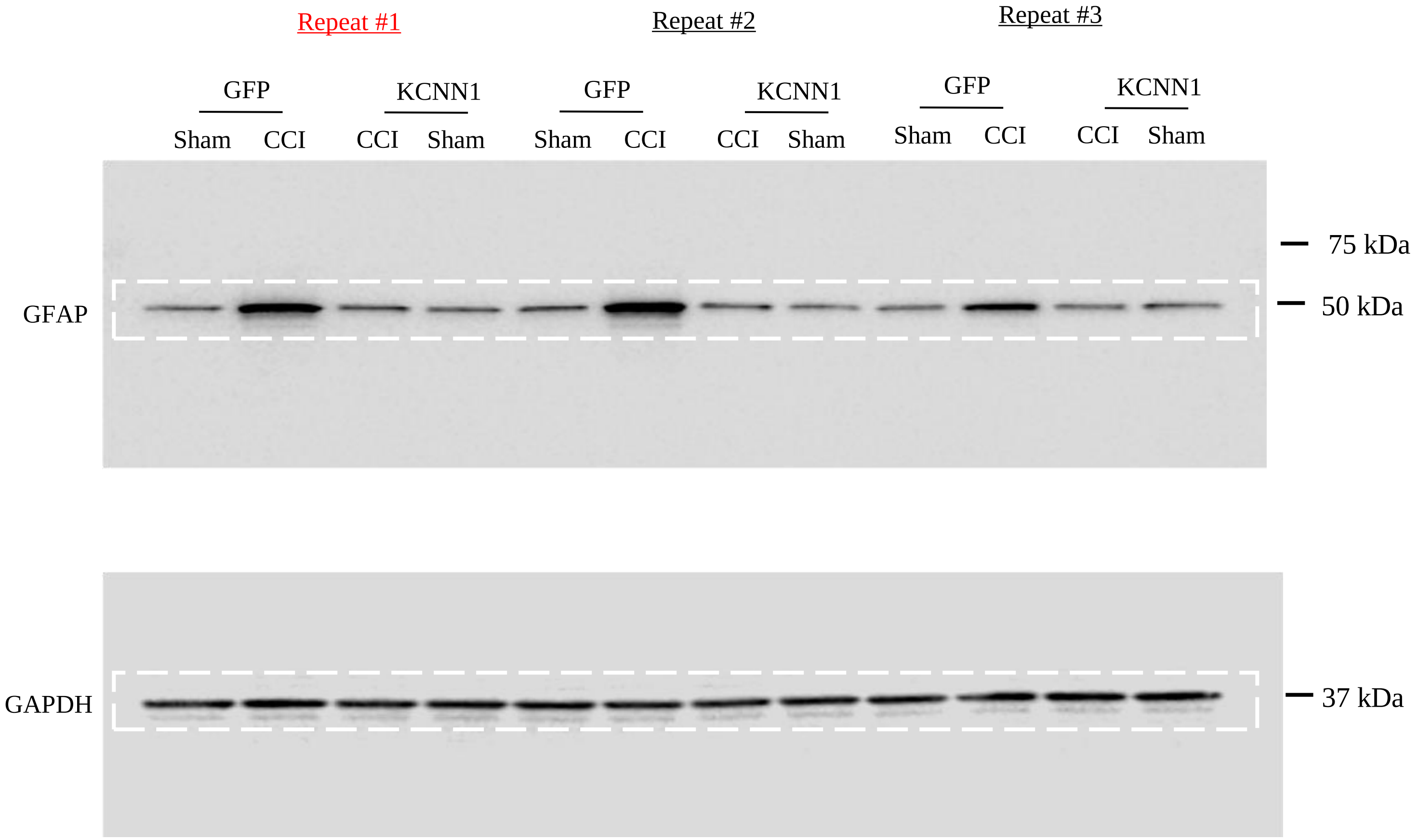

Full unedited gels for Fig. 3L

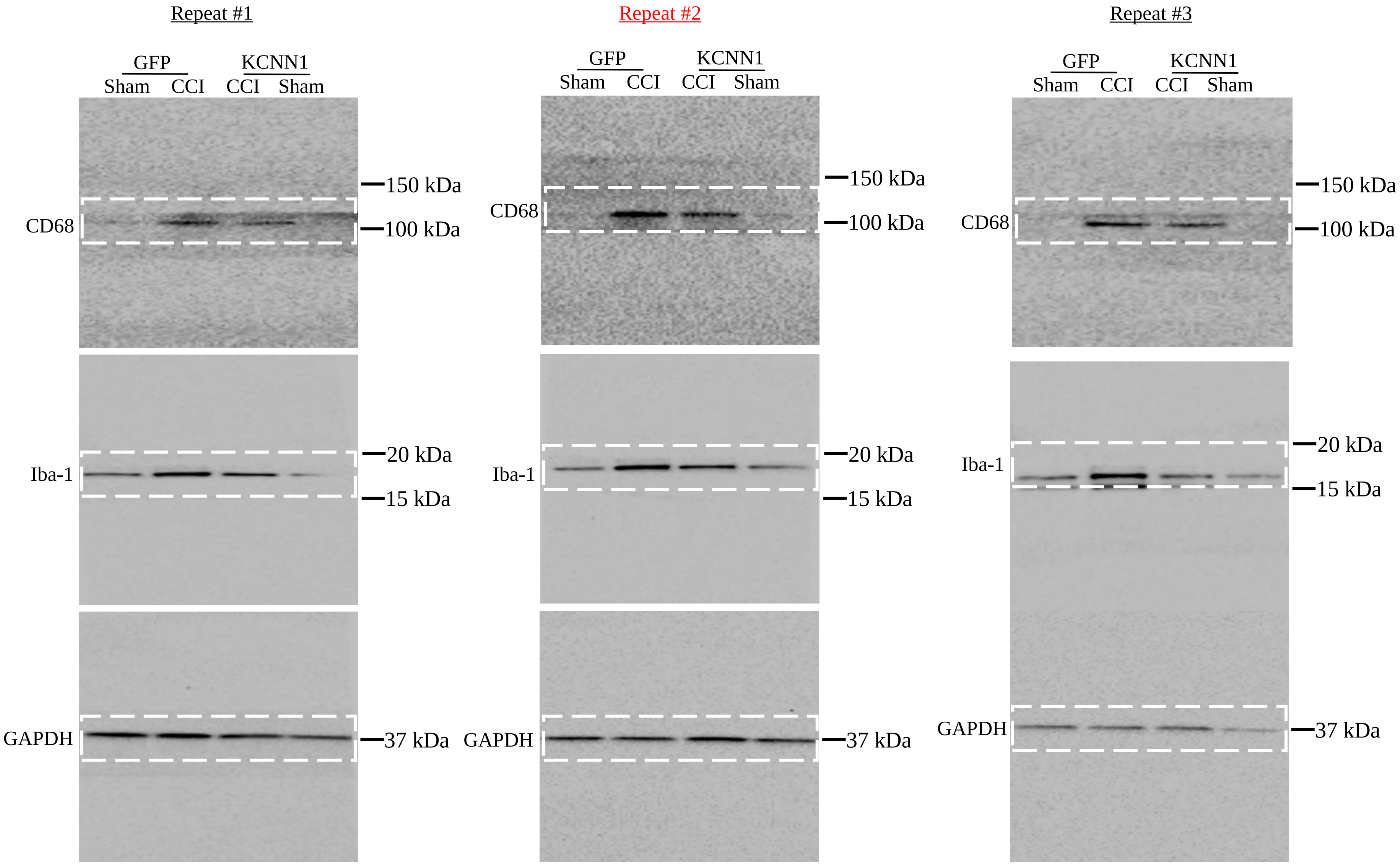

Full unedited gels for Fig. 4G

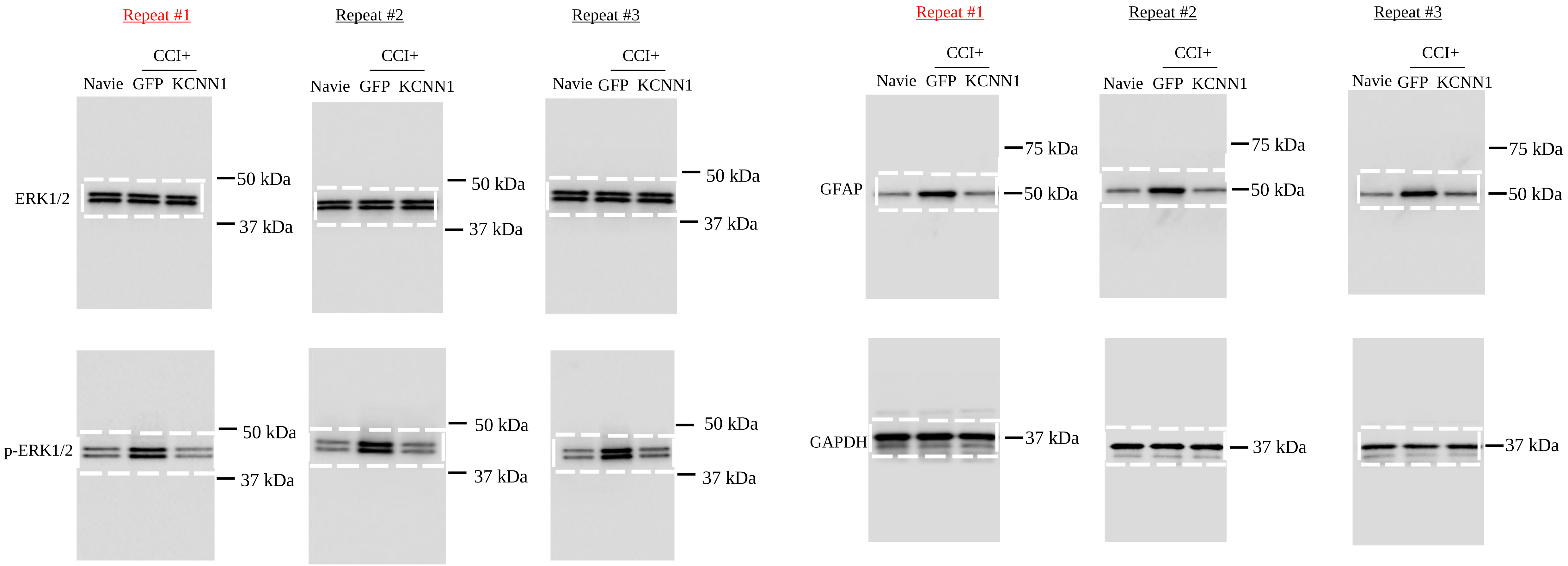

Full unedited gels for Fig. 4H

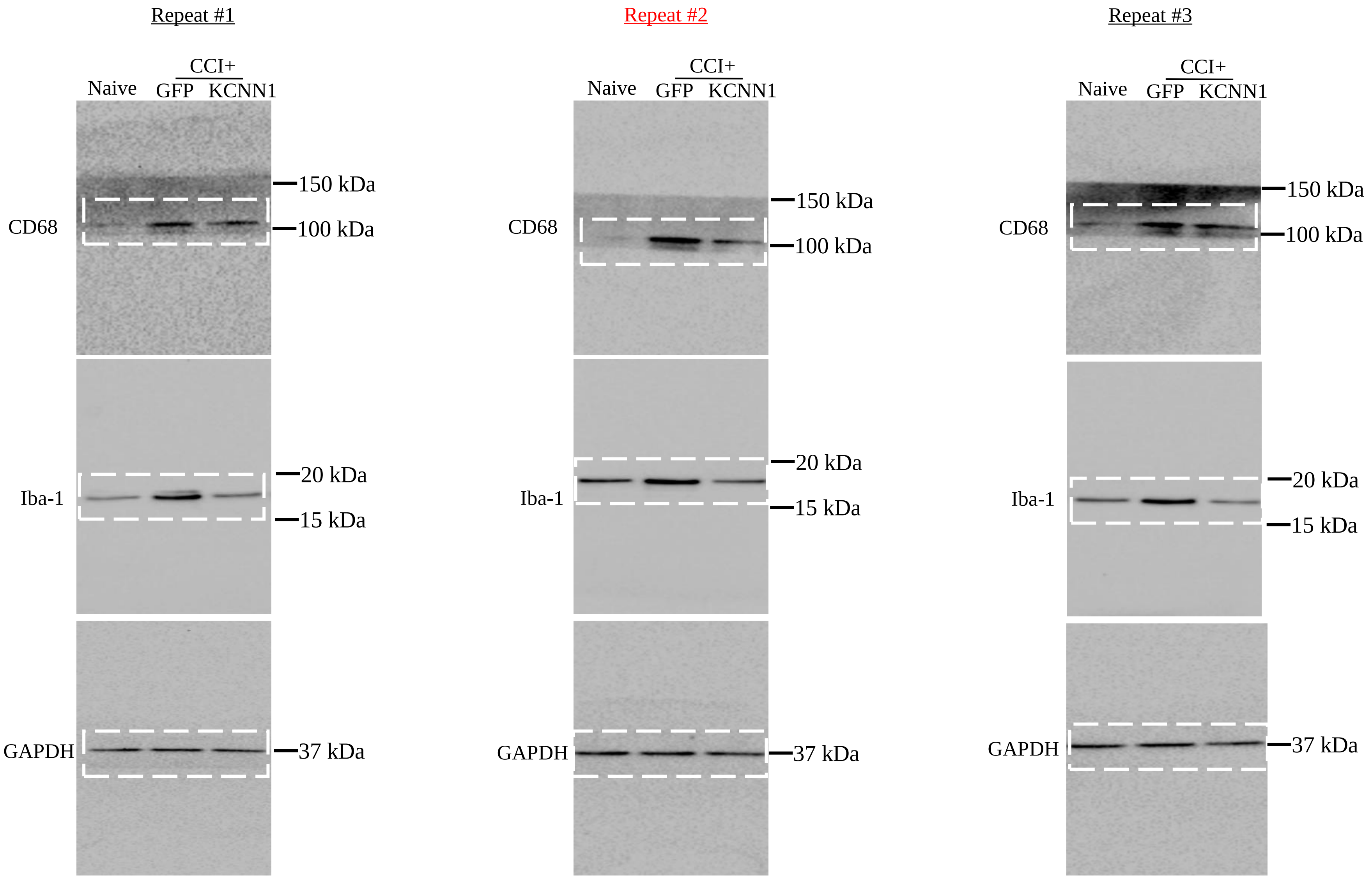

Full unedited gels for Fig. 4J

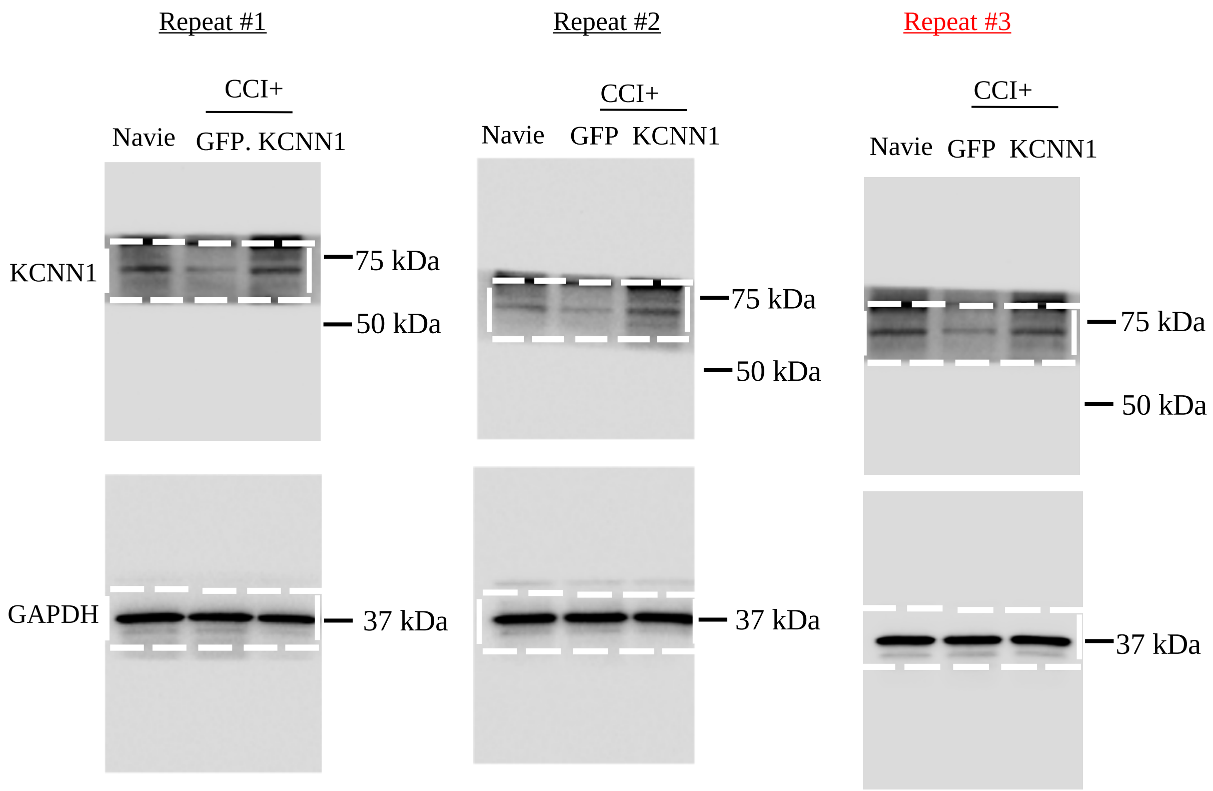

Full unedited gels for Fig. 5B

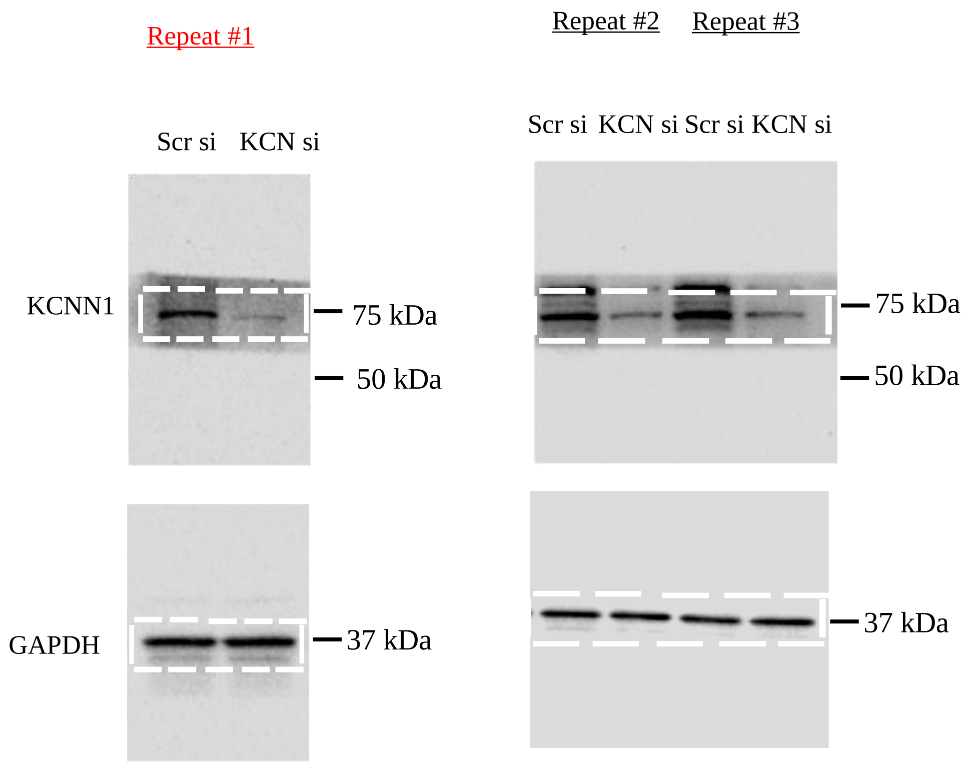

Full unedited gels for Fig. 5C

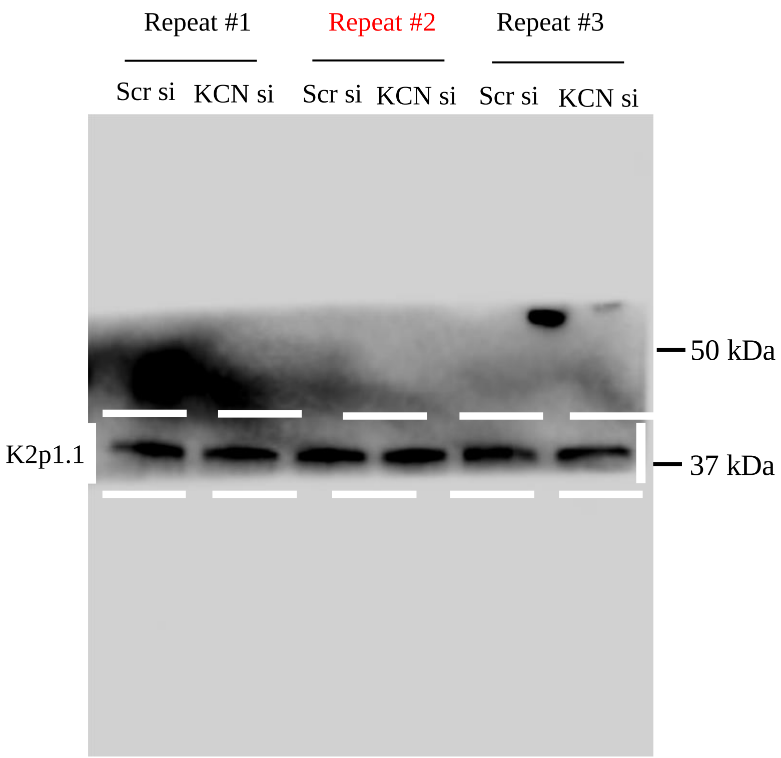

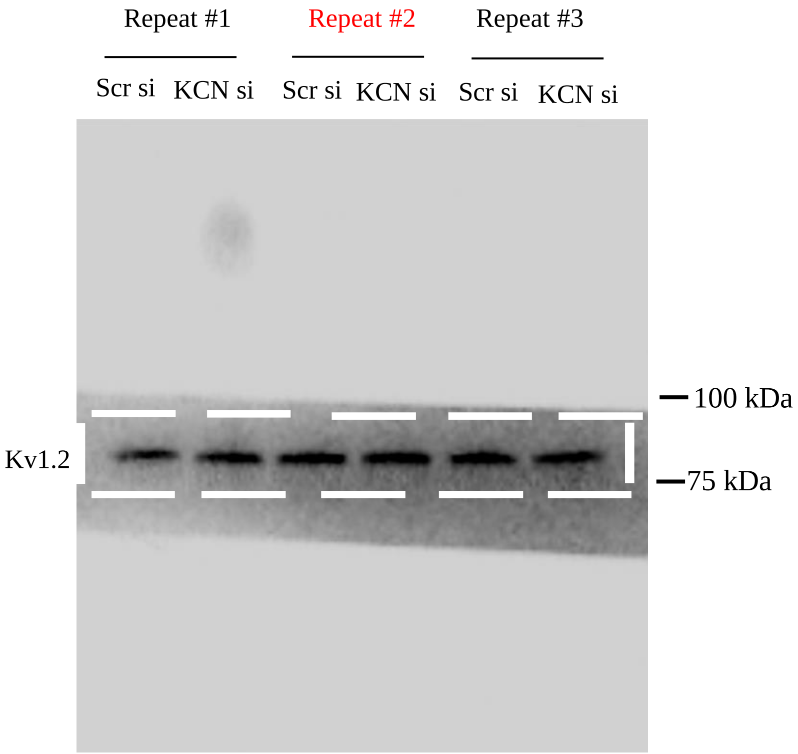

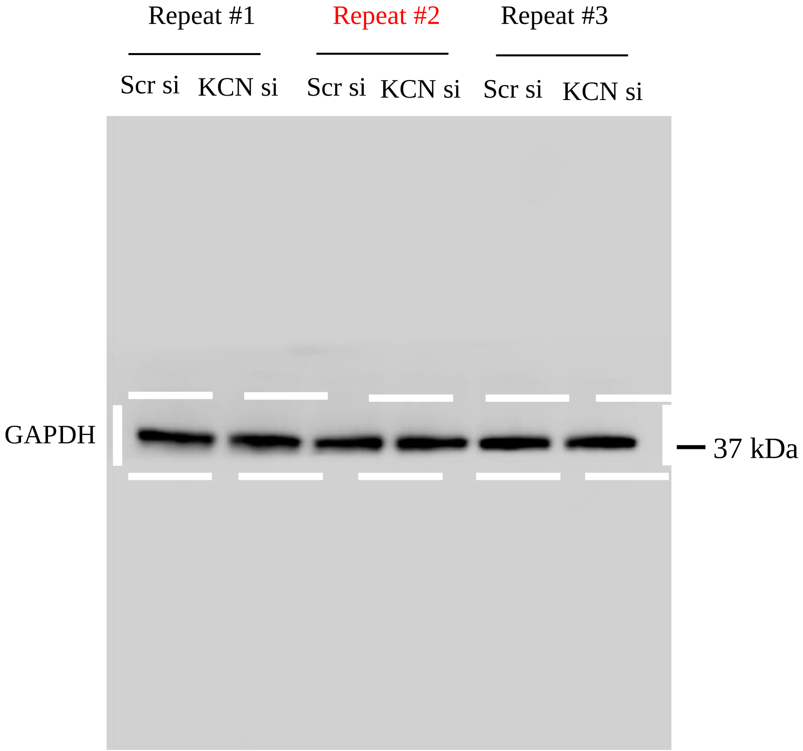

Full unedited gels for Fig. 5H

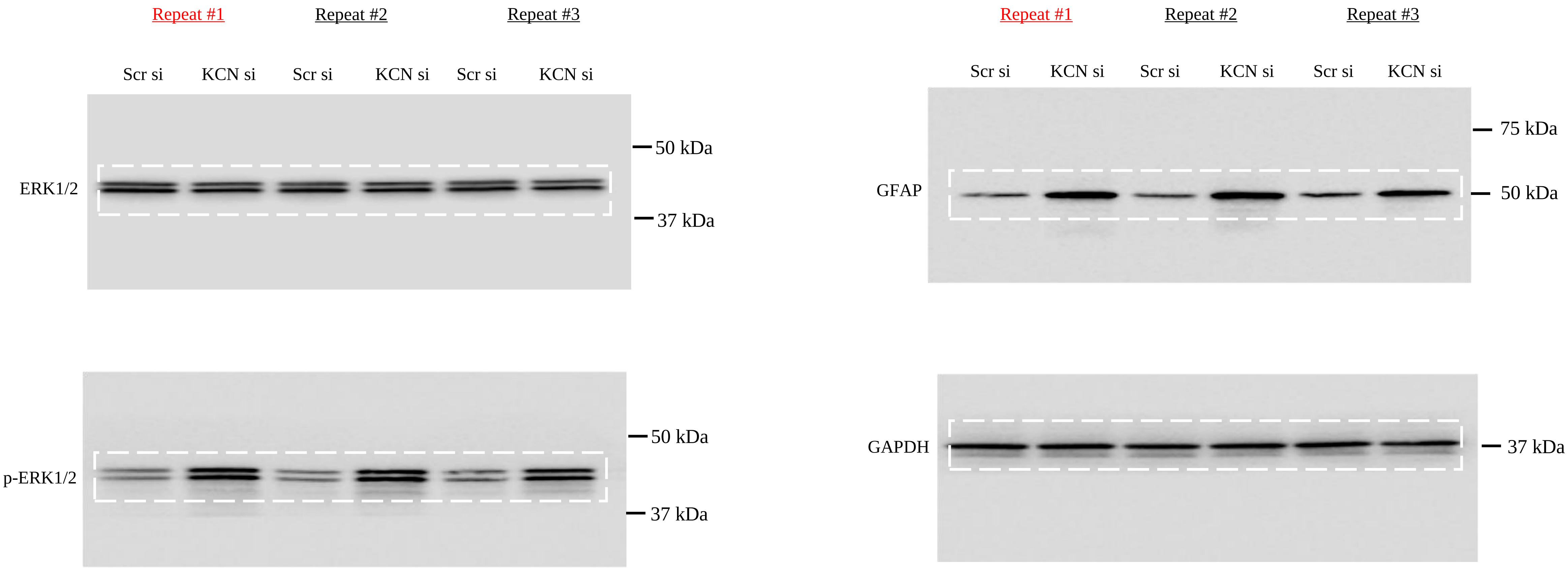

Full unedited gels for Fig. 5I

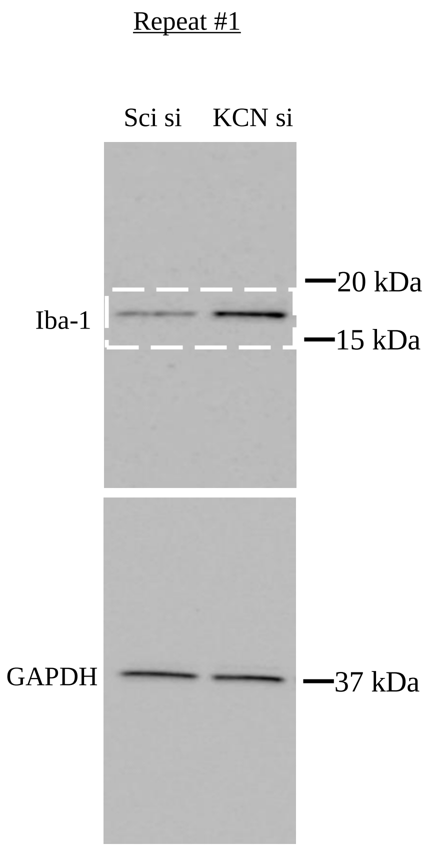

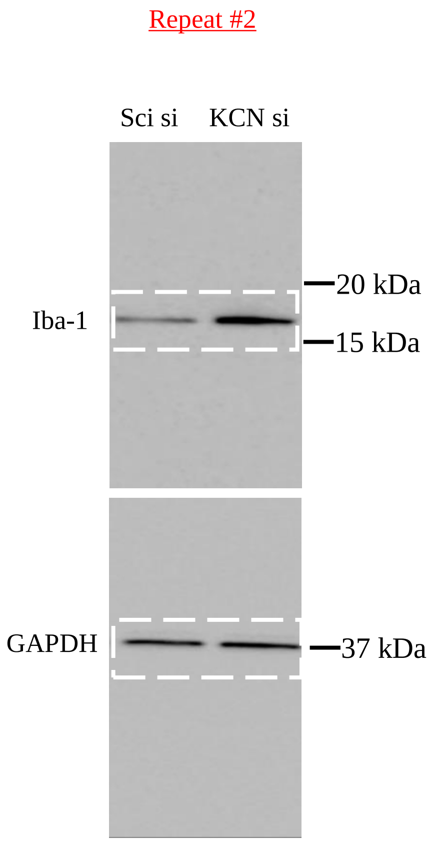

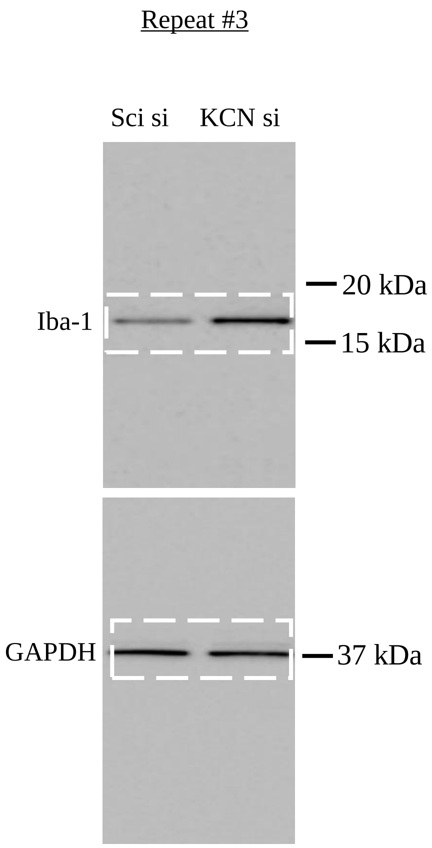

Full unedited gels for Fig. 7C

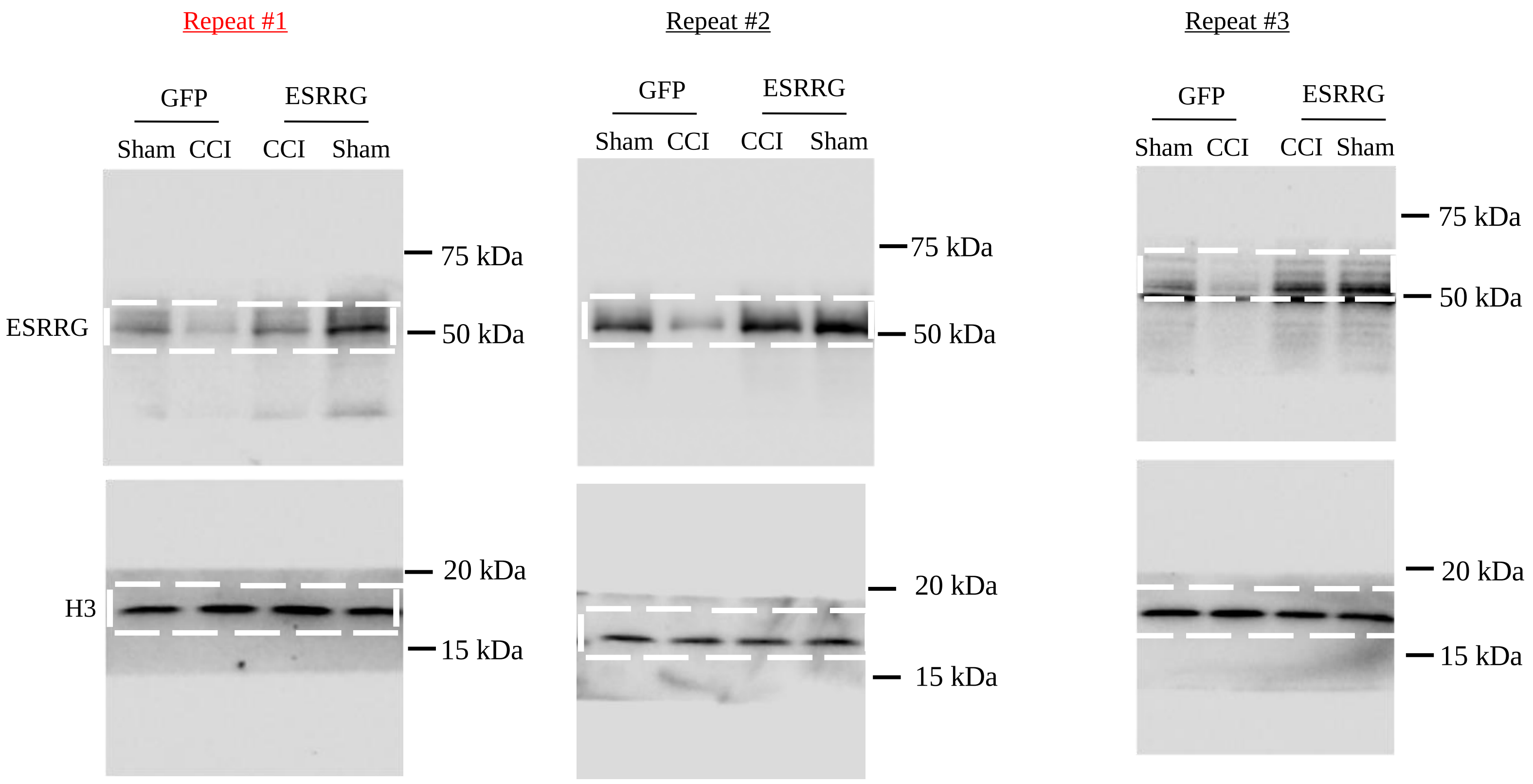

Full unedited gels for Fig. 7C

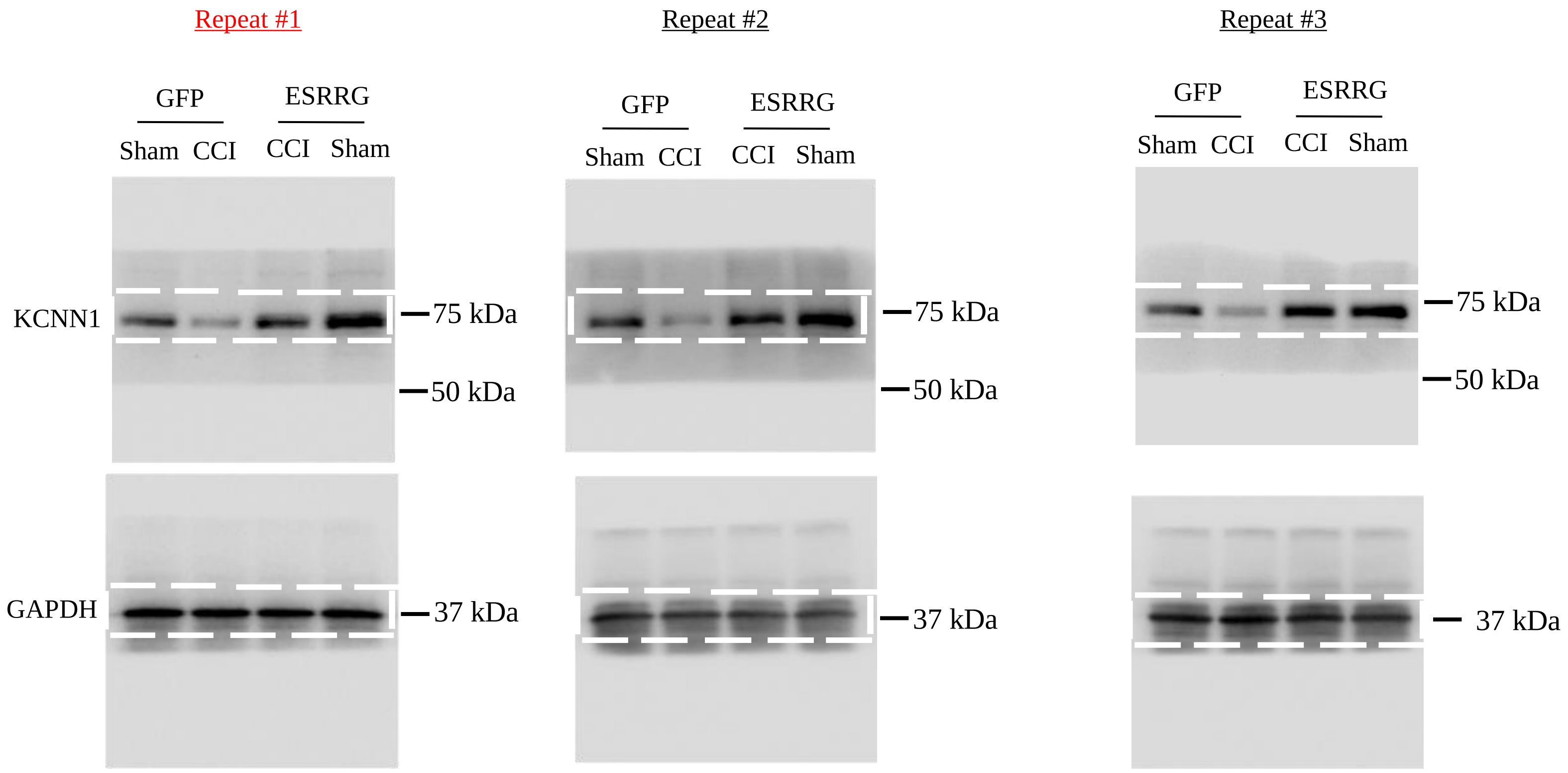

Full unedited gels for Fig. 7E

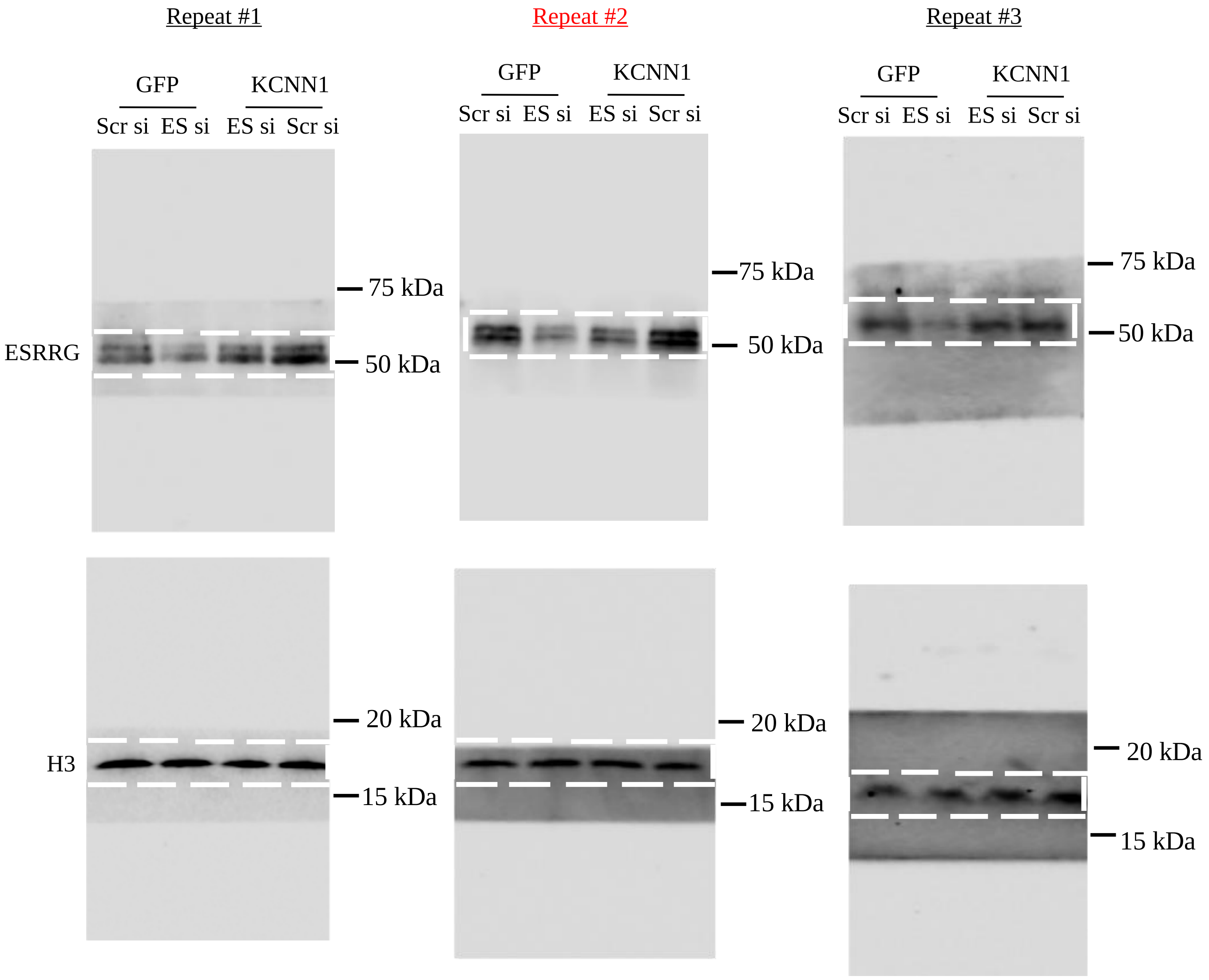

Full unedited gels for Fig. 7E

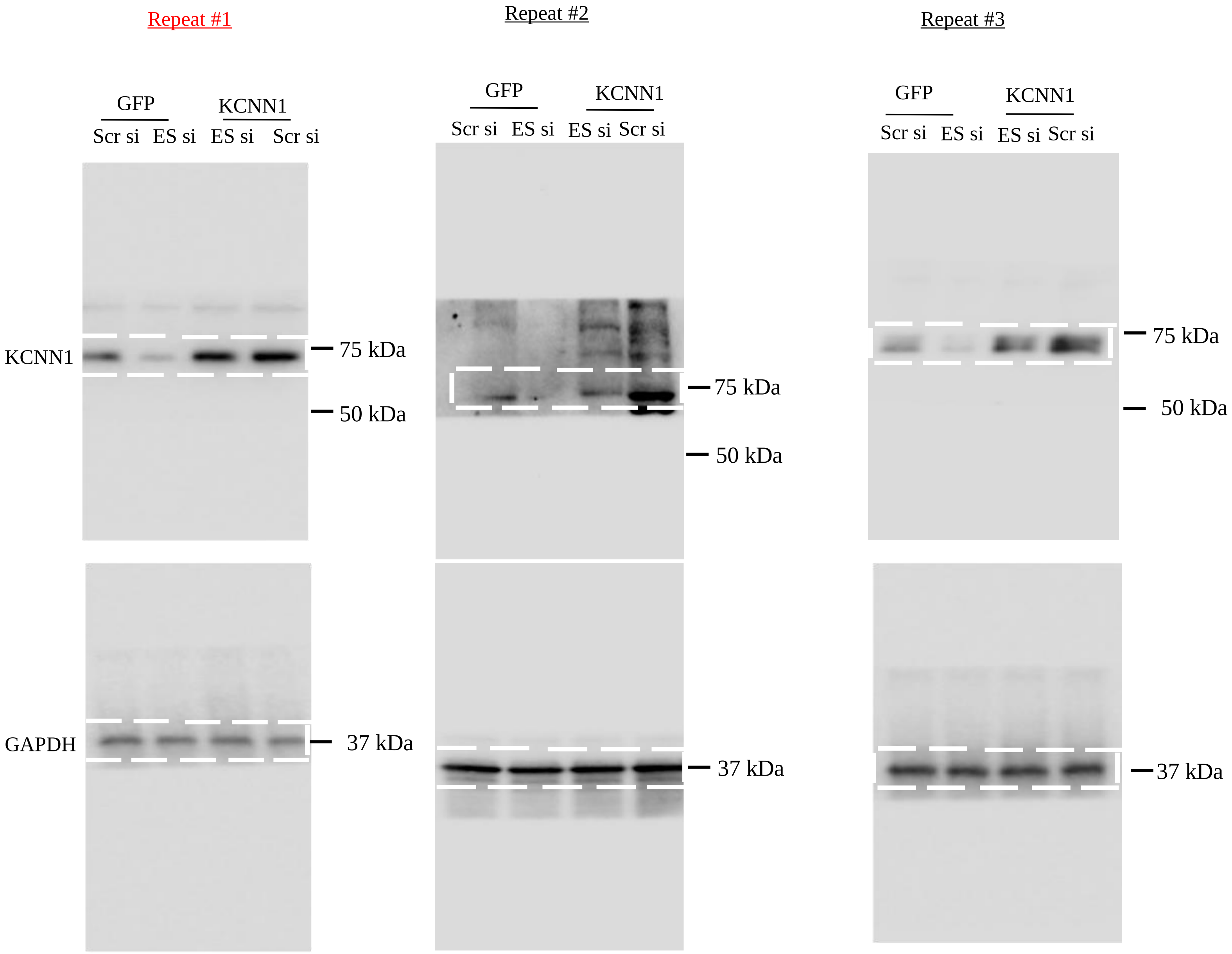

Full unedited gels for supplementary Fig. 2

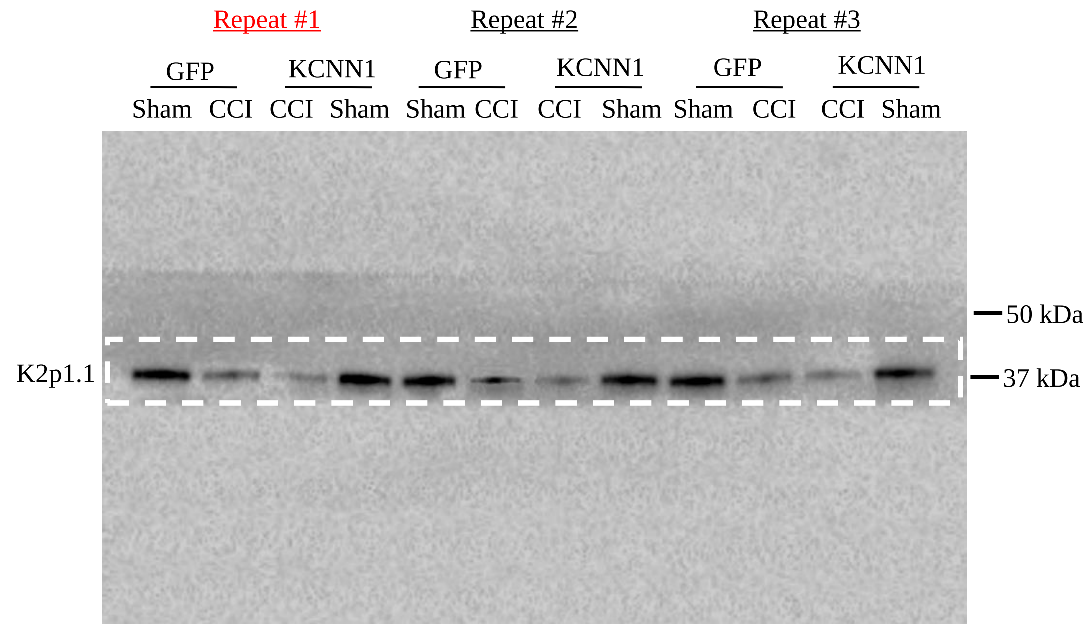

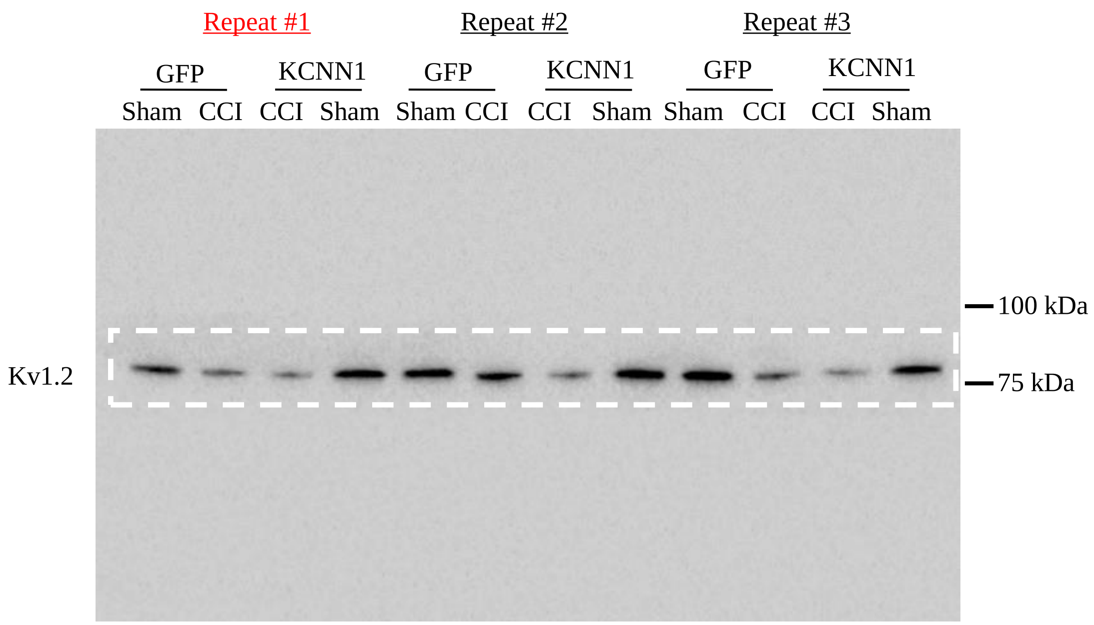

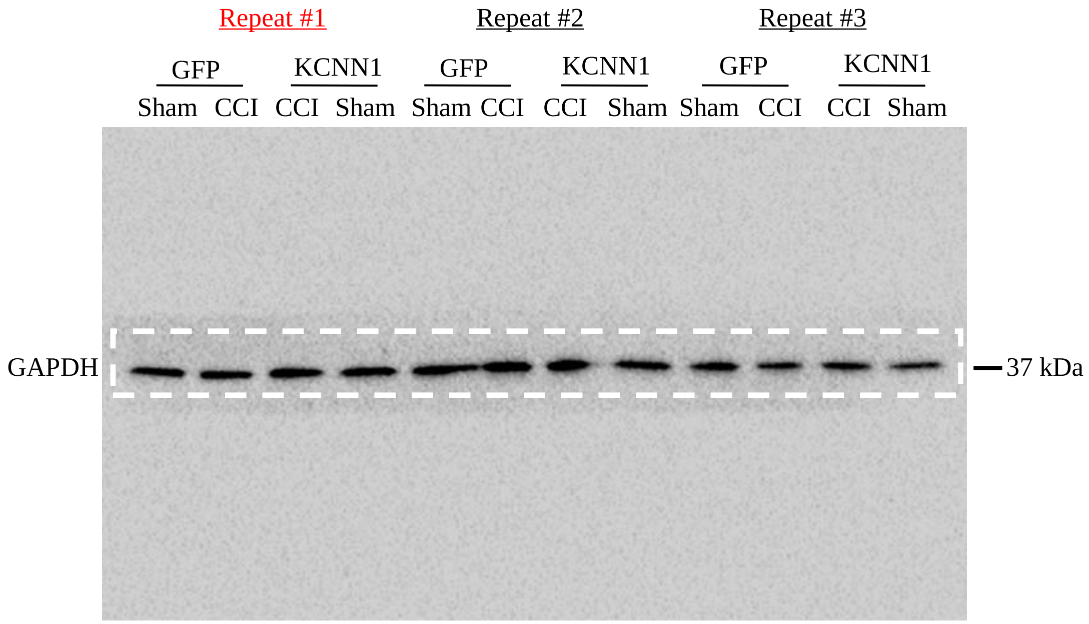

Full unedited gels for supplementary Fig. 3A

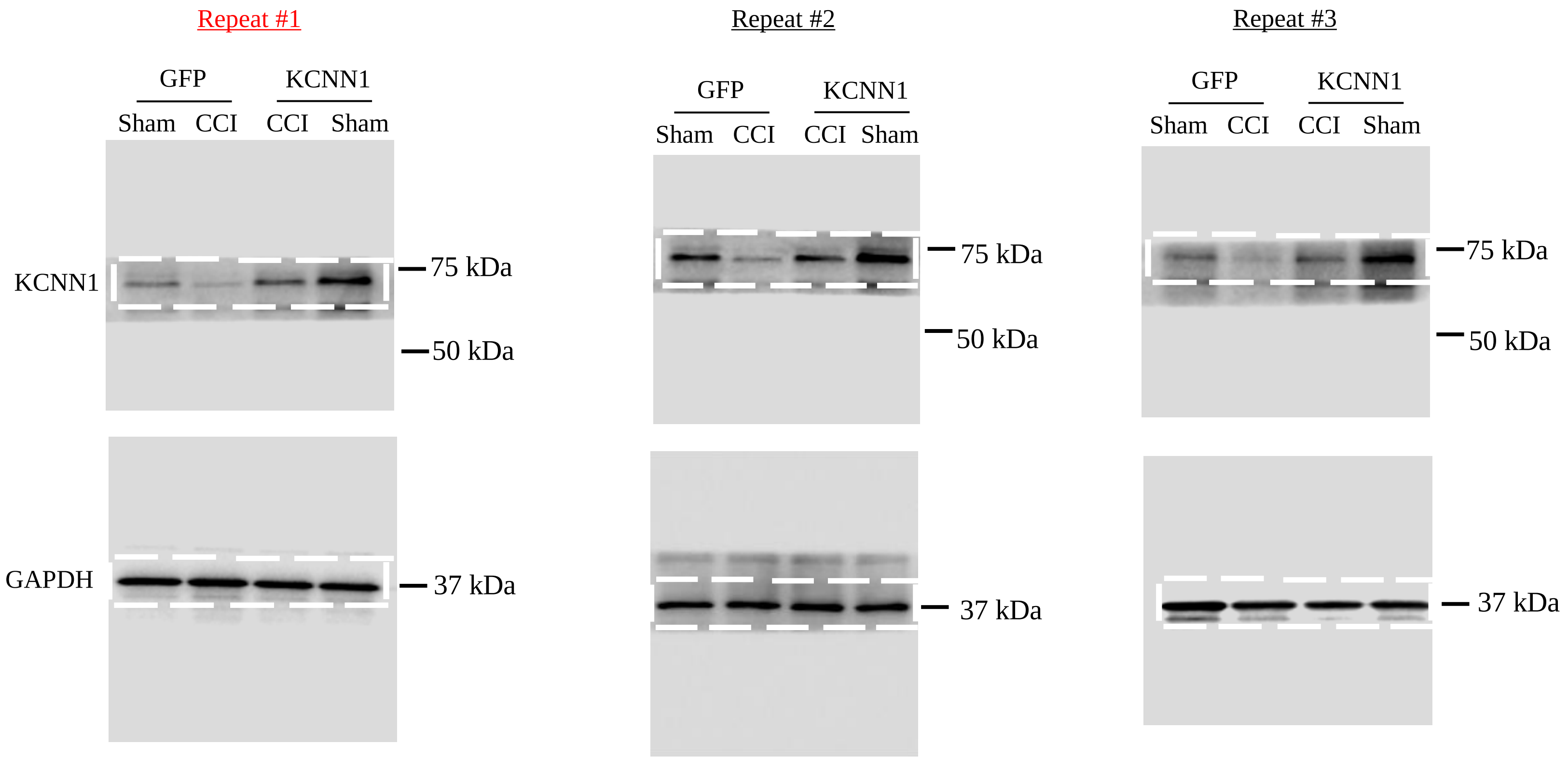

Full unedited gels for supplementary Fig. 3K

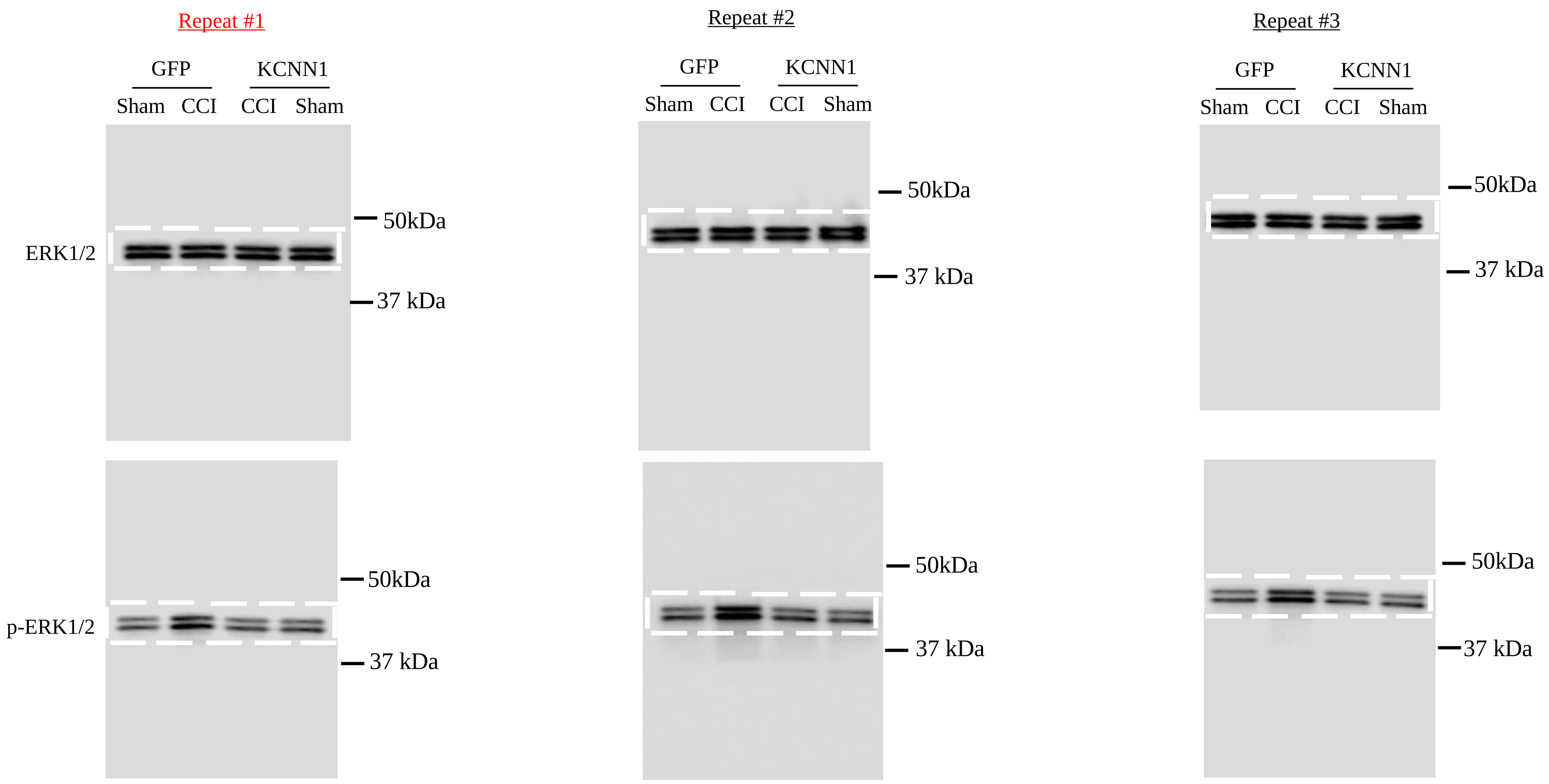

Full unedited gels for supplementary Fig. 3K

Repeat #1

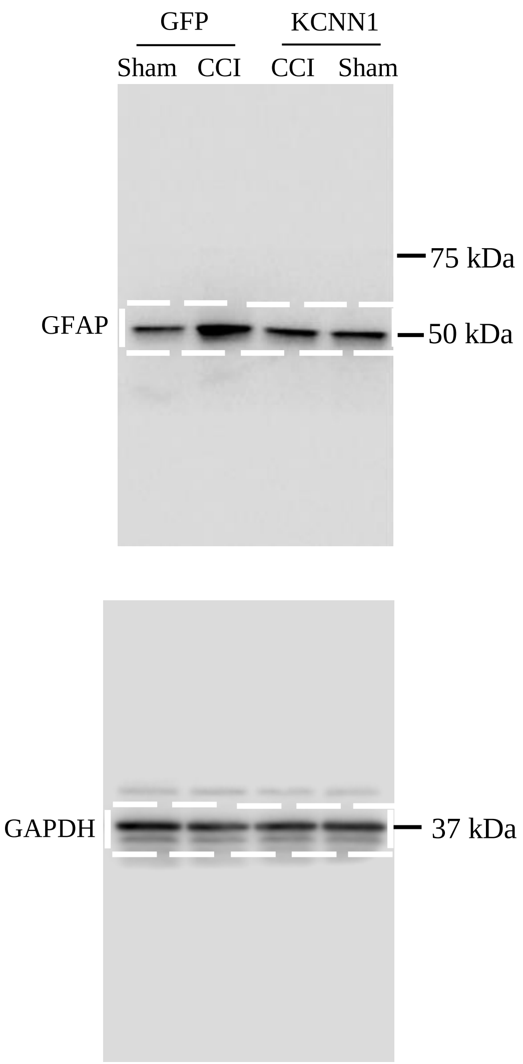

Repeat #2

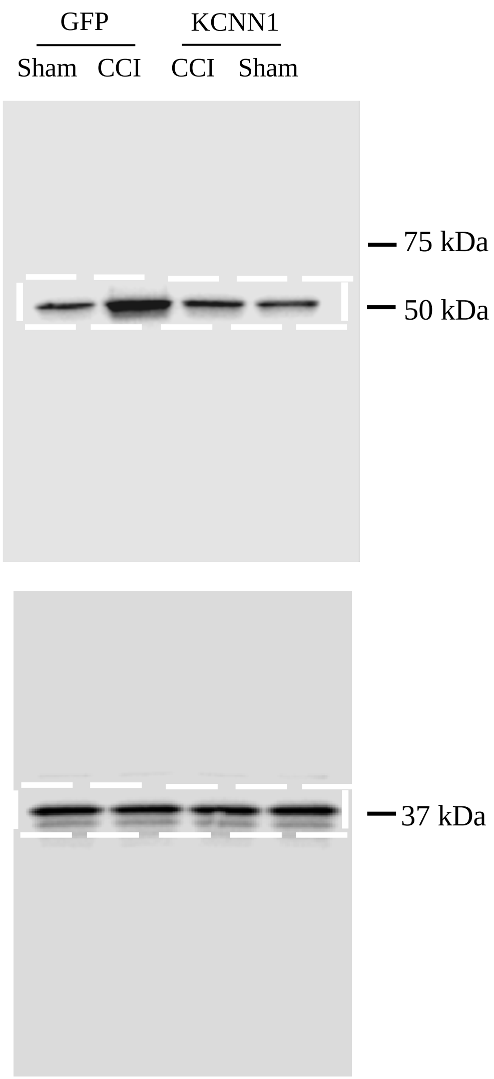

Repeat #3

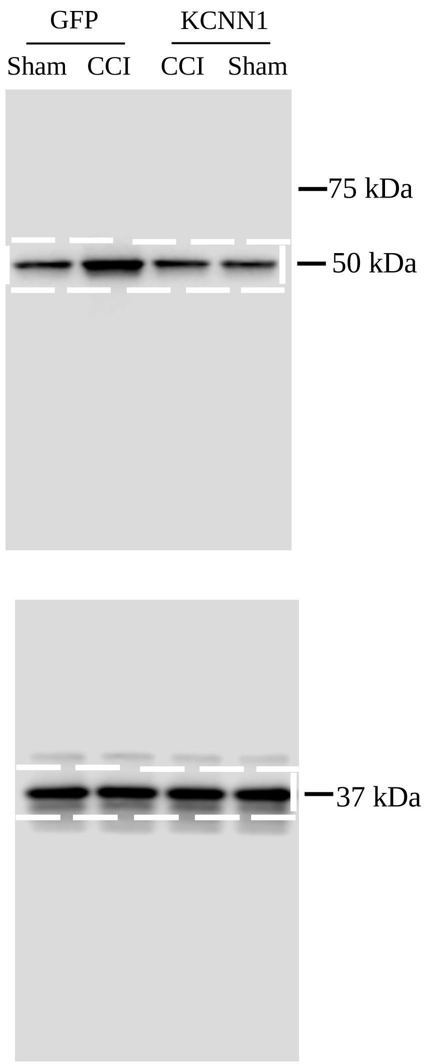

Full unedited gels for supplementary Fig. 3L

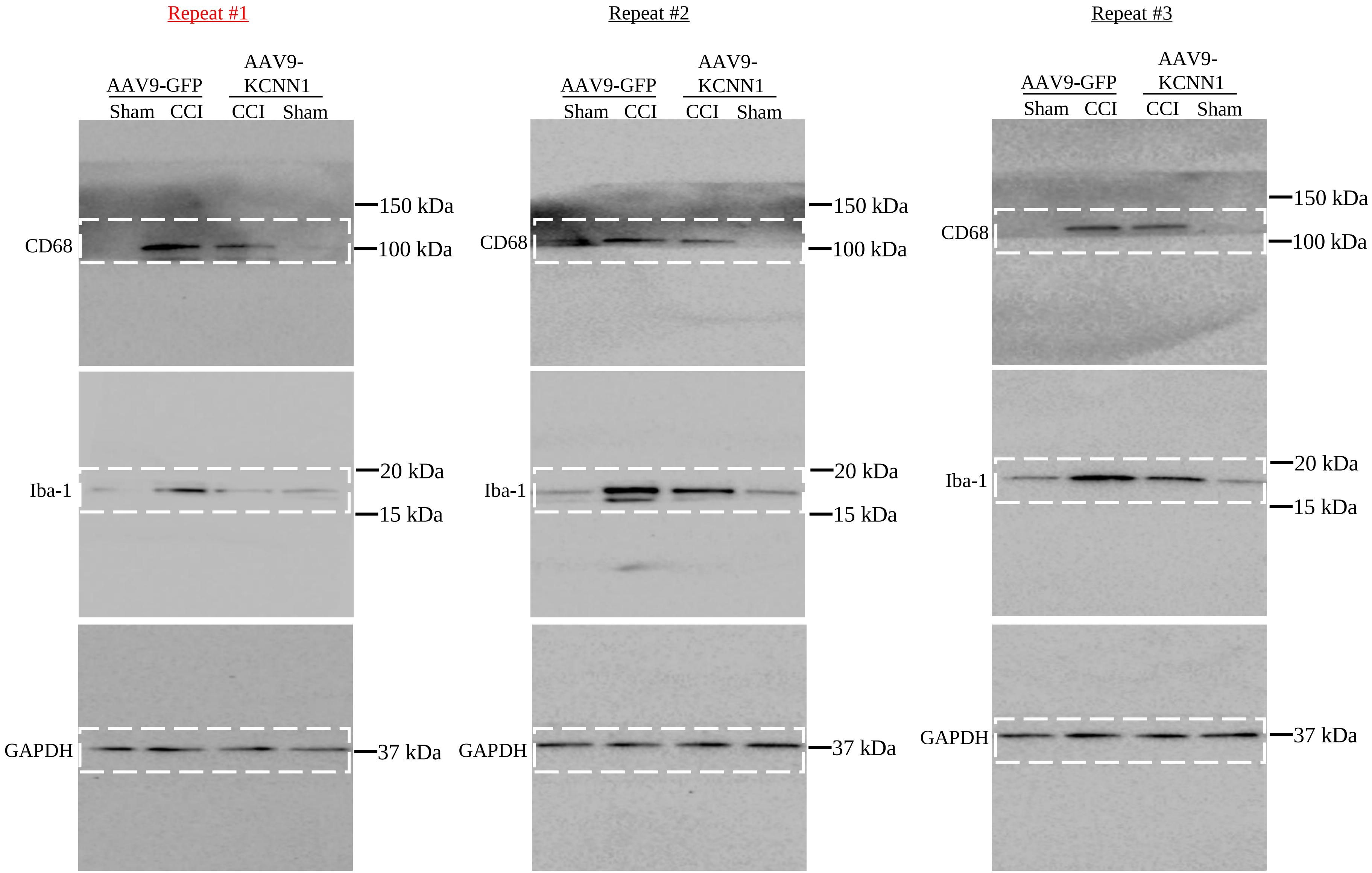

Full unedited gels for supplementary Fig. 4A

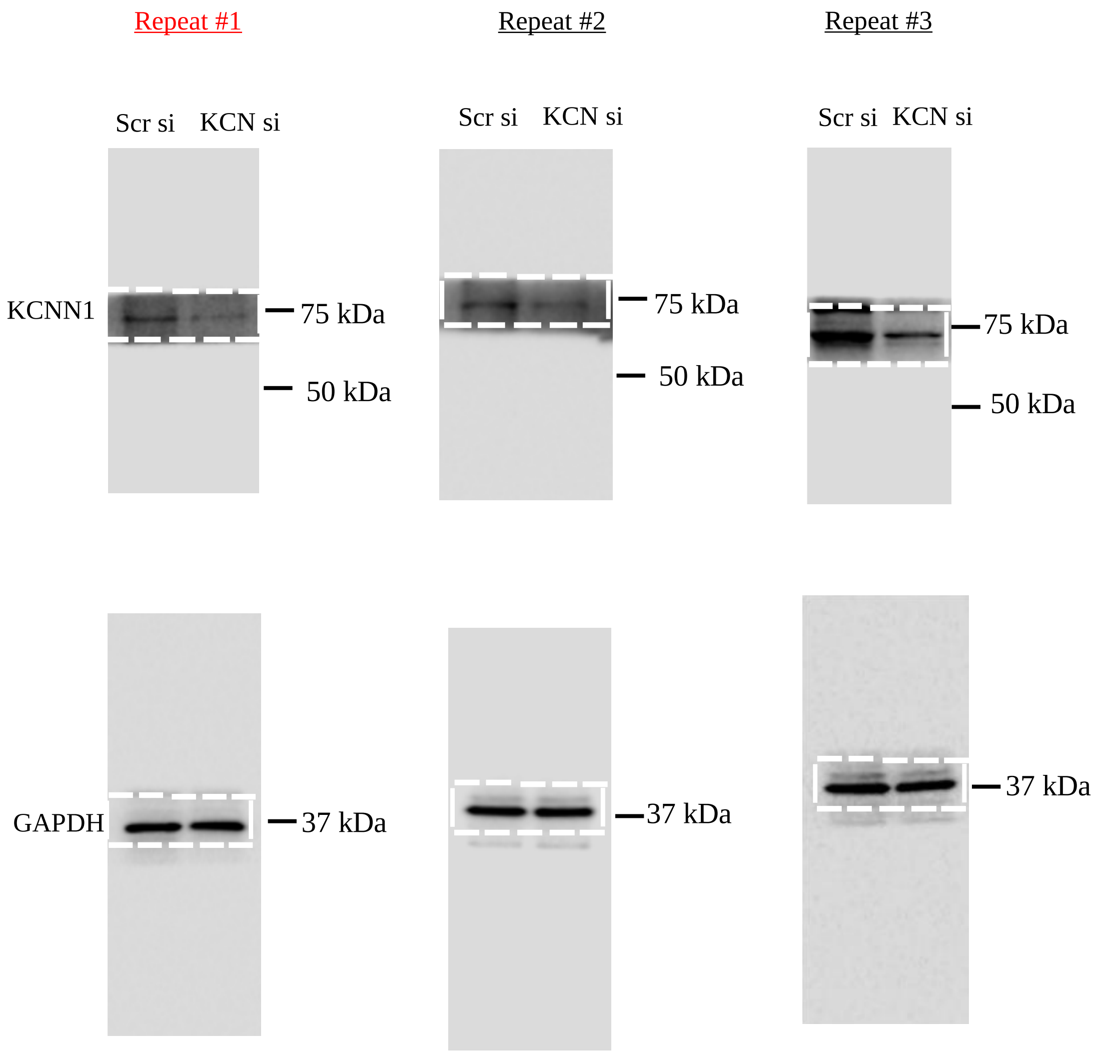

Full unedited gels for supplementary Fig. 4F

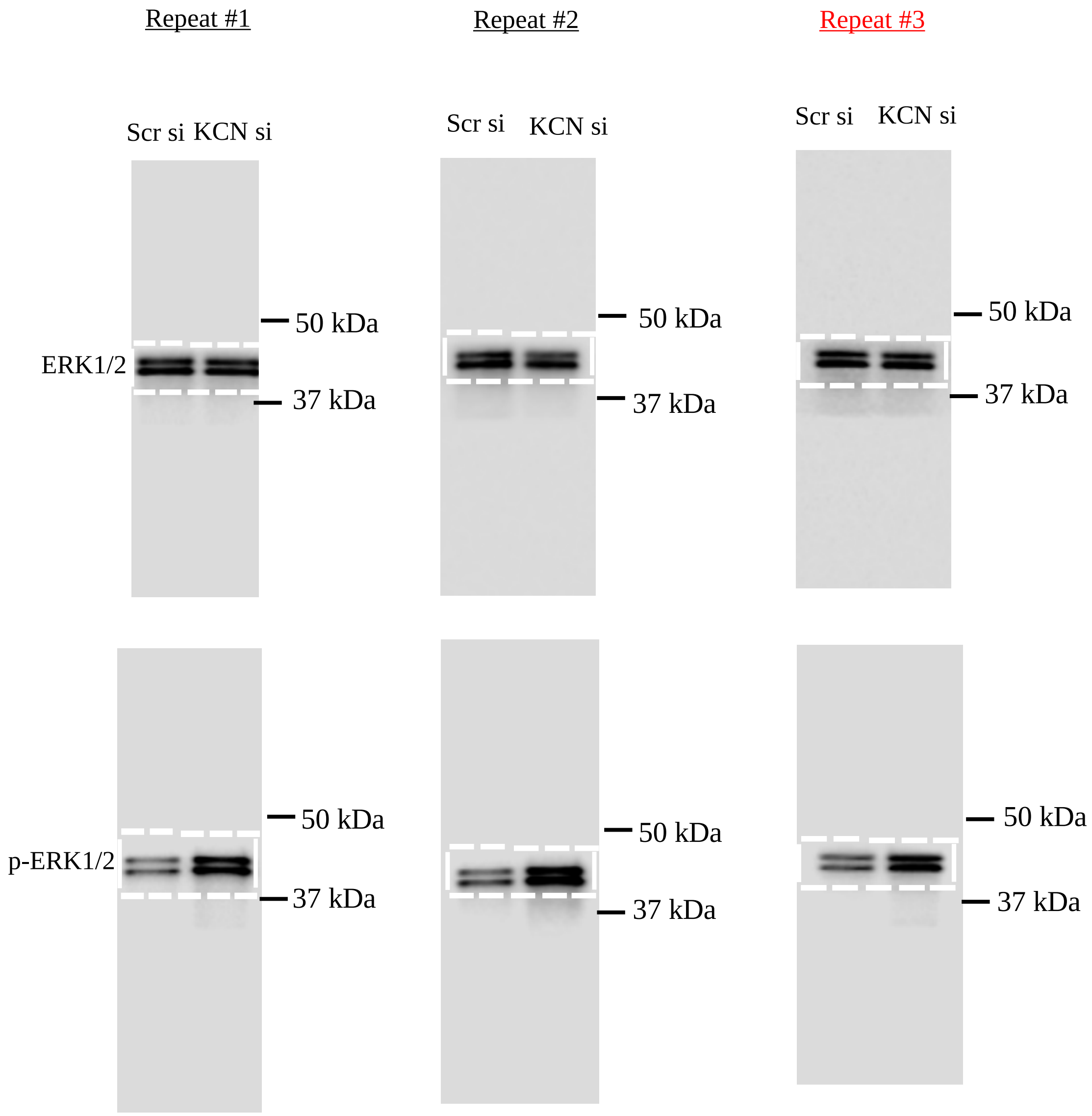

Full unedited gels for supplementary Fig. 4F

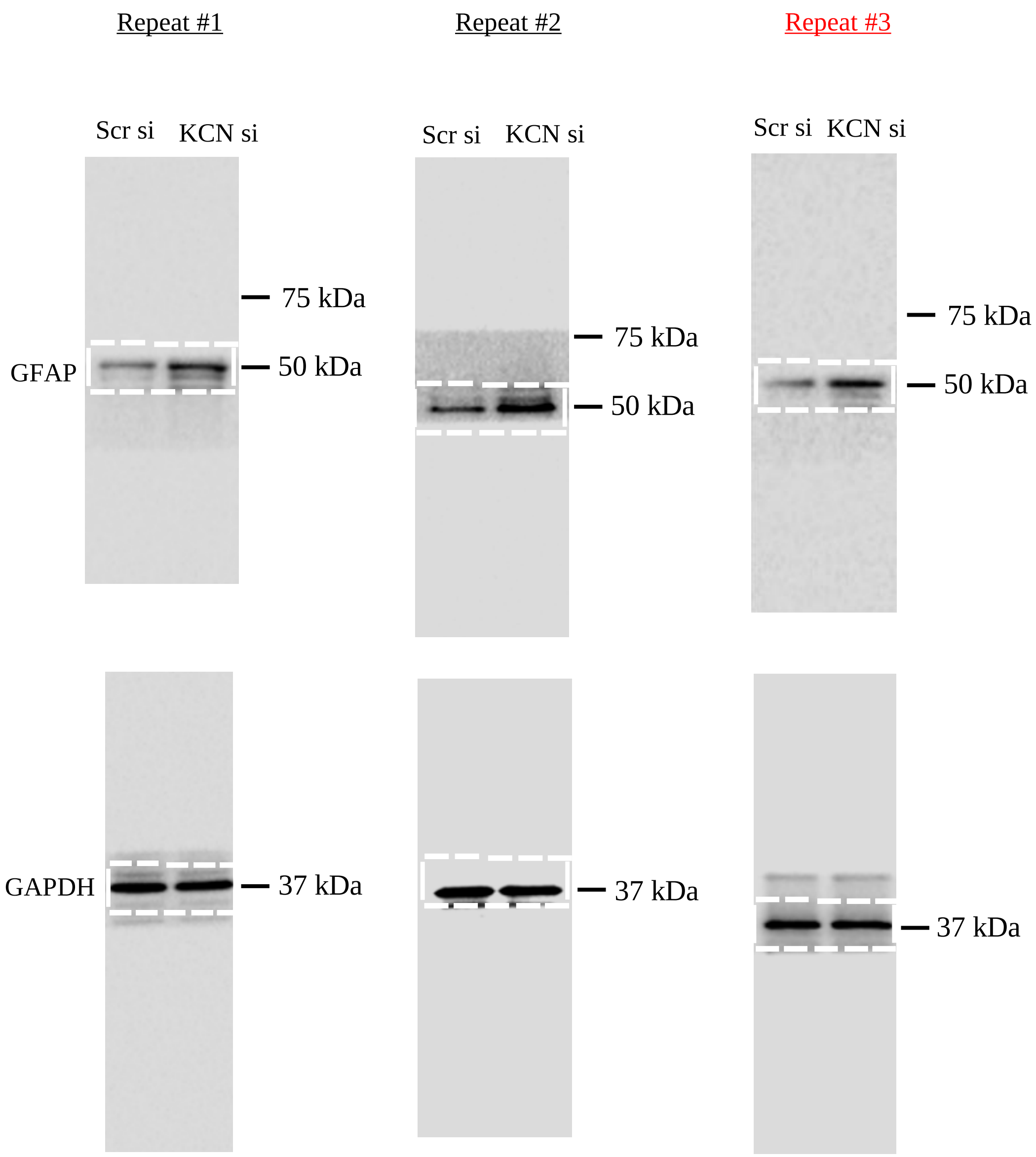

Full unedited gels for supplementary Fig. 4G

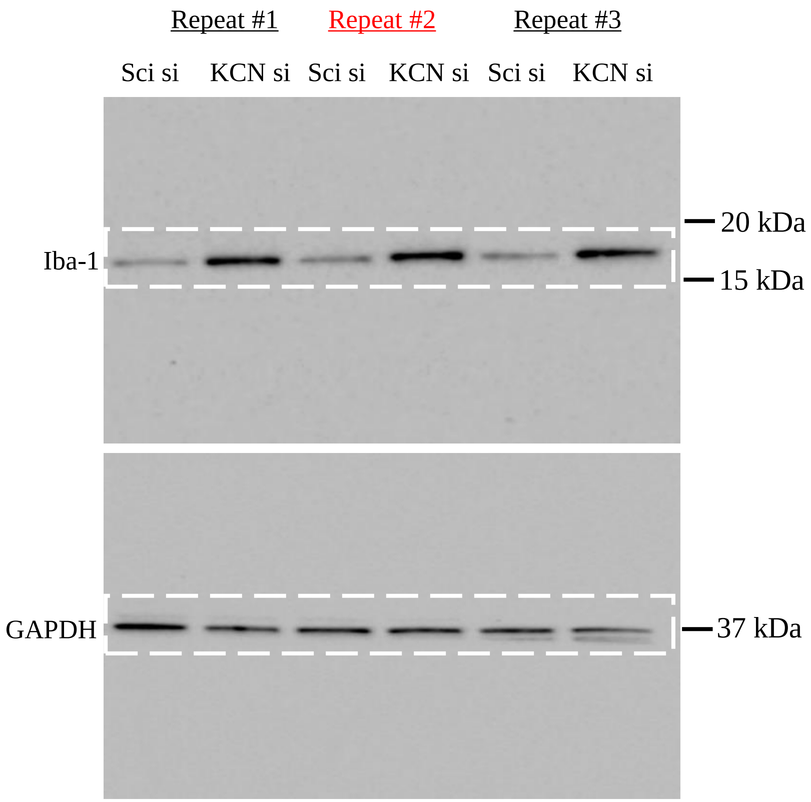

Full unedited gels for supplementary Fig. 6G

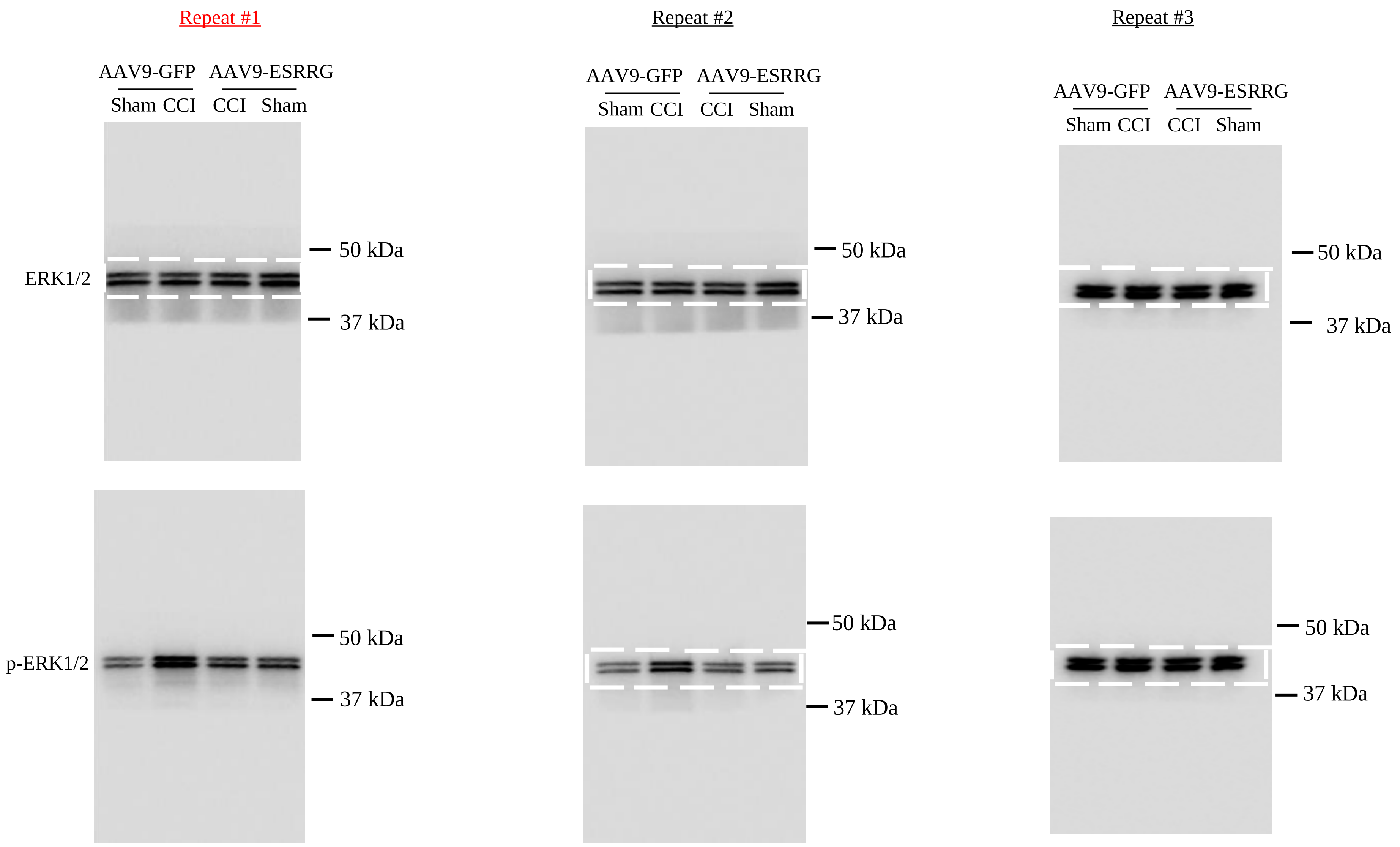

Full unedited gels for supplementary Fig. 6G

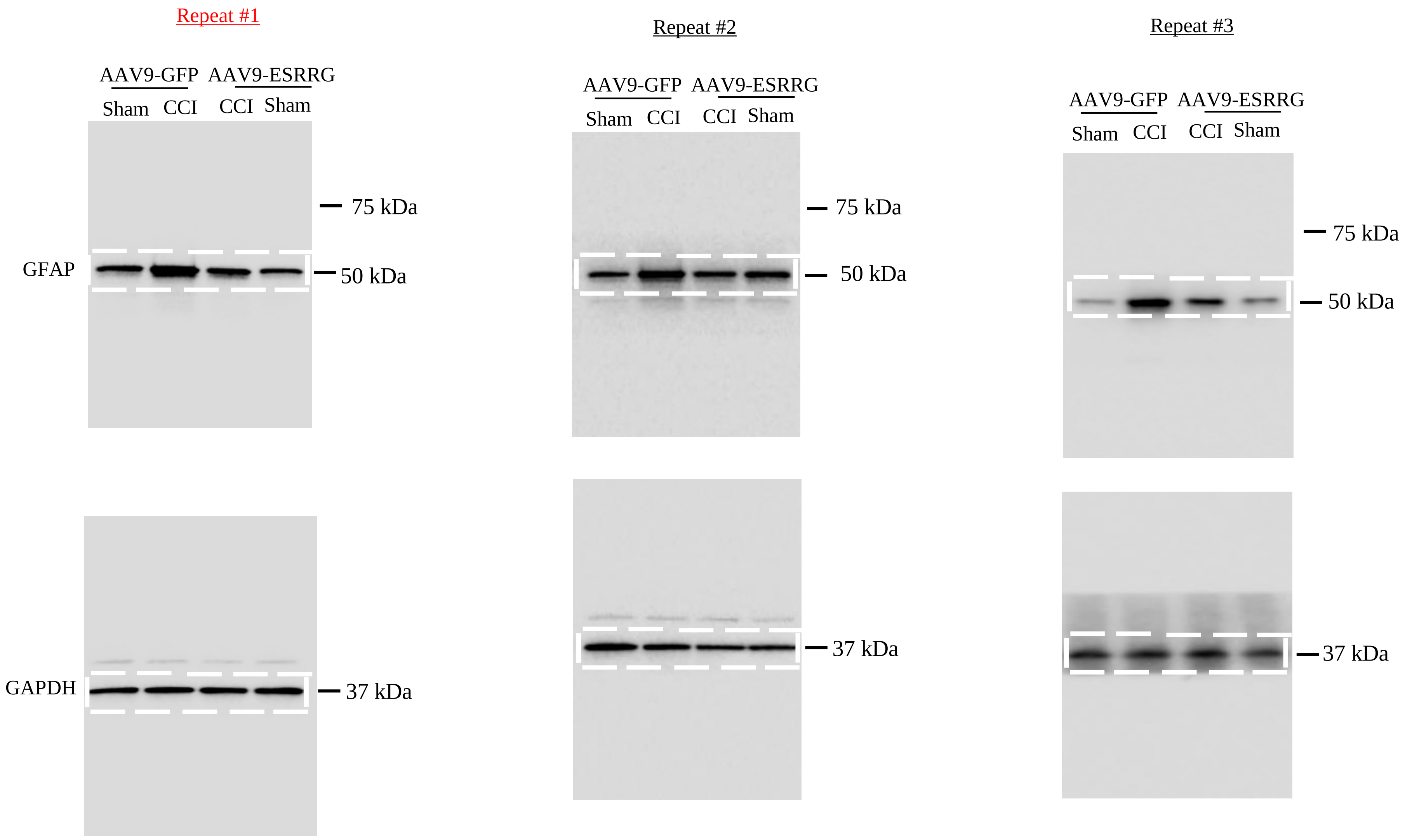

Full unedited gels for supplementary Fig. 6I

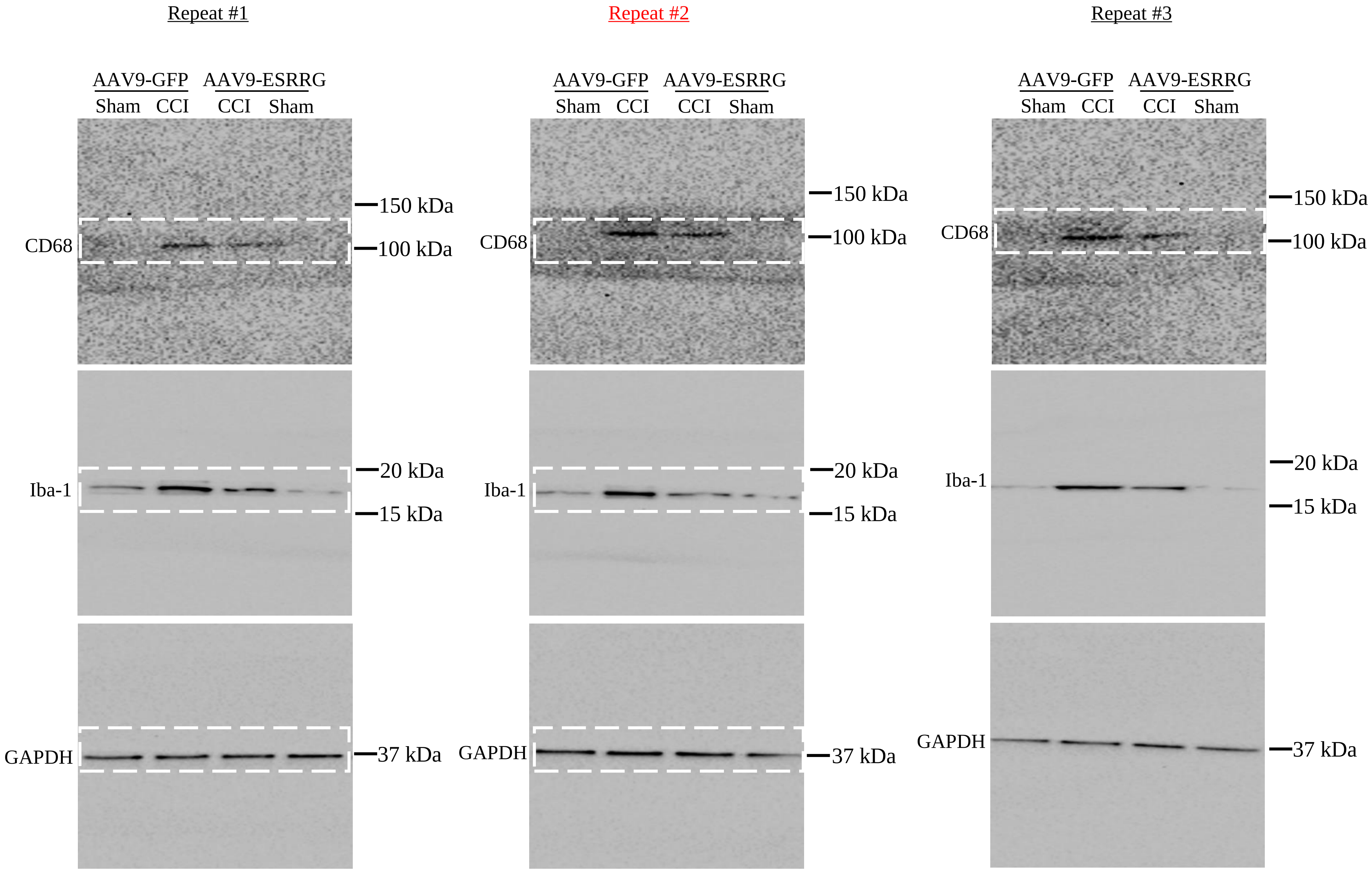

Full unedited gels for supplementary Fig. 7D

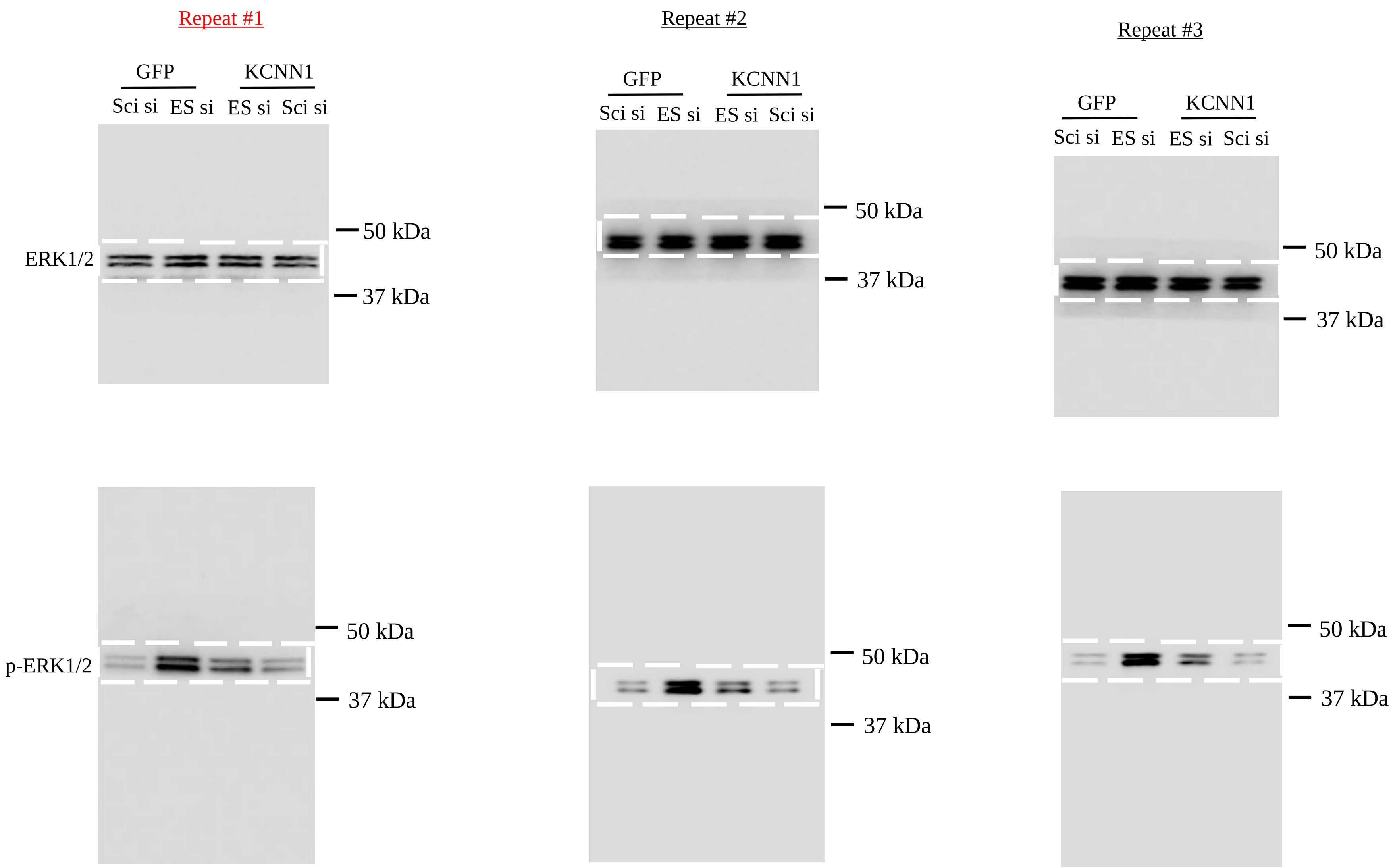

Full unedited gels for supplementary Fig. 7D

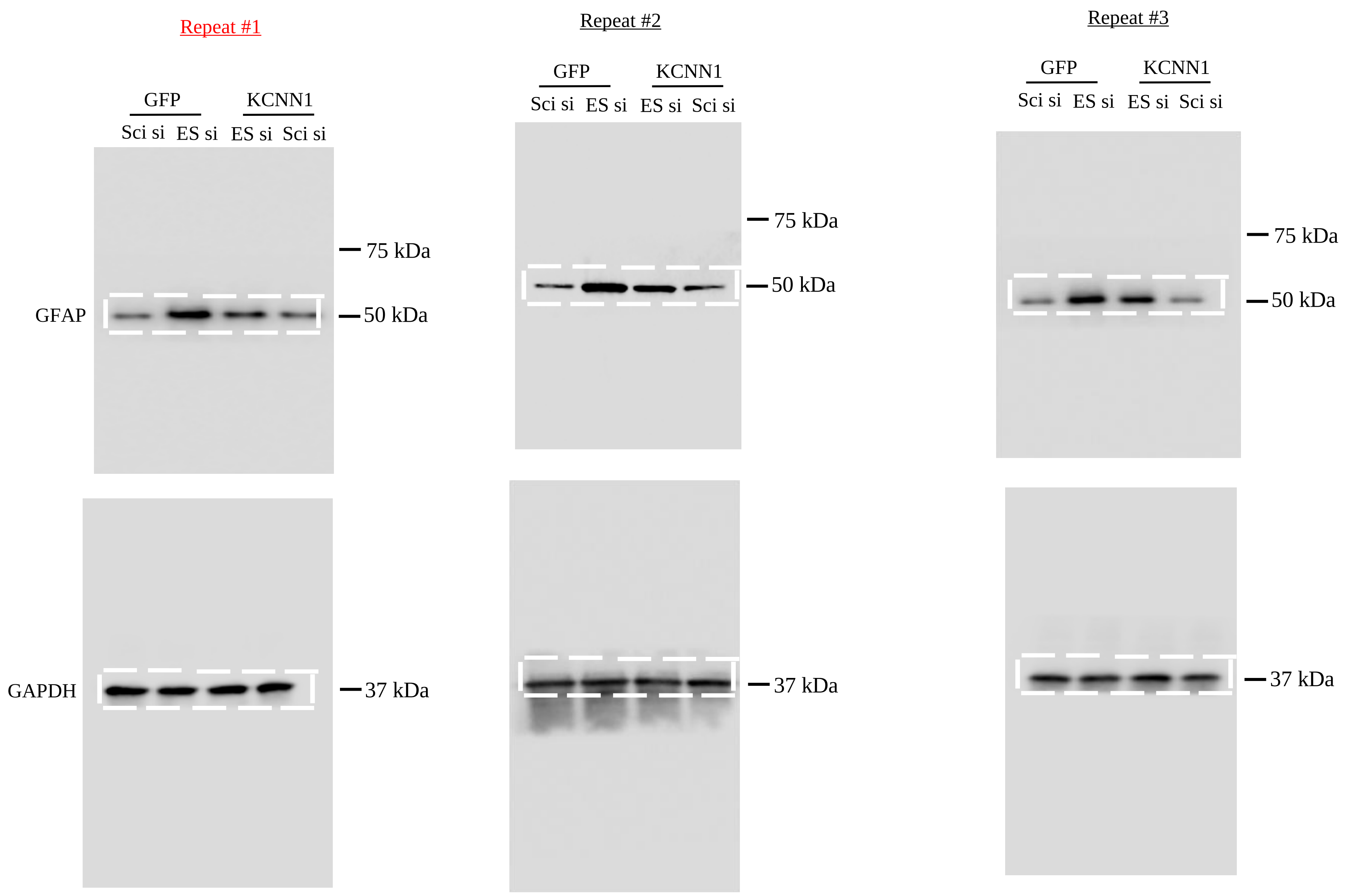

Full unedited gels for supplementary Fig. 7E

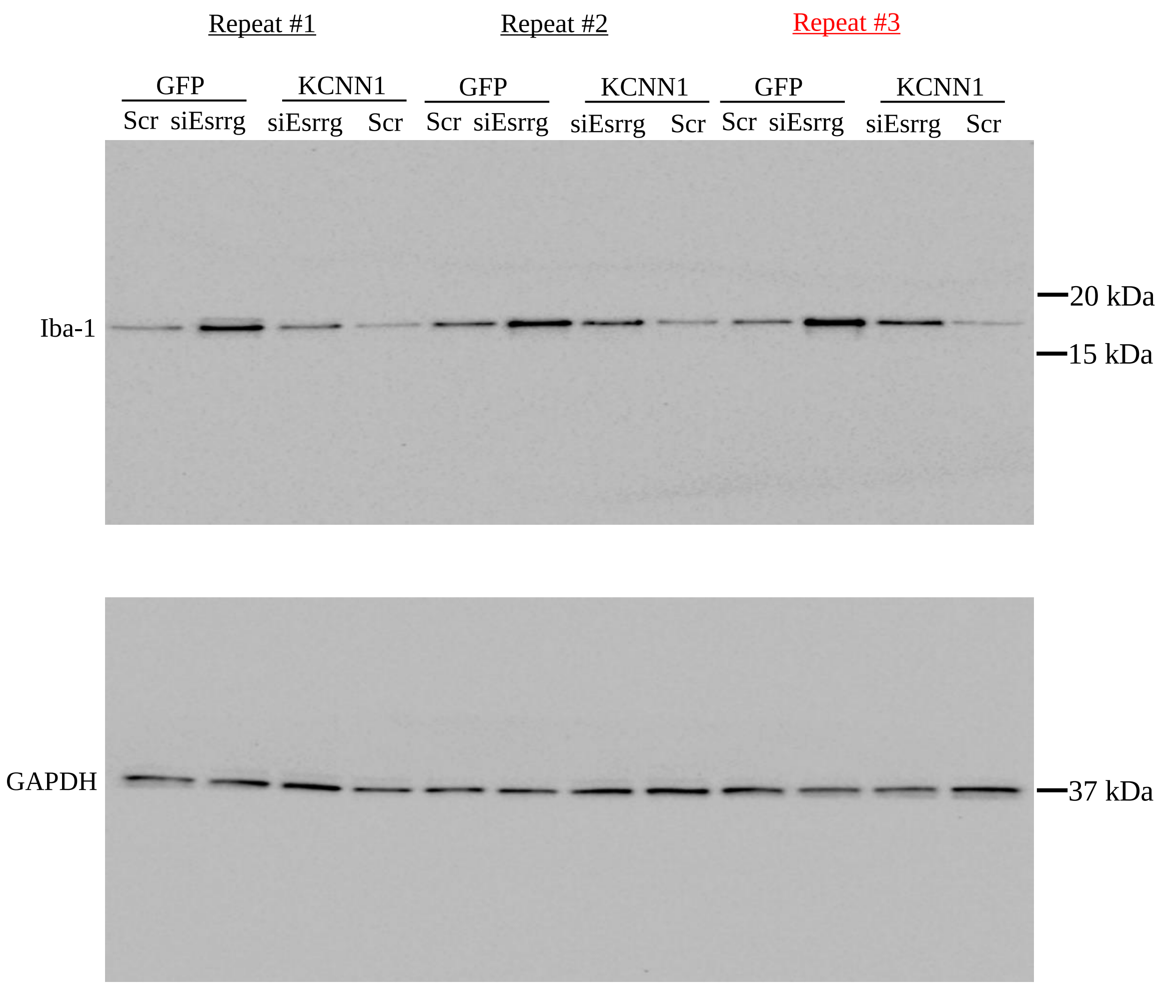

Supplement: Unedited blot and gel images [file jciinsight-9-180085-s089.pdf]
